# Supplementary material for: Dynamic cybergenetic control of bacterial co-culture composition via optogenetic feedback
Source: Nat Commun. 2022 Aug 16;13:4808. doi: 10.1038/s41467-022-32392-z (PMC9381578; doi:10.1038/s41467-022-32392-z)
Supplement: Supplementary file 1 — Supplementary Information [file 41467_2022_32392_MOESM1_ESM.pdf]

Supplementary Information for

Dynamic control over the composition of bacterial co-cultures via optogenetic feedback

Joaquín Gutiérrez Mena, Sant Kumar, Mustafa Khammash*

*Corresponding author. Email: mustafa.khammash@bsse.ethz.ch

This PDF file includes:

- Supplementary Text Sections 1-3
- Supplementary Figures 1-23
- Supplementary Tables 1-2

1 Construction of *evotron* framework

1.1 eVOLVER sleeve re-design

Hardware

We re-designed the eVOLVER sleeve as shown in Supplementary Figure 21 and Supplementary Figure 22 to accommodate new tube holder and vial cap designs resulting in stable and consistent OD sensor measurements (Supplementary Figure 3). We also integrated one blue LED (465nm, superbrightleds: RL5-B08-360) per sleeve to facilitate optogenetic stimulation of target cell culture when placed in the sleeve. This blue LED was connected to the vial electronic board by replacing the 90 degree photodiode (IR detector), shown in Supplementary Figure 22b and Supplementary Figure 19a. Since, our experiments involved cell cultures maintained at lower cell densities (OD < 0.2), readings from 135 degree photodiode (IR detector) were enough to provide accurate OD measurements (thus, 90 degree photodiode was not required). Further, we also replaced the default M6 plugin (on the eVOLVER motherboard) with a PWM board(1) to power this additional LED, and re-programmed UC2 arduino (SAMD21) to provide software control access for changing LED intensity in real-time during optogenetic experiments.

Software

All CAD design files, codes and routines are available in this GitHub repository:
`https://github.com/santkumar/evotron.git` (Zenodo DOI 10.5281/zenodo.6908131)

1.2 Automated sampling platform design

Hardware

We designed our automated sampling and measurement framework around Opentrons OT-2 liquid handling robot. The modified eVOLVER platform was placed inside the Opentron OT-2 robot replacing its deck, as can be seen in Supplementary Figure 23a. Three cleaning solution bottles (1-H_2O, 2-Bleach (2%), 3-H_2O) were also placed on the OT-2 deck for cleaning the sample path after every sampling step. A sampling-needle was fixed onto the OT-2 pipette head by using a custom-designed 3D printed needle holder (Supplementary Figure 23a).

As seen in Supplementary Figure 23b, the sampling needle was attached to a flexible silicone tubing which provided a route for the sample to move into the flow-cytometer (Cytoflex S, Beckman Coulter) sampling vial via a peristaltic sampling pump (Intellicyt peristaltic pump). Another tubing and waste-removal pump was set up to remove the residual sample from flow-cytometer sampling vial after measurement. A controller (Arduino Mega 2560) was incorporated in the framework providing software control access of the OT-2 robot (serial communication), sampling and waste-removal pumps (TTL signaling). The control computer was connected to the OT-2 and eVOLVER platforms via WiFi communication, and was set up to communicate with the arduino controller serially (Figure 3b).

Software

All CAD design files, codes and routines are available in this GitHub repository:
`https://github.com/santkumar/evotron.git` (Zenodo DOI 10.5281/zenodo.6908131)

Pseudo Code

These are the synchronized steps performed during an automated sampling event:

1. Start the sampling pump.
2. Move the OT-2 pipette head to a desired eVOLVER sleeve location (where our target cell culture is maintained within OD range 0.1-0.15 in a glass vial).
3. Lower the OT-2 pipette head so that the sampling-needle is dipped into the cell culture, wait for 3 seconds (for the sampling pump to extract ∼0.5ml of culture into the sampling tubing), and then move it up.
4. Wait for 40 seconds so that the extracted sample is pulled and completely discharged into the flow-cytometer sampling vial, then stop the sampling pump.
5. Start the flow-cytometer measurement.
6. Once measurement is done, start the waste-removal pump and wait for 10 seconds so that the residual sample is completely pumped out of the flow-cytometer samling vial, and then stop the waste-removal pump.
7. Start the sampling pump.
8. Move the OT-2 pipette head to the first cleaning solution (sterile H_2O) bottle location.
9. Lower the OT-2 pipette head so that the sampling-needle is dipped into the cleaning solution, wait for 4 seconds (for the sampling pump to extract ∼0.7ml of cleaning solution into the sampling tubing), and then move it up.
10. Wait for 40 seconds so that the extracted cleaning solution is pulled and completely discharged into the flow-cytometer sampling vial, then stop the sampling pump.
11. Start the waste-removal pump and wait for 10 seconds so that the cleaning solution is completely pumped out of the flow-cytometer samling vial, and then stop the waste-removal pump.
12. Move the OT-2 pipette head to the second cleaning solution (2% bleach solution) bottle location.
13. Lower the OT-2 pipette head so that the sampling-needle is dipped into the bleach solution, wait for 6 seconds (for the sampling pump to extract ∼1ml of bleach solution into the sampling tubing), and then move it up.
14. Wait for 40 seconds so that the extracted bleach solution is pulled and completely discharged into the flow-cytometer sampling vial, then stop the sampling pump.
15. Wait for 10 seconds so that the bleach solution has sufficient time to disinfect the sampling tubing and the flow-cytometer sampling vial.
16. Start the waste-removal pump and wait for 10 seconds so that the bleach solution is completely pumped out of the flow-cytometer samling vial, and then stop the waste-removal pump.
17. Move the OT-2 pipette head to the third cleaning solution (sterile H_2O) bottle location.
18. Lower the OT-2 pipette head so that the sampling-needle is dipped into the cleaning solution, wait for 8 seconds (for the sampling pump to extract a bit more than 1ml of cleaning solution into the sampling tubing), and then move it up.
19. Wait for 40 seconds so that the extracted cleaning solution is pulled and completely discharged into the flow-cytometer sampling vial, then stop the sampling pump.
20. Start the waste-removal pump and wait for 10 seconds so that the cleaning solution is completely pumped out of the flow-cytometer samling vial, and then stop the waste-removal pump.
21. The automated sampler is now ready to perform the next sampling.

Note:

- During a closed-loop control experiment, the feedback controller computation routine is run on measurements just after the flow-cytometer measurement is done in step 6. Once the computation is over, the LED (integrated on the respective eVOLVER sleeve of the target cell culture vial) stimulation intensity is set as per the value computed by this controller. These controller computations are carried out in parallel with cleaning steps 7-20.
- When performing all the above mentioned steps, a single automated sampling event takes approx. 5 and half minutes to finish. This was the limiting factor in deciding how many culture vials (5 in our case even though the eVOLVER platform has 16 parallel culture sleeves) can be maintained and experimented with in parallel, given that the frequency of control input (light intensity) update in our co-culture composition control experiments was set to be once every 30 minutes.
- **Comparison with ReacSight framework (2)**: A recently published article (2) proposed a similar hardware-software framework centered around a pipetting robot (Opentrons OT-2) enabling automated sampling and measurement capabilities. In contrast to our *evotron* framework, this ReacSight approach involves moving culture samples via sampling lines to the OT-2 deck and then transferring them to a measurement device (positioned within the OT-2 pipette head accessible region) using default pipettes available on OT-2. To achieve automated sampling, measurement and reactive control capability, one can adopt any of the two (*evotron* and ReacSight) strategies depending on the application, culture platforms, and measurement devices. Our *evotron* framework facilitates integration of any suitable measurement device, and does not require positioning them within the OT-2 pipette head accessible region, which is required in the ReacSight setup. On the other hand, we place the culture platform on the deck of the OT-2 robot, which is placed outside of the OT-2 in ReacSight approach.

2 Formulation of the mathematical modeling framework

Unit conversion

We note that the cellular protein density of *E. coli* is approximately independent of the growth rate (3). Because of this, we can estimate a factor that converts protein concentrations into fractions of the proteome and viceversa. We define the proteome fraction of a given protein type X as

$$\Phi_X = \frac{M_X[\text{aa}]}{M_{cell}[\text{aa}]} \tag{1}$$

where M_X is the mass of all protein copies of type X and M_{cell} is the mass of all proteins in the cell, both in units of the average mass of an amino acid. Denoting the mass of a single copy of X by n_X and the number of protein copies by N_X, we can relate Φ_X to the concentration c_X in the following way:

$$\begin{aligned}\Phi_X &= \frac{M_X[\text{aa}]}{M_{cell}[\text{aa}]} = n_X[\text{aa}]\,\frac{N_X[\text{molecules}]}{M_{cell}[\text{aa}]} \\ &= n_X[\text{aa}]\,\frac{V_{cell}[\text{fL}]}{M_{cell}[\text{aa}]}\,\frac{N_X[\text{molecules}]}{V_{cell}[\text{fL}]} \\ &= n_X[\text{aa}]\,\rho_{cell}^{-1}\,c_X[\frac{\text{molecules}}{\text{fL}}]\end{aligned} \tag{2}$$

where we have denoted the cell volume by V_{cell} and introduced the cellular protein density, which is approximately (4) $\rho_{cell} \approx \frac{2\cdot 10^9\,\text{aa}}{1\,\text{fL}} = 2\cdot 10^9\,\frac{\text{aa}}{\text{fL}}$. In the following sections, we use $\frac{n_X}{\rho_{cell}}$ as an inter-conversion factor between protein concentrations and proteome mass fractions.

Proteome partition model of bacterial physiology

The framework for host-aware modeling of synthetic genetic circuits we present in this study is based on the proteome-partition model put forward by Scott *et al.*(5). In this section, we provide a brief summary of their model, focusing on the equations that are central to the development of our approach. For further details and derivations of these equations from first principles, we refer the reader to the original publications (5, 6). For examples on how these types of models have been applied to a range of biological problems, see e.g. (7–9).

The model proposed by Scott *et al.* attempts to explain key observations about how the macromolecular composition of *E. coli* varies when the growth rate of the cell changes. The composition of the cell, i.e. the relative amounts of its component proteins, is dictated by its gene expression profile. Therefore, the relation between the composition and the growth rate is ultimately a connection between gene expression and the physiology of the cell, for which growth rate is the most direct readout. The laws governing this relation are of high importance for the prediction of how synthetic genetic circuits behave in the cellular context.

Despite the complexity of a cell, the empirical link between aspects of its macromolecular composition and its growth rate turns out to be remarkably simple. At the core of the model of Scott *et al.* are two linear relations that were determined experimentally, which relate the fraction of the proteome made up by ribosomes and the growth rate (depicted schematically in Figure 4a). The first one applies when the growth rate is changed by modulating the quality of nutrients in the media and it implies a positive correlation between ribosomal content and growth rate:

$$\lambda = \gamma_0 \left(\Phi_R - \Phi_{R_0}\right) \tag{3}$$

where λ represents the growth rate, Φ_R, the ribosomal mass fraction and Φ_{R_0}, the Y-offset extrapolated from experiments which corresponds to ribosomes that are not actively engaged in translation. The proportionality constant γ_0 can be shown to correspond to the translation rate.

The second linear relation is revealed when growth rate is modulated by translation inhibition, e.g. by exposing *E. coli* to increasing concentrations of chloramphenicol in the presence of saturating nutrient availability. In that case, the cell upregulates its ribosome content at lower growth rates, in response to the increasing inhibition of translation brought about by the antibiotic:

$$\lambda = \nu \left(\Phi_R^{\max} - \Phi_R\right). \tag{4}$$

where, ν characterizes the quality of the available nutrients and Φ_R^{max} is again the Y-offset, i.e. the maximal ribosome content that is theoretically possible.

Scott *et al.* propose that the simplest way to account for these linear growth laws is to assume a partition of the proteome in three fractions. The existence of a constant fraction Φ_Q, that does not vary as the growth rate changes, is deduced from the fact that the ribosomal fraction cannot be increased further than a maximum of $\Phi_R^{\max} = 1 - \Phi_Q$. This fixed fraction is hypothesized to represent house-keeping proteins, whose concentration is kept constant by negative feedback mechanisms. The ribosomes and associated proteins required for translation constitute a further significant fraction of the proteome, Φ_R. It's magnitude is closely associated to the growth rate of the cell, as described by the growth laws, equations 3 and 4. Finally, since Φ_Q is fixed, there needs to be a third fraction that accommodates for changes in Φ_R. This variable fraction,

$$\Phi_P = 1 - \Phi_Q - \Phi_R = \Phi_R^{\max} - \Phi_R = \frac{\lambda}{\nu} \tag{5}$$

is interpreted as the group of proteins responsible for catabolism, as well as other proteins whose expression is unregulated, i.e. not subject to any of the homeostatic mechanisms that stabilize components of the Q-fraction. It

follows the exact opposite behavior to the ribosomal fraction Φ_R, i.e. decreasing with increasing nutrient quality and decreasing with increasing inhibition of ribosomes. Crucially for the derivation of our model framework below, constitutive genes were shown to belong to this category(5) (Supplementary Figure 20).

The growth laws, equations 3 and 4, together with the constraint of having a finite proteome size,

$$\Phi_Q + \Phi_R + \Phi_P = 1 \tag{6}$$

are solved by Scott *et al.* to arrive at expressions for the growth rate and ribosomal fraction, that depend only on three parameters: the nutritional capacity ν, the translational capacity γ_0 and the maximally-possible ribosomal fraction $\Phi_R^{\max}$.

$$\lambda\left(\gamma_0, \nu, \Phi_R^{\max}\right) = \left(\Phi_R^{\max} - \Phi_{R_0}\right)\frac{\gamma_0\,\nu}{\gamma_0 + \nu} \tag{7}$$

$$\Phi_R\left(\gamma_0, \nu, \Phi_R^{\max}\right) = \left(\Phi_R^{\max} - \Phi_{R_0}\right)\frac{\nu}{\gamma_0 + \nu} + \Phi_{R_0} \tag{8}$$

Finally, Scott *et al.* also discussed what would happen if non-toxic, exogenous proteins are expressed in the cell. Exogenous gene expression would introduce a further proteome fraction, Φ_S (S for *synthetic*), which results in growth defects because it restricts the fraction of the proteome that is available for ribosomes and catabolic proteins (effectively decreasing the value of $\Phi_R^{\max}$). With the extra proteome fraction, the finite proteome constraint becomes

$$\Phi_Q + \Phi_R + \Phi_P + \Phi_S = 1 \tag{9}$$

which leads to equations for the growth rate and ribosomal fraction that explicitly depend on Φ_S:

$$\lambda\left(\gamma_0, \nu, \Phi_R^{\max}, \Phi_S\right) = \left(\Phi_R^{\max} - \Phi_{R_0} - \Phi_S\right)\frac{\gamma_0\,\nu}{\gamma_0 + \nu} \tag{10}$$

$$\Phi_R\left(\gamma_0, \nu, \Phi_R^{\max}, \Phi_S\right) = \left(\Phi_R^{\max} - \Phi_{R_0} - \Phi_S\right)\frac{\nu}{\gamma_0 + \nu} + \Phi_{R_0} \tag{11}$$

Note that this assumes a synthetic proteome fraction that is fixed, as is the case when a neutral protein product is expressed constitutively. Furthermore, Φ_S enters the equations as an extra parameter. Scott *et al.* express an exogenous protein in *E. coli* and measure the fraction of the proteome occupied by that protein. They show that the relation between that fraction and the growth defects observed can be quantitatively described by equation 10.

The parameters ν, γ_0 and $\Phi_R^{\max}$ were estimated by Scott *et al.* for a wide range of conditions encompassing cells growing in different media and with different concentrations of chloramphenicol. Most studies on synthetic genetic circuits in *E. coli* are conducted in a laboratory setting under comparable conditions, e.g. the performance of a circuit is commonly evaluated in cells that grow exponentially at 37°C with saturated availability of oxygen and a well-defined growth medium. Under such conditions, γ_0 and $\Phi_R^{\max}$ do not vary much, which makes it possible to use the values estimated in (5). The above equations can then be used to predict growth defects due to burden, provided that the amount of synthetic proteins being expressed is known (with respect to the total protein mass of the cell) and the single missing parameter, ν, is determined. ν can be estimated by measuring the growth rate of the strain of interest in the chosen media.

Host-aware modeling framework for synthetic genetic circuits

Expression of heterologous genes consumes cellular resources and reduces the growth rate of cells. In turn, limitations in the cellular gene expression machinery impact the performance of synthetic circuits(10). Here, we develop a modeling framework that captures this two-way interference and that can be seamlessly incorporated into conventional ODE models with little extra complexity and adding no extra free parameters.

In the proteome allocation model of Scott *et al.* summarized above, equations 10 and 11 quantify the impact of expressing heterologous genes on the cellular resources (Φ_R) and the growth rate (λ). They are valid for balanced exponential growth. However, modelers are often interested in understanding the dynamics of circuits and not only their steady-state behavior. Moreover, the proteome fraction occupied by the circuit, Φ_S, which enters the equations as a free parameter, cannot be determined experimentally in most practical cases. The equations also do not explicitly quantify the effect that the limited pool of cellular resources has on the performance of a circuit.

Derivation of the framework

In the following, we assume that the relations presented in the previous section also apply to the transient dynamics of a synthetic genetic circuit and not only at steady-state. To address the other issues, we begin by considering the simplest case of a circuit composed by a single, constitutively-expressed gene, x. The study by Scott *et al.* demonstrated that this type of unregulated expression would fall under the P-type proteome fraction(5, 8), which follows the opposite rules that apply to the ribosomal fraction (eq. 5). This means that an appropriate host-aware model of this simple circuit should recover two general trends:

1. the proteome fraction of X should decrease linearly with growth rate, when growth is modulated by nutrient quality
2. The proteome fraction of X should increase linearly with growth rate, when growth is modulated by translational capacity and the slope of this increase should depend on the nutrient quality.

Conventional models of constitutive expression represent transcription and translation as birth-death processes:

$$\begin{aligned} \frac{dm_X}{dt} &= \omega - \delta_m \, m_X \\ \frac{dX}{dt} &= \alpha \, m_X - \lambda \, X \end{aligned} \tag{12}$$

where mRNA is transcribed at a rate ω and degraded at a rate δ_m and proteins are translated at a rate α and diluted through cell growth. At steady-state, the synthetic proteome fraction is given by

$$\Phi_S = \Phi_X = \frac{n_X}{\rho_{cell}} \, \overline{X} = \frac{n_X \, \omega \, \alpha}{\rho_{cell} \, \delta_m} \, \frac{1}{\lambda} \tag{13}$$

which clearly does not meet the requirements stated above (Supplementary Figure 20a), since it scales as $1/\lambda$.

Our goal is to find a simple expression that captures how the required links between growth rate and constitutive gene expression listed above, may arise from coupling the circuit to the gene-expression machinery of the cell. As a first step, we introduce a dependency of the circuit on the cellular translation machinery, which is known to be the most important resource bottleneck(11) in *E. coli*. For simplicity, we assume mass action kinetics, with R representing a unit of *active* translational resources[1] (active ribosome plus associated factors required for protein production). The

[1]For a possible derivation of this mass-action term from a model of peptide elongation, see(12)

ODEs become[2]

$$\begin{aligned}\frac{dm_X}{dt} &= \omega - \delta_m\, m_X \\ \frac{dX}{dt} &= \alpha\, m_X\, R - \lambda\, X\end{aligned} \tag{14}$$

We can convert the concentration of active translational machinery to its proteome mass fraction $(\Phi_R - \Phi_{R_0})$ and then use the first growth law (eq. 3) to express it as a function of the growth rate

$$R = \frac{\rho_{cell}}{n_R}(\Phi - \Phi_{R_0}) = \frac{\rho_{cell}}{n_R\,\gamma_0}\,\lambda \tag{15}$$

With this, the ODE for the protein concentration becomes

$$\frac{dX}{dt} = \alpha\,\frac{\rho_{cell}}{n_R\,\gamma_0}\,m_X\,\lambda\, - \lambda\,X \tag{16}$$

which would imply that at steady-state the concentration (and mass fraction) of the constitutive protein is independent of the growth rate (Supplementary Figure 20b),

$$\Phi_S = \frac{n_X}{\rho_{cell}}\,\overline{X} = \frac{n_X\,\omega\,\alpha}{n_R\,\gamma_0\,\delta_m} \tag{17}$$

again not capturing the expected behavior of a constitutive gene.

In order to determine what the correct expression would look like, we take a more careful look at equation 13. The synthetic proteome fraction scales inversely with growth rate, because the dilution term is set by the growth rate. From equation 13, then we can infer that the production term in the ODE describing the protein concentration must scale as $\frac{\lambda^2}{\nu}$, if the synthetic proteome fraction is to be proportional to the P-type fraction

$$\Phi_S \propto \Phi_P = \frac{\lambda}{\nu} \tag{18}$$

We interpret this result in the following way. The squared dependency on growth rate arises quite naturally if we consider that the availability of transcriptional machinery also depends on the cellular growth rate. Here, we refer to a recent study by Balakrishnan *et al.*, in which the authors monitor transcriptome- and proteome-wide changes under different growth conditions, in order to investigate at a very detailed level how the observed growth-rate dependencies arise naturally from the central dogma of molecular biology(13).

Balakrishnan *et al.* report that the mRNA and protein levels correlate linearly for the majority of genes in *E. coli*. Moreover, after carefully quantifying the contributions of each step of gene expression, they conclude that the concentrations of proteins are dominantly set by the transcriptional output of their genes and not by other factors, such as translation initiation rates, mRNA degradation rates or gene dosage in replicating chromosomes. Balakrishnan *et al.* further estimate that the concentration of active RNA polymerase is tightly coupled to the concentration of active ribosomes. Therefore, it exhibits the same dependencies on growth rate as the ribosomal proteome fraction, i.e. the growth laws described in the previous section. Since the actual concentration of RNA polymerase components seems to remain constant under different growth conditions, the authors present evidence for a mechanism by which the availability of *active* RNA polymerase is modulated by the anti-sigma factor Rsd, which indeed exhibits the

[2] To keep notation simple, we have left α as a symbol for the translation rate, although it must be noted that it now has different units compared to the quantity in eq. 12. The same will be true below, when we consider the role of transcriptional resources and the dependency on the media quality.

expected growth-rate dependencies.

Based on the findings of Balakrishnan *et al.*, we now consider that the transcription rate of the constitutive gene reflects the overall availability of RNA polymerase, as is the case for the translation rate and the active ribosomes

$$\frac{dm_X}{dt} = \omega\,\mathrm{RNAP} - \delta_m\, m_X \tag{19}$$

Since Balakrishnan *et al.* determined that the concentration of active RNA polymerase correlates positively with growth rate, we set

$$\mathrm{RNAP} = \beta\,\lambda \tag{20}$$

so that the ODE describing the mRNA concentration becomes

$$\frac{dm_X}{dt} = \widetilde{\omega}\,\lambda - \delta_m\, m_X \tag{21}$$

where we have absorbed the proportionality factor into the transcription rate.

With this, the equation for the synthetic proteome fraction becomes

$$\Phi_S = \frac{n_X}{\rho_{cell}}\,\overline{X} = \frac{n_X\,\widetilde{\omega}\,\alpha}{n_R\,\gamma_0\,\delta_m}\,\lambda \tag{22}$$

This expression looks already more promising, since it is proportional to the growth rate. However, it does not distinguish between cases in which the growth rate is varied through changes in nutritional capacity or translational capacity (Supplementary Figure 20c). Comparison to eq. 18 reveals that in order to achieve a behavior that does distinguish these two cases, there is still a factor $\frac{1}{\nu}$ missing.

It is less straightforward to derive the presence of this factor from first principles. However, we note that Scott *et al.* propose that the observed difference between the behavior of the P-sector and the R-sector reflects the balance between catabolic and anabolic fluxes in *E. coli*(5, 6). The factor $\frac{1}{\nu}$ captures this overall regulatory strategy, which affects a large portion of genes in parallel.

Since Balakrishnan *et al.* provide evidence that the concentrations of proteins are set foremost by the transcriptional activity, we here assume that the factor $\frac{1}{\nu}$ enters at that stage of the gene expression process. The final model for the expression of a constitutive gene thus becomes

$$\begin{aligned} \frac{dm_X}{dt} &= \widetilde{\omega}\,\frac{\lambda}{\nu} - \delta_m\, m_X \\ \frac{dX}{dt} &= \widetilde{\alpha}\, m_X\,\lambda - \lambda\, X \end{aligned} \tag{23}$$

where we have absorbed the constants $\frac{\rho_{cell}}{n_R\,\gamma_0}$ into an effective translation rate $\widetilde{\alpha}$.

These final equations have the same number of free parameters than the conventional model of the constitutive gene we started from. The nutritional capacity ν is a property of the media in which the cells are grown and it can be easily determined by measuring the growth rate of the strain in the absence of the circuit and then solving for ν in eq.10. We note that the choice of adding the factor $\frac{1}{\nu}$ to the transcriptional step is arbitrary. It could also be added to the translation step and still the predictions at the protein level would be consistent with the bacterial growth laws. Moreover, if the circuit of interest operates in a context where the media is fixed and there are no changes in nutrient quality, it becomes unnecessary to consider the factor $\frac{1}{\nu}$, since it can be absorbed into the production constants $\widetilde{\omega}$ or

$\widetilde{\alpha}$.

Here, we have simply added an explicit growth-rate dependency to the production rates of mRNA and protein to reflect the fact that these depend on the physiological state of the cell through their reliance on the host's gene expression machinery. As Supplementary Figure 20d shows, our modified equations are able to capture the expected qualitative behavior of a constitutive gene, both in the case when media quality is modulated and when the translational machinery is inhibited by a bacteriostatic antibiotic. Since the general dependence on the host gene-expression machinery does not only apply to constitutive genes, but to any heterologous gene that forms part of a synthetic network of genes[3], in the following selection we generalize the framework derived here to an arbitrary synthetic genetic circuit.

General Formulation of the framework

The host-aware framework we propose here can be applied to any arbitrary synthetic genetic circuit. It consist of simple modifications to a conventional ODE model, which incorporate a two-way coupling between the circuit and the host. The circuit impacts the host by sequestering cellular resources, which causes a reduction in the growth rate. The host influences the performance of the circuit, because the latter depends on the availability of cellular gene-expression machinery and this is tightly regulated in accordance to the host's physiological state.

Consider a generic synthetic genetic circuit composed of u genes that are introduced into *E. coli* to produce proteins $X_1, \cdots, X_u$. A conventional model of such a system will consist of $2u$ ordinary differential equations, describing the mRNA and protein concentrations of the circuit's components. To these ODEs, we add the factors derived in the previous section to the production rates of mRNA and protein, so that they take the form

$$\begin{aligned} \frac{dm_{X_i}}{dt} &= \widetilde{\omega}_i \, T_i(\mathbf{m_X}, \mathbf{X}) \, \frac{\lambda}{\nu} + F_i(\mathbf{m_X}, \mathbf{X}) - \delta_i \, m_{X_i} \\ \frac{dX_i}{dt} &= \widetilde{\alpha}_i \, m_{X_i} \, \lambda + G_i(\mathbf{m_X}, \mathbf{X}) - \lambda \, X_i \end{aligned} \tag{24}$$

with $i = 1, \cdots u$. The collections of functions $\mathbf{F}(\mathbf{m_X}, \mathbf{X})$ and $\mathbf{G}(\mathbf{m_X}, \mathbf{X})$ describe potential interactions between the components of the circuits at the mRNA and protein level, respectively, and the functions $\mathbf{T}(\mathbf{m_X}, \mathbf{X})$ reflect potential regulatory input at the transcriptional level.

The explicit dependency of the production and dilution rates on the growth rate reflects the impact of resource availability and host growth on the concentration of circuit components. To get an explicit expression for the growth rate, which reflects the impact of the circuit on the host, we make use of the expressions derived by Scott *et al.*[4] for the case of heterologous gene expression (eq. 10).

We can use the conversion factor derived above to calculate the proteome fraction occupied by the proteins in the circuit at any particular point in time:

$$\Phi_S = \frac{\sum_{i=1}^{u} n_{X_i} \, X_i}{\rho_{cell}} \tag{25}$$

This can in turn be inserted into equation 10 to obtain an explicit expression for the instantaneous growth rate

$$\lambda \left(\Phi_S \right) = \left(\Phi_R^{\max} - \Phi_{R_0} - \Phi_S \right) \, \frac{\gamma_0 \, \nu}{\gamma_0 + \nu} \tag{26}$$

[3]This excludes genes whose expression does not depend on cellular factors, such as genes transcribed by exogenous polymerases or translated by orthogonal ribosomes(14).

[4]This assumes that the dynamics of the synthetic circuit can be taken to be in quasi-equilibrium compared to the dynamics of the host's physiology. We simply assume this here, since this results in model predictions that match the experimental data for our circuit of interest.

Equations 24, 25 and 26 form a closed system of $2u$ ODEs and 2 algebraic equations that can be simulated to approximate the dynamic behavior of the synthetic genetic circuit in the context of an exponentially growing *E. coli* host.

Model parameters

Under the growth conditions investigated by Scott *et al.*(5), the estimated values for $\Phi_R^{\max}$, Φ_{R_0} and γ_0 were largely constant. As long as the cells under consideration are grown under similar conditions[5], which will be true of most cases in a laboratory environment, the values they determined for these three parameters can be used.

Apart from these, the number of amino acids that compose each protein, n_{X_i} is known, as well as the cellular protein density ρ_{cell}, which is largely growth-rate independent. The nutritional capacity ν, which describes the quality of the media, can be easily determined by measuring the growth rate of the strain in the absence of the synthetic genetic circuit ($\Phi_S = 0$) and substituting in equation 26:

$$\nu = \frac{\gamma_0\,\lambda}{\gamma_0\,(\Phi_R^{\max} - \Phi_{R_0}) - \lambda} \tag{27}$$

In that case, the framework we propose contains the same amount of free parameters as a conventional model that does not consider the host, that is u transcription rates, u mRNA degradation rates, u translation rates plus any number of parameters required to describe the interactions between circuit species. This framework then has the advantage that it can be easily incorporated into an existing ODE model and that it can capture the qualitative trends expected from a host-aware model without adding complexity or increasing the risk of overfitting. The parameter values we use in this study are summarized in Supplementary Table 1.

Model of photophilic strain

In this section, we apply the framework formulated above to the case of the growth-control circuit in the photophilic strain. The circuit consists of the two parts of a split opto-T7, which dimerizes in the presence of blue light to produce an active complex that transcribes mRNA coding for the resistance enzyme chloramphenicol acetyltransferase (CAT).

We begin by presenting the final result and then derive the equations in the following sections. Our model is composed of three ODEs and two algebraic equations:

$$\frac{dT_T}{dt} = \hat{\alpha}_T\,\frac{\lambda^2}{\nu} - \lambda\,T_T \tag{28}$$

$$\frac{dT_D}{dt} = h_{ON}(L)\,\left(T_T - 2\,\left(T_D + g_{ON}(T_D)\right)\right)^2 - \lambda\,\left(T_D + g_{ON}(T_D)\right) \tag{29}$$

$$\frac{dC}{dt} = \hat{\alpha}_C\,\frac{\lambda}{\nu}\,g_{ON}(T_D) - \lambda\,C \tag{30}$$

$$\Phi_S(t) = \frac{n_T\,T_T(t) + n_C\,C(t)}{\rho_{cell}} \tag{31}$$

$$\lambda(t) = \left(\Phi_R^{max} - \Phi_{R_0} - \Phi_S(t)\right)\,\frac{\nu\,\gamma_0}{\gamma_0 + \nu\,\left(1 + \frac{\frac{A_E}{K_D}}{1+\left(\frac{C(t)}{\kappa\,\hat{K}_C}\right)^{h_C}}\right)} \tag{32}$$

The ODEs describe the evolution of the concentration of the circuit's proteins: T7-monomers, T_T, T7-dimers, T_D and CAT, C. For simplicity, we have assumed fast mRNA dynamics for all species (quasi-steady state), which in our

[5]Carbon-limited exponential growth at 37°C with saturating amounts of carbon source, in the presence or absence of translation-inhibiting antibiotics.

framework corresponds to:

$$m_{X_i}^{qss} = \frac{\widetilde{\omega}_i}{\delta_i} \frac{\lambda}{\nu} \tag{33}$$

and we have absorbed the transcription and mRNA-degradation rates into an effective protein-production rate $\widetilde{\alpha}_i$.

The light-dependent T7-dimerization rate is modeled by the general expression

$$h_{ON}(L) = h_{ON}^{min} + \left(h_{ON}^{max} - h_{ON}^{min}\right) \frac{L^{n_L}}{K_L^{n_L} + L^{n_L}} \tag{34}$$

where L is the blue-light intensity, and the factor

$$g_{ON}(T_D) = N_p \frac{T_D^{n_G}}{K_G^{n_G} + T_D^{n_G}} \tag{35}$$

captures the concentration of actively transcribing T7-dimers.

The difference between equation 32 and equation 26 reflects the effect of chloramphenicol inhibition.

The equations used here are mainly phenomenological in nature. Nevertheless, their utility is supported by the good results obtained, both in recapitulating the gene expression and growth rate dynamics of the photophilic strain, as well as in predicting the trajectories of the closed-loop co-culture. In the following sections, we provide the rationale behind the choice of terms for our model.

Production of CAT

To get a simple description of transcription by T7 polymerase, we treat it as a two step process

$$T_D + g_{OFF} \underset{\beta_-}{\overset{\beta_+}{\rightleftharpoons}} g_{ON} \xrightarrow{\widetilde{\omega}_C} T_D + g_{OFF} + m_C$$

First, a T7-dimer first binds reversibly to a copy of the gene which is in an inactive state, g_{OFF}, to produce an active complex, g_{ON}. The complex then directly produces a unit of CAT mRNA, m_C, thereby releasing the T7-dimer and the gene, which reverts back to its OFF state. Finally, the CAT gene resides on a plasmid, whose copy number, $N_P = g_{OFF} + g_{ON}$, we assume is kept constant through a balance between plasmid replication and dilution:

$$\varnothing \xrightarrow{\lambda N_p} g_{OFF} \xrightarrow{\lambda} \varnothing$$
$$g_{ON} \xrightarrow{\lambda} \varnothing$$

These reactions lead to the following ODEs for the time evolution of the inactive and active gene copies

$$\begin{aligned} \frac{dg_{OFF}}{dt} &= \lambda N_p - \beta_+ g_{OFF} T_D + (\beta_- + \widetilde{\omega}_C) g_{ON} - \lambda g_{OFF} \\ \frac{dg_{ON}}{dt} &= \beta_+ g_{OFF} T_D - (\beta_- + \widetilde{\omega}_C) g_{ON} - \lambda g_{ON} \end{aligned} \tag{36}$$

Assuming that these dynamics evolve at a faster timescale than that of processes related to the changes in cell growth and physiology, we consider steady-state for these equations, which leads to the following expression for the concentration of active gene copies

$$g_{ON} = N_p \, \frac{T_D}{T_D + \frac{\beta_- + \tilde{\omega}_C}{\beta_+} - \frac{\lambda}{\beta_+}} \approx N_p \, \frac{T_D}{T_D + K_G} \tag{37}$$

where we have used the fact that the binding reaction of the T7 to its promoter is orders of magnitude faster than cell growth and we have introduced $K_G = \frac{\beta_- + \tilde{\omega}_C}{\beta_+}$ as a parameter that relates to the affinity of the T7 to its promoter sequence. We finally add a hill-coefficient to this expression to be able to capture higher degrees of non-linearity in the system, leading to the expression in equation 35.

With this, and assuming the mRNA concentration is in quasi-steady state (eq. 33), we can write the ODE for the concentration of CAT as

$$\frac{dC}{dt} = \hat{\alpha}_C \, \frac{\lambda}{\nu} \, N_p \, \frac{T_D^{n_G}}{T_D^{n_G} + K_G^{n_G}} - \lambda \, C \tag{38}$$

where we have again absorbed the parameters related to transcription into the effective production rate $\hat{\alpha}_C$. Note that, in this equation, the protein production rate scales only linearly with the growth rate. This is because, in the derivation of our framework, the square dependency arises from the fact that both the ribosomes and RNA polymerase introduce a linear dependency on the growth rate. However, since CAT is not transcribed by the endogenous *E. coli* polymerase, we let it scale linearly with the growth rate to reflect the fact that it only depends on the cellular translation machinery.

Light-dependent dimerization

We begin by representing light-mediated dimerization of the split T7 through the following reversible reaction

$$T_M + T_M \underset{h_{OFF}(L)}{\overset{h_{ON}(L)}{\rightleftharpoons}} T_D$$

In reality, the opto-T7 is a heterodimer, composed of two different N-terminal, nMag-T7$_{\text{N-Ter}}$, and C-terminal, pMag-T7$_{\text{C-Ter}}$, parts. However, for simplicity we assume that we can model the opto-T7 as a homodimer that results from the light-promoted fusion of two identical monomer units, T_M.

We chose a general Hill-function, each with 4 free parameters, for the forward and backward rates of the dimerization reaction, in order to capture the dynamics of this process accurately

$$\begin{aligned} h_{ON}(L) &= h_{ON}^{min} + \left(h_{ON}^{max} - h_{ON}^{min}\right) \, \frac{L^{n_{L_{ON}}}}{K_L^{n_{L_{ON}}} + L^{n_{L_{ON}}}} \\ h_{OFF}(L) &= h_{OFF}^{max} - \left(h_{OFF}^{max} - h_{OFF}^{min}\right) \, \frac{L^{n_{L_{OFF}}}}{K_L^{n_{L_{OFF}}} + L^{n_{L_{OFF}}}} \end{aligned} \tag{39}$$

The ODE for the T7 dimers includes the dimerization and transcription reactions

$$\frac{dT_D}{dt} = h_{ON}(L) \, T_M^2 - h_{OFF}(L) \, T_D - \beta_+ \, g_{OFF} \, T_D + (\beta_- + \tilde{\omega}_C) \, g_{ON} - \lambda \, T_D \tag{40}$$

$$= h_{ON}(L) \, T_M^2 - h_{OFF}(L) \, T_D - \lambda \, g_{ON} - \lambda \, T_D \tag{41}$$

However, simulations revealed that the dynamics of T_D were only weakly influenced by the dissociation reaction, in contrast to the forward reaction, which scales with the square of the monomer concentration. Because of this

and in order to reduce the number of free parameters, we neglected the dissociation reaction, effectively considering dimerization as an irreversible process. This leads to the simpler equation

$$\frac{dT_D}{dt} = h_{ON}(L)\, T_M^2 - \lambda\,(g_{ON} + T_D) \tag{42}$$

Finally, we chose to keep track of the concentration of *total* T7-monomers, T_T, instead of that of *free* T7-monomers, T_M. T_T is given by

$$T_T = T_M + 2\,(T_D + g_{ON}) \tag{43}$$

and its concentration is not affected by dimerization or transcription reactions, so that its dynamics follow the simple ODE

$$\frac{dT_T}{dt} = \hat{\alpha}_T\,\frac{\lambda^2}{\nu} - \lambda\, T_T \tag{44}$$

which is equation 28. Using equation 43 to remove T_M from the ODE for T_D, we recover equation 29.

Inhibition of growth by chloramphenicol

What remains to be discussed is how to model the inhibitory effect of chloramphenicol on growth and how this is alleviated in the presence of the resistance. For this, we follow closely the approach taken by Deris *et al.*(8) and we refer to their paper for further details.

Chloramphenicol inhibits translational elongation by direct binding to the 50S subunit of the ribosome. We model this by considering that the ribosomes can be in one of three states. R_u are active, chloramphenicol-unbound ribosomes which can be engaged in translation. R_b are ribosomes that have been inhibited by antibiotic binding and R_0 are the naturally-occurring inactive ribosomes that give rise to the Y-offset in the bacterial growth law, eq. 3. We model chloramphenicol inhibition as a reversible binding reaction

$$R_u + A_I \underset{k_-}{\overset{k_+}{\rightleftharpoons}} R_b \xrightarrow{\lambda} \varnothing$$

where A_I is the intracellular chloramphenicol concentration. This leads to the following ODE for the concentration of the bound ribosomal species

$$\frac{dR_b}{dt} = k_+\, R_u\, A_I - k_-\, R_b - \lambda\, R_b \tag{45}$$

Reversible chloramphenicol binding happens at a faster timescale than growth and gene expression. Therefore, we assume R_b to be in quasi-steady state, from which we get

$$R_u = \frac{\left(1 + \frac{\lambda}{k_-}\right)\,(R - R_0)}{1 + \frac{k_+}{k_-}\, A_I + \frac{\lambda}{k_-}} \approx \frac{1}{1 + \frac{A_I}{K_D}}\,(R - R_0) \tag{46}$$

where we have used the fact that $k_- \gg \lambda$ and we have introduced the total ribosomal concentration $R = R_u + R_b + R_0$ and the parameter $K_D = \frac{k_-}{k_+}$, which relates to the affinity of chloramphenicol to the ribosome. Equation 46 reveals that the presence of chloramphenicol reduces the concentration of actively translating ribosomes by a factor $\frac{1}{1+\frac{A_I}{K_D}}$.

Since we can transform ribosomal concentrations to proteome mass fractions by multiplication with the factor $\frac{n_R}{\rho_{cell}}$, the same applies to the corresponding mass fractions

$$\Phi_{R_u} = \frac{1}{1 + \frac{A_I}{K_D}} \left(\Phi_R - \Phi_{R_0}\right) \tag{47}$$

In the presence of chloramphenicol, only unbound ribosomes contribute to biomass synthesis, so the first bacterial growth law becomes

$$\lambda = \gamma_0 \, \Phi_{R_u} = \frac{\gamma_0}{1 + \frac{A_I}{K_D}} \left(\Phi_R - \Phi_{R_0}\right) \tag{48}$$

Again, we combine this equation with the second growth law

$$\Phi_P = \frac{\lambda}{\nu}$$

and the finite size of the proteome

$$\Phi_Q + \Phi_R + \Phi_P + \Phi_S = 1$$

to obtain an updated equation for the instantaneous growth rate in the presence of chloramphenicol

$$\lambda = \left(\Phi_R^{max} - \Phi_{R_0} - \Phi_S(t)\right) \frac{\nu \, \gamma_0}{\gamma_0 + \nu \left(1 + \frac{A_I}{K_D}\right)} \tag{49}$$

Finally, we turn to how the intracellular concentration of chloramphenicol, A_I, can be related to the extracellular concentration, A_E, which is the one we actually set in experiments. Apart from the reversible binding to the ribosomes, we assume that chloramphenicol enters the cell passively via diffusion and is also degraded catalytically by the action of CAT

$$\varnothing \underset{\kappa}{\overset{\kappa \, A_E}{\rightleftharpoons}} A_I$$
$$A_I + C \underset{v_-}{\overset{v_+}{\rightleftharpoons}} A^* \xrightarrow{v_f} C$$

The ODEs that describe the collection of reactions involving chloramphenicol are the following

$$\begin{aligned} \frac{dA_I}{dt} &= \kappa(A_E - A_I) - v_+ \, A_I \, C + v_- \, A^* - k_+ \, A_I \, R_u + k_- \, R_b - \lambda \, A_I \\ \frac{dA^*}{dt} &= v_+ \, A_I \, C - v_- \, A^* - v_f \, A^* - \lambda \, A^* \end{aligned} \tag{50}$$

Assuming that degradation is a fast process, we assume quasi-steady state for $A*$, which leads to

$$A^* = \frac{\frac{v_+}{v_- + v_f}}{1 + \frac{\lambda}{v_- + v_f}} \, A_I \, C \approx \frac{A_I \, C}{K_C} \tag{51}$$

where we have again used the fact that binding and degradation reactions are fast compared to cell growth, $v_- + v_f \gg \lambda$, and we have introduced the parameter $K_C = \frac{v_- + v_f}{v_+}$, which relates to the affinity of the degradation reaction.

Using this result, together with the quasi-steady state concentration of bound ribosomes from eq. 45, we obtain

$$
\begin{aligned}
\frac{dA_I}{dt} &= \kappa(A_E - A_I) - \frac{1+\frac{\lambda}{v_f}}{\frac{K_C}{v_f}} A_I\, C - \lambda \frac{A_I\, R_u}{K_D} - \lambda\, A_I \\
&\approx \kappa\, A_E - A_I \left[\kappa + \frac{C}{\hat{K}_C} + \frac{\rho_{cell}}{n_r\, \gamma_0\, K_D} \lambda^2 + \lambda\right]
\end{aligned}
\tag{52}
$$

where we use the first growth law to replace R_u with $\frac{\rho_{cell}\,\lambda}{n_r\,\gamma_0}$, we absorb v_f into $\hat{K}_C = \frac{v_- + v_f}{v_+\, v_f}$ and for the last approximation we use the fact that $v_f \gg \lambda$.

We now examine the value ranges each term in this equation can take, to determine the ones that dominate. For the sources from which we obtain our parameter values, see Supplementary Table 1.

- For the concentrations of chloramphenicol and light intensities used in our experiments, we obtain growth rate values in the range $\lambda \in [0.015,\, 0.033]\frac{1}{min}$
- With this, the term with square-dependency on the growth rates takes on values in the interval $[0.1382,\, 3]\frac{1}{min}$
- The diffusion rate $\kappa \approx 90\frac{1}{min}$ is one to two orders of magnitude larger than the last two terms
- Since $\hat{K}_C \approx 0.333\, nM\, min$, the term $\frac{C}{\hat{K}_C}$ scales as $3\,C$, which can become comparable to κ or dominant for even low concentrations of CAT.

Because of this, we further approximate the ODE for A_I as

$$
\frac{dA_I}{dt} \approx \kappa\, A_E - \kappa\, A_I + \frac{C}{\hat{K}_C} A_I \tag{53}
$$

which, since A_I only takes part in fast binding or degradation reactions can be taken to be at quasi-steady state, leading to

$$
A_I \approx \frac{A_E}{1 + \frac{C}{\kappa\, \hat{K}_C}} \tag{54}
$$

Inserting equation 54 back into equation 49, we recover the expression for growth rate in our model, eq. 32, except for the exponent h_C, which we added *a posteriori* because it improved the capability of the model to capture the non-linear response of the photophilic strain in experiments.

Modeling the dynamics of co-culture composition

We consider an exponentially growing two-strain co-culture in a turbidostat setup. The accumulation of total biomass M_T in the vial is given by the combined exponential growth of the two strains that are present (in this section, p and c subscripts denote the photophilic and constitutive strains respectively):

$$
\frac{dM_T}{dt} = \lambda_p\, M_p + \lambda_c\, M_c
$$

The principle of a turbidostat consists in balancing the biomass increase rate by an equal dilution rate, so that the overall density of the culture is kept constant. To get a formula for this effective rate of dilution, d, we set

$$
\frac{dM_T}{dt} \stackrel{!}{=} d\, M_T
$$

which leads to

$$d = \frac{\lambda_p \, M_p + \lambda_c \, M_c}{M_p + M_c} = \lambda_p \, \varphi_p + \lambda_c \, \varphi_c$$

where we have defined the strain fractions in the co-culture $\varphi_p = \frac{M_p}{M_p + M_c}$ and $\varphi_c = \frac{M_c}{M_p + M_c}$, with $\varphi_c + \varphi_p = 1$.

The differential equation governing the dynamics of the photophilic fraction, φ_p can be derived as follows:

$$\frac{d\varphi_p}{dt} = \frac{d}{dt} \frac{M_p}{M_T} = \frac{1}{M_T} \frac{dM_p}{dt} + M_p \frac{d}{dt} \frac{1}{M_T} = \frac{1}{M_T} \frac{dM_p}{dt} = \frac{\lambda_p \, M_p - d \, M_p}{M_T} = (\lambda_p - d) \, \varphi_p$$

where we have used the fact that in turbidostat mode $\frac{dM_T}{dt} = 0$. Inserting the expression for the dilution rate d from above and noting that $\varphi_c = 1 - \varphi_p$ we get the following ODE for φ_p:

$$\frac{d\varphi_p}{dt} = (\lambda_p - \lambda_c) \, (1 - \varphi_p) \, \varphi_p \tag{55}$$

The equation provides immediate intuition about the co-culture dynamics, with three qualitatively different scenarios which are schematically shown in Figure 5a of the main text. Dynamic equilibria arise when the derivative in equation 55 vanishes. This happens for $\varphi_p = 1$ and $\varphi_p = 0$, corresponding to the extinction of either the constitutive or the photophilic strain.

The stability of the fixed points is determined by the sign of $\frac{d\varphi_p}{dt}$ close to the fixed point. Since $0 \leq \varphi_p$ and $0 \leq 1 - \varphi_p$, the sign of the derivative is completely determined by the sign of the first factor. If $\lambda_c > \lambda_p$, the fixed point at $\varphi_p = 1$ becomes unstable and the co-culture evolves towards $\varphi_p = 0$, a culture dominated by the constitutive strain. Alternatively, if $\lambda_p > \lambda_c$, the fixed point at $\varphi_p = 0$ becomes unstable and the photophilic strain will inevitably come to dominate. In both of these cases, the magnitude of the difference in growth rates sets how fast the system converges to the stable equilibrium.

When both strains grow at the same pace ($\lambda_c = \lambda_p$), the derivative vanishes irrespective of the value of φ_p. This means that the co-culture will maintain any particular ratio that it has when λ_c and λ_p first become equal.

In view of this, the control strategy for stabilizing the strain ratio with optogenetic feedback becomes clear. If the ratio is lower than the desired value, light levels that result in the photophilic strain growing faster than the constitutive one should be applied, steering the system towards $\varphi = 1$. The opposite is true if the ratio is higher than the desired value. Then, once the desired setpoint is reached, the light level should be such as to precisely ensure that the growth rates are equal, so that the desired ratio is maintained over time.

The analysis also reveals the fragility of the co-existence equilibrium. Even small fluctuations in the growth rate will cause the system to evolve towards a monoculture.

For our simulations, λ_c is assumed to be constant. On the contrary, λ_p is calculated at any given time by solving the set of equations 29-32 for the current external light input L, in order to compute the instantaneous growth rate of the photophilic strain via equation 32.

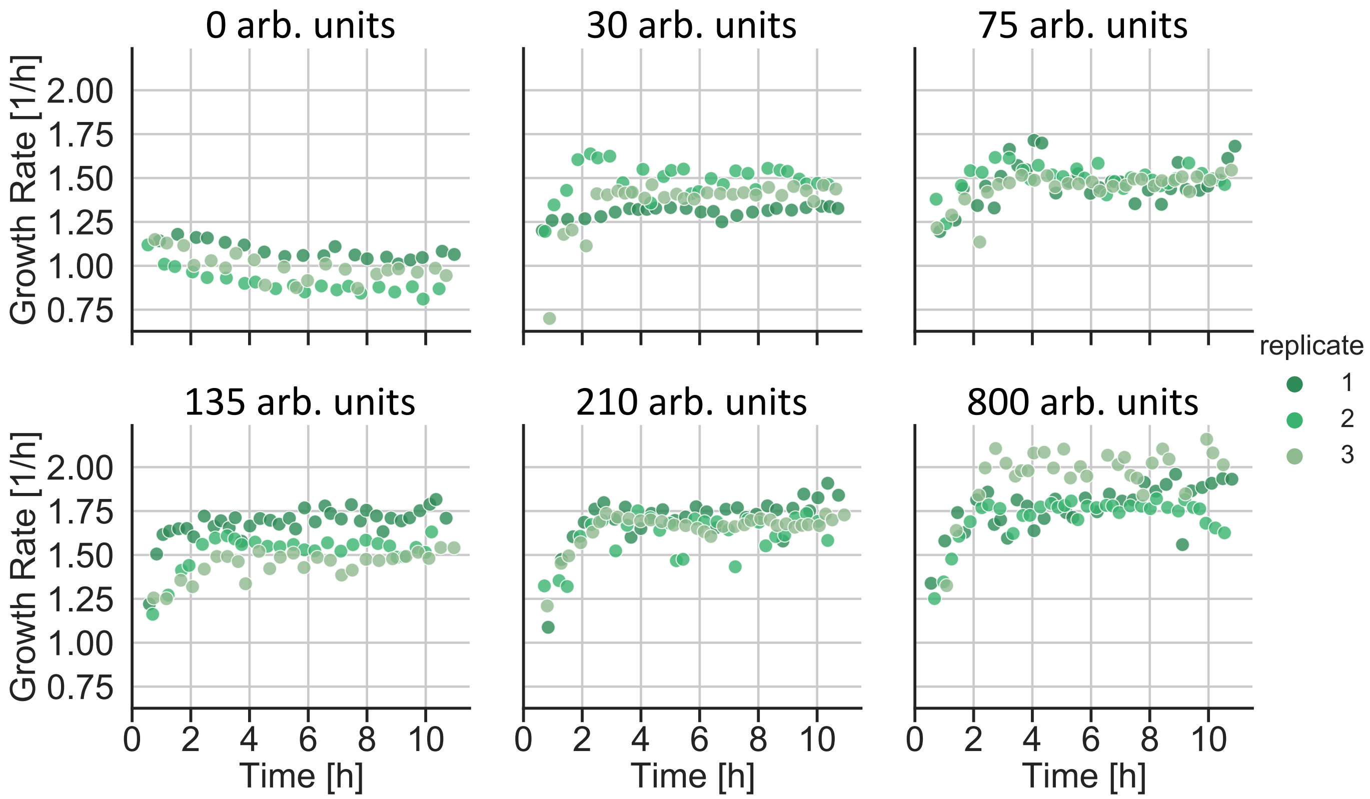

Supplementary Fig. 1. Dose-response of photophilic strain.
This figure depicts the raw data for determination of the steady-state growth rate of the photophilic strain in response to light (Figure 2b). For different light intensities, each run corresponds to a biological replicate carried out on a different day. The steady-state growth rates depicted in Figure 2b correspond to the median of each replicate over time in the time interval 6h¡t¡10h. Source data are provided as a Source Data file.

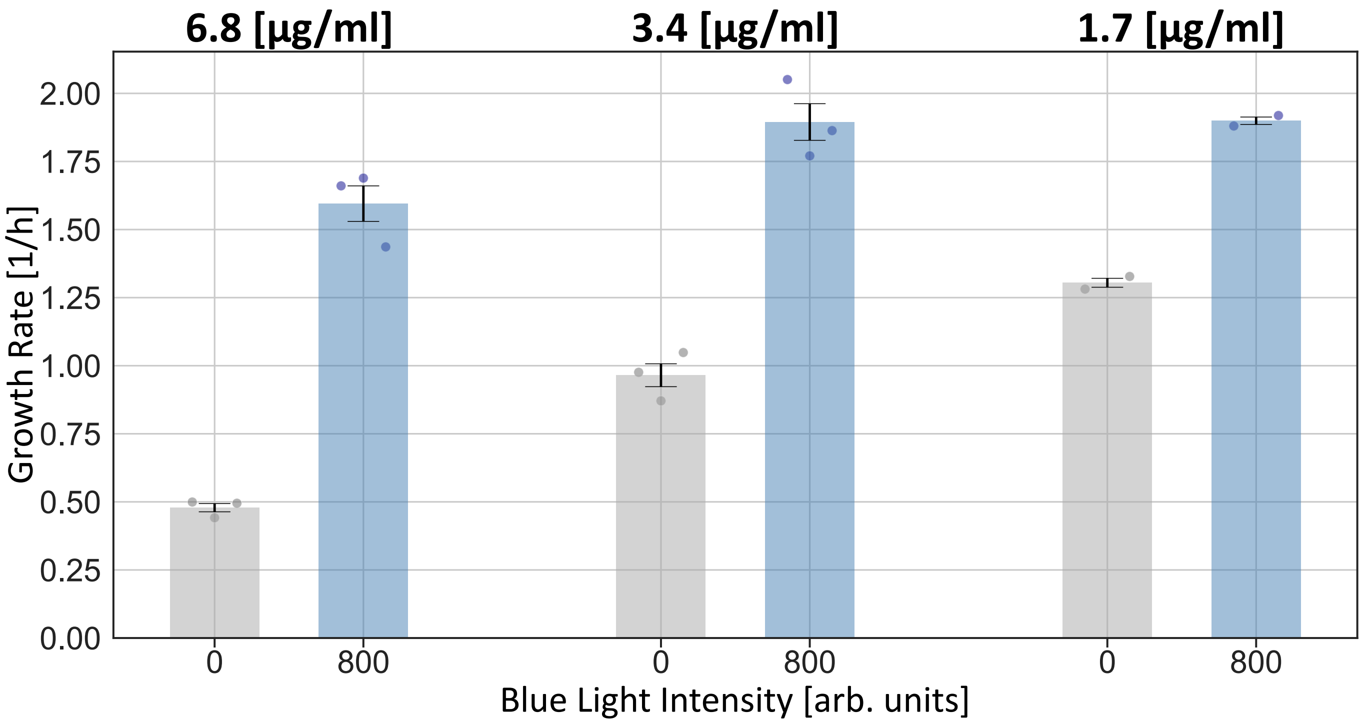

Supplementary Fig. 2. Growth of photophilic strain with different external concentrations of chloramphenicol The growth rate of the photophilic strain can be modulated by changing the light intensity or the external concentration of chloramphenicol. Different bars correspond to equilibrium growth rate of the photophilic strain in the presence of different chloramphenicol concentrations and either no light or the maximal intensity used in this study (Data are presented as mean values +/- SEM, n=3 biologically independent samples for 6.8 and 3.4 [ug/ml] and n=2 biologically independent samples for 1.7 [ug/ml]). For the rest of the study, we used 3.4 ug/ml as fixed chloramphenicol concentration, because it leads to a good fold-change in growth, while maintaining a saturated growth rate when the resistance is fully induced. We hypothesized that this would reduce the selection pressure during closed-loop co-culture experiments for mutations that escape the growth control circuit. Source data are provided as a Source Data file.

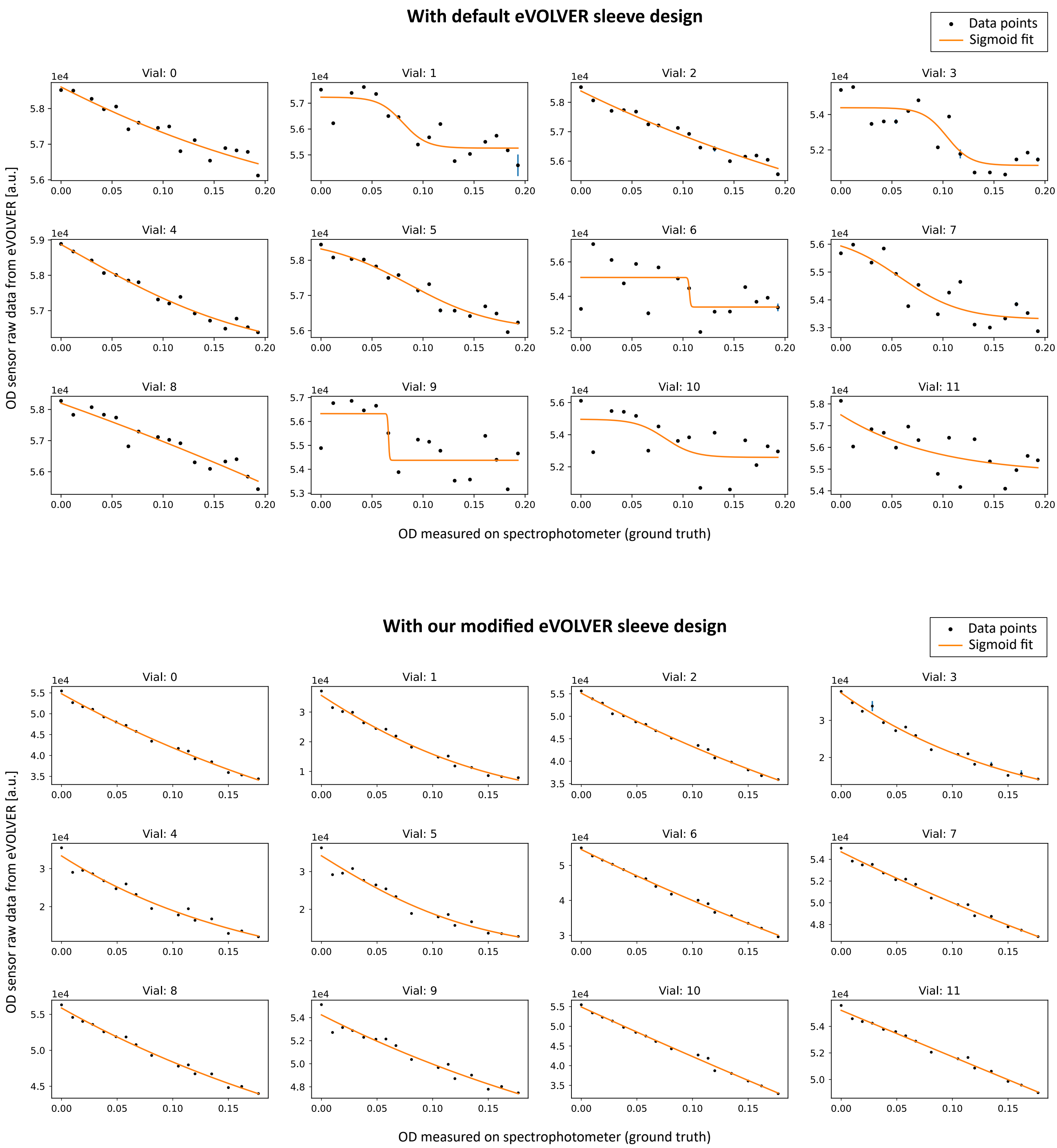

Supplementary Fig. 3. Improved OD measurement with modified eVOLVER sleeve design
As shown in Figure 3a **(Left)**, we re-designed the glass vial cap and the tube-holder with added O-rings to prevent wobbling of the culture vial inside the eVOLVER sleeve. This resulted in very stable and consistent OD sensor measurements compared to that with the default eVOLVER sleeve design. These plots show the results of the calibration procedure of the OD-sensors in different eVOLVER sleeves, performed with the same calibration standards on the same day. The OD calibration was carried out once and the saved calibration file used for all experiments in this study. The plots were generated automatically by the eVOLVER and source data can be provided upon reasonable request.

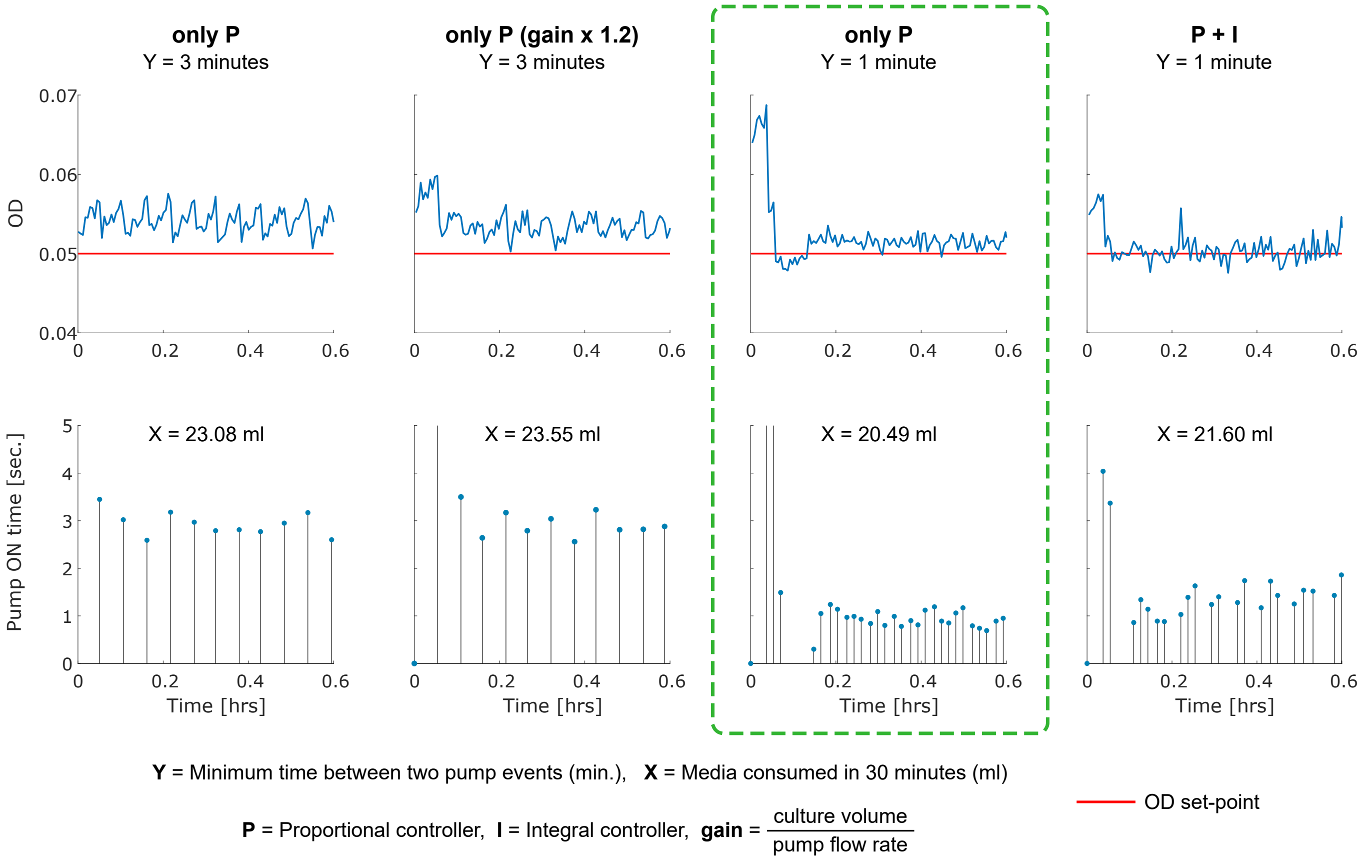

Supplementary Fig. 4. OD regulation controller parameter tuning.
We used the modified eVOLVER platform in turbidostat mode in order to maintain the cell culture density in a desired range in our experiments. This mode has an OD regulation feedback controller continuously running in the background. Based on OD measurements, this controller determines the duration for which the media pump should be switched ON in order to dilute the culture to a desired density. We performed multiple trial experiments with different OD regulation controller parameters. In all of our experiments in this study, we chose the controller parameters which exhibited better OD set-point tracking with less media consumption (highlighted with green dashed line box in this figure). Source data are provided as a Source Data file.

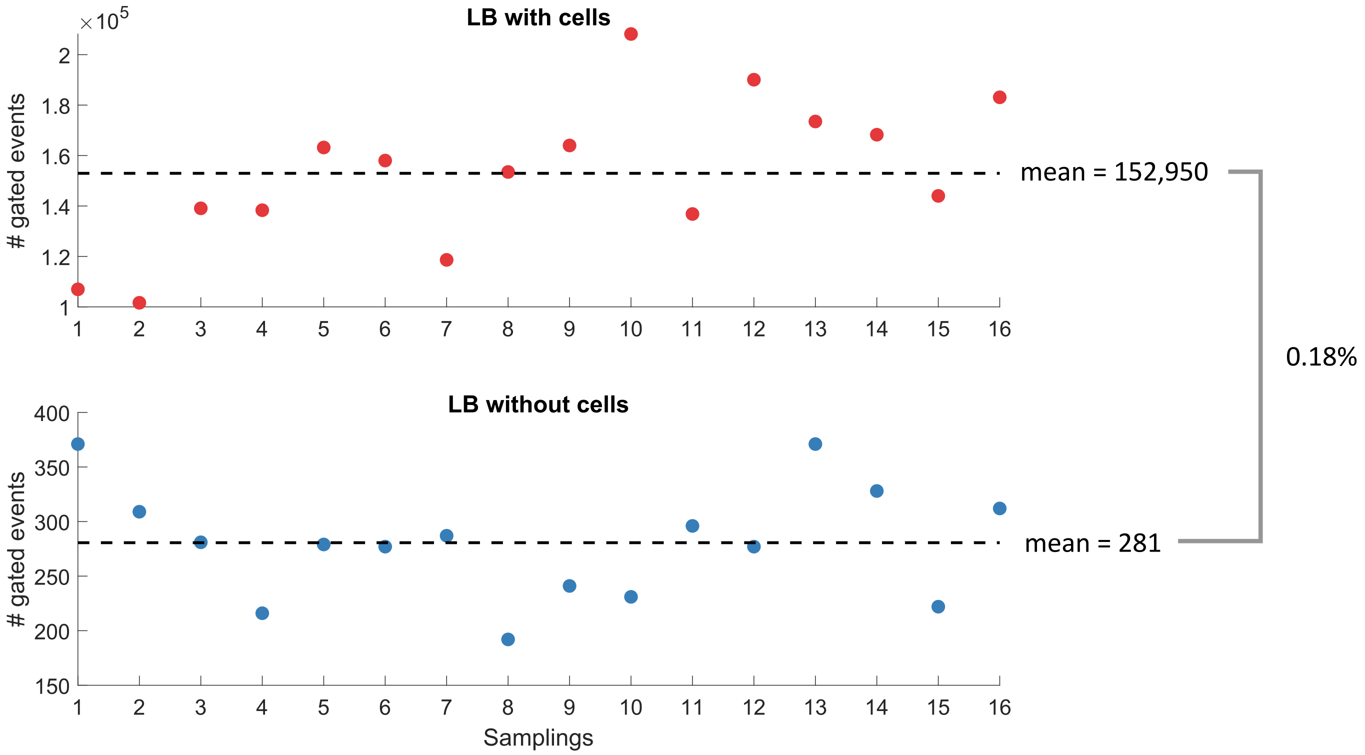

Supplementary Fig. 5. Sampling with *evotron* platform exhibits negligible cross-contamination
This figure shows the measured flow-cytometry gated events (indicating cell counts) in multiple automated samplings, with our *evotron* sampler, from two parallel cultures. One culture contained *E. coli* cells in LB media while the other contained sterile LB media without cells. Samples from these two cultures were taken in an alternate fashion with automated cleaning steps between successive sampling attempts. The number of gated events measured from the LB culture samples without cells displayed only background events with no increasing trend, even after multiple alternate sampling of LB culture with cells. Moreover, overnight incubation of the vial without cells after the 16th sampling cycle resulted in no bacterial growth, indicating that there had been no cross-contamination. Source data are provided as a Source Data file.

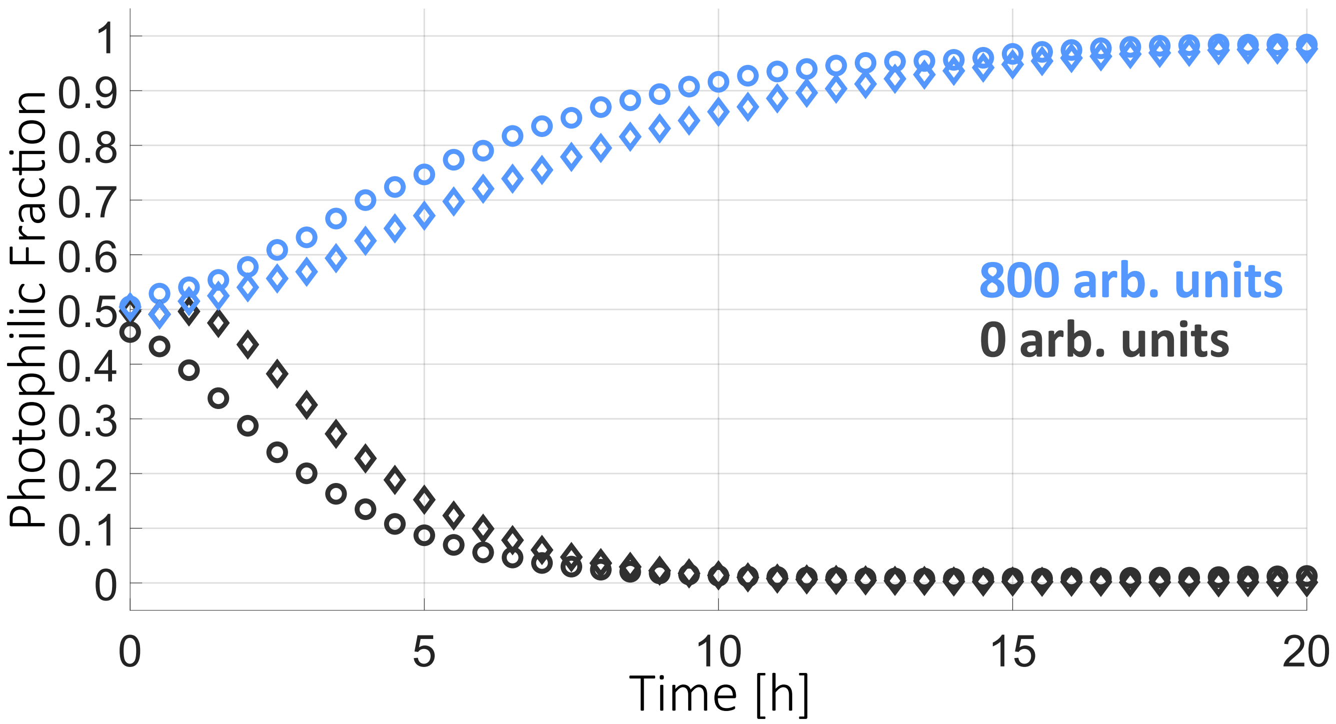

Supplementary Fig. 6. Open-loop co-culture: replicates
Biological replicates of the open-loop co-cultures with maximal light exposure or no light exposure shown in Figure 5b. Different symbols correspond to experiments carried out on different days. Source data are provided as a Source Data file.

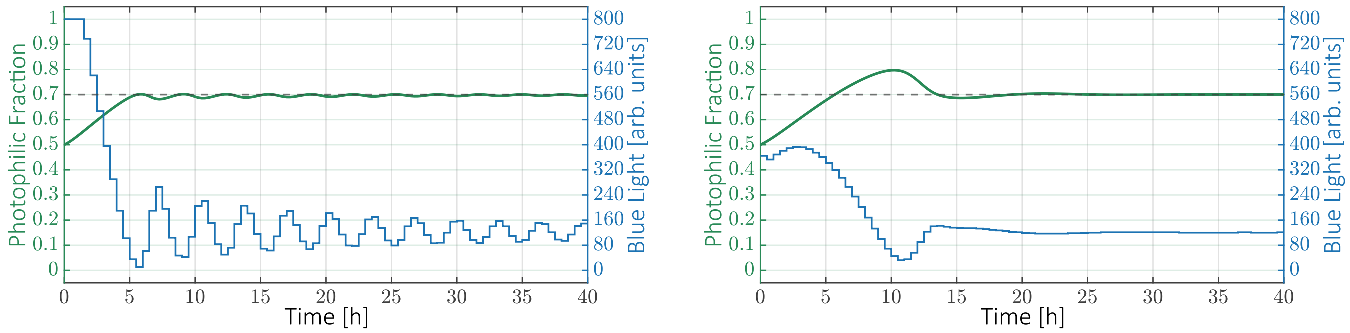

Supplementary Fig. 7. Different sets of optimal PID gains
The computational optimization procedure makes it possible to select gains that optimize different aspects of the dynamic behavior of the closed-loop system. **(Left)** The gains used in the closed-loop experiments produce a fast transient at the cost of low-amplitude oscillations at steady-state ($K_p = 5.9055 \cdot 10^3$, $K_i = 3.0382$, $K_d = 2.3427 \cdot 10^5$, $K_{bc} = 0.01 \cdot K_i$). **(Right)** An alternative set of gains results in no steady-state oscillations but after a longer transient phase with a large overshoot ($K_p = 1.5327 \cdot 10^3$, $K_i = 9.6743$, $K_d = 9.5689 \cdot 10^4$, $K_{bc} = 0.01 \cdot K_i$).

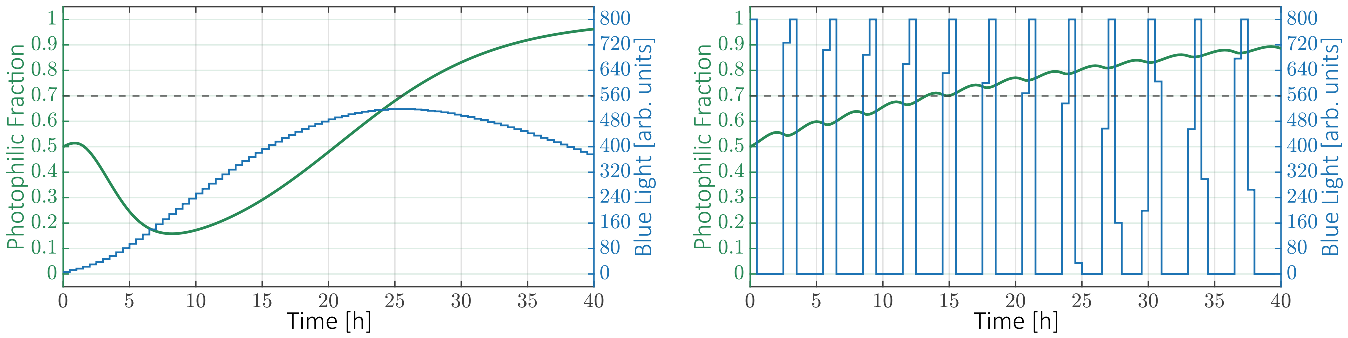

Supplementary Fig. 8. Un-optimized PID gains lead to poor dynamic performance of the closed-loop system
An arbitrary choice of gains lead to poor dynamic performance. Since the space of possible values for the gains is large, it is impractical to find a suitable set of gains through experimental trial-and-error. Model-guided optimization procedures can vastly reduce the number of experiments required to achieve optimal performance. **(Left)** $K_p = 1$, $K_i = 1$, $K_d = 1$, $K_{bc} = 1 \cdot K_i$. **(Right)** $K_p = 1 \cdot 10^5$, $K_i = 1$, $K_d = 1$, $K_{bc} = 1 \cdot K_i$.

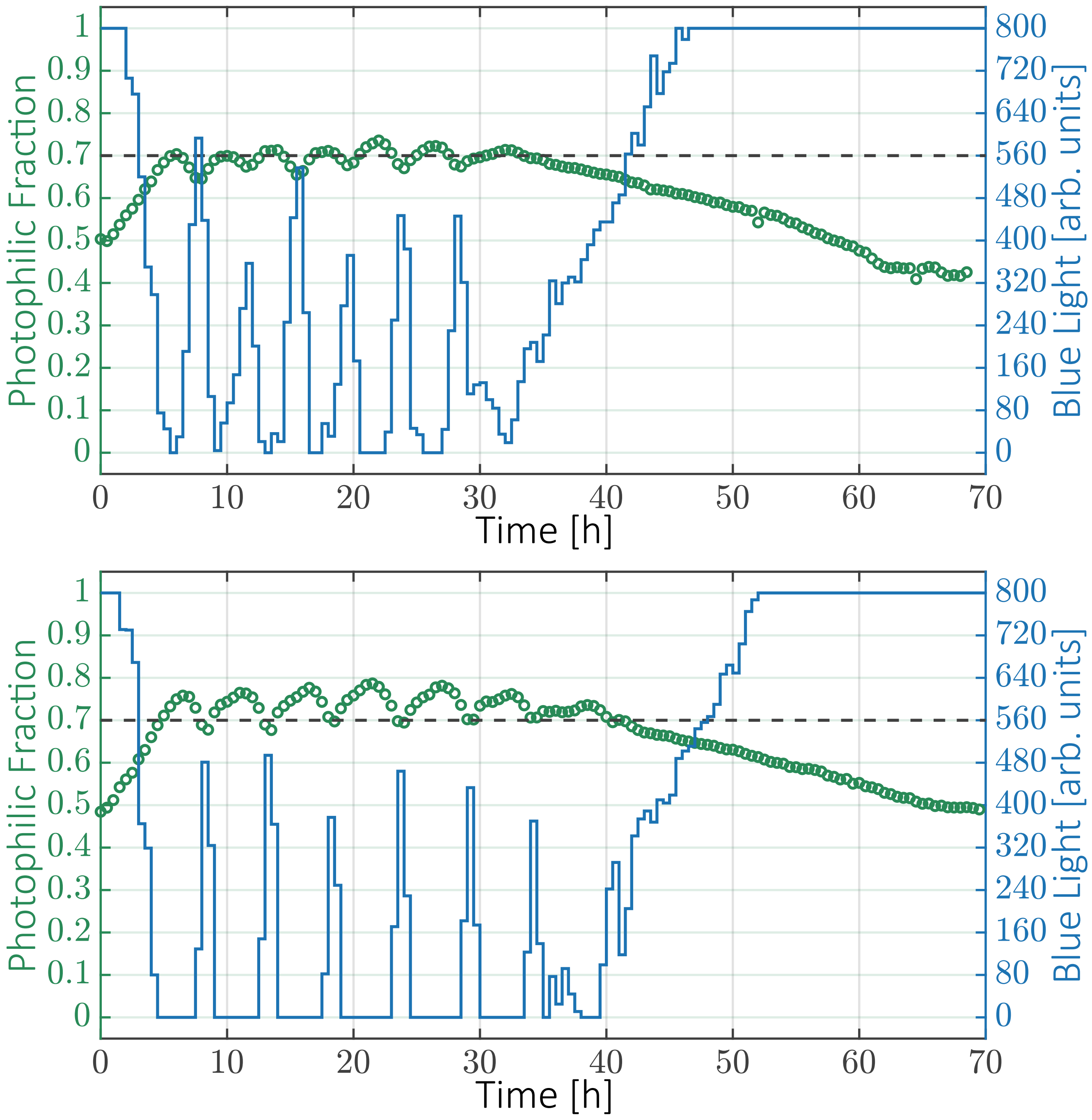

Supplementary Fig. 9. High-setpoint: replicates
Biological replicates of the high-setpoint closed-loop co-culture shown in Figure 6c. Source data are provided as a Source Data file.

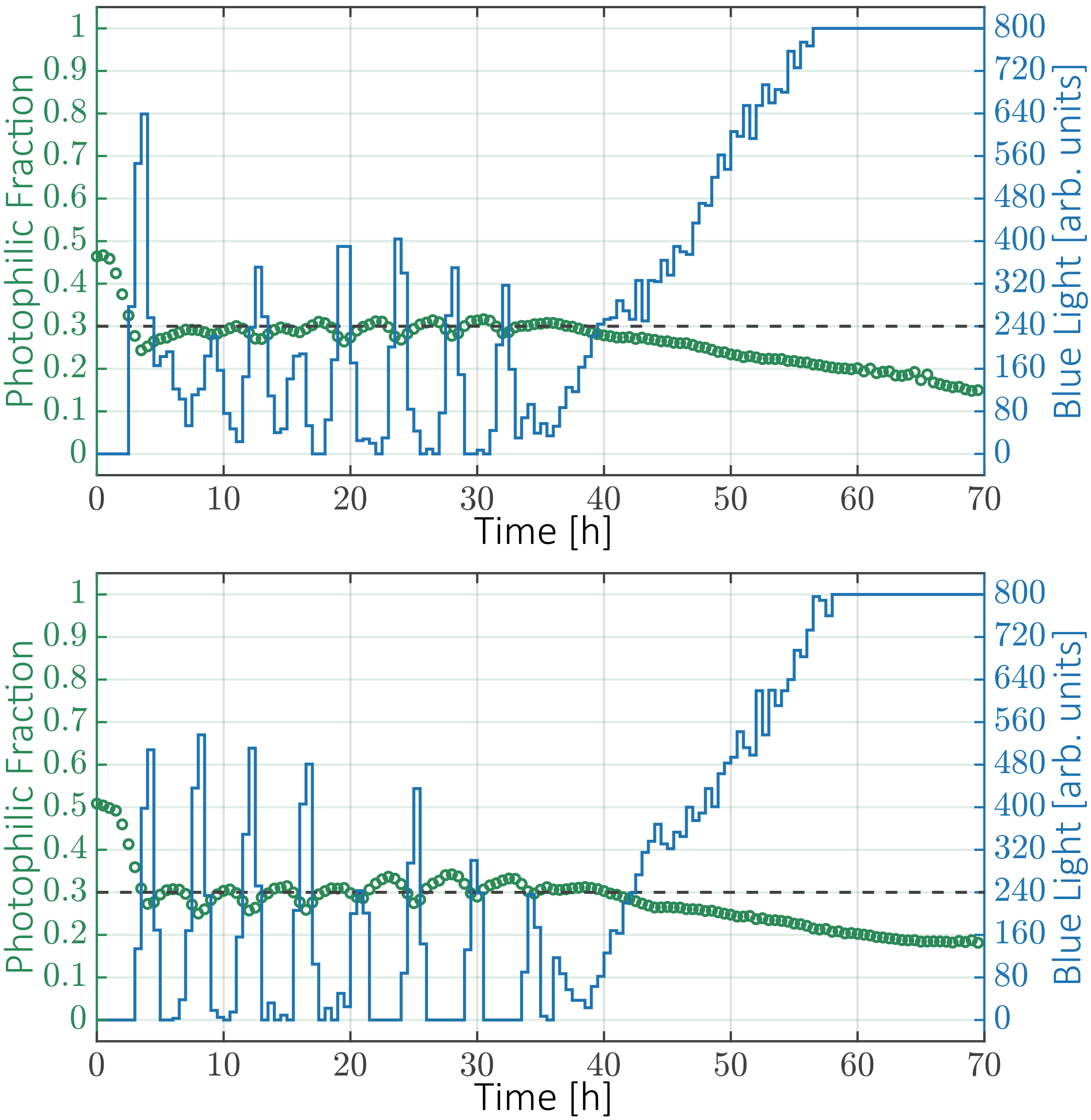

Supplementary Fig. 10. Low-setpoint: replicates
Biological replicates of the low-setpoint closed-loop co-culture shown in Figure 6d. Source data are provided as a Source Data file.

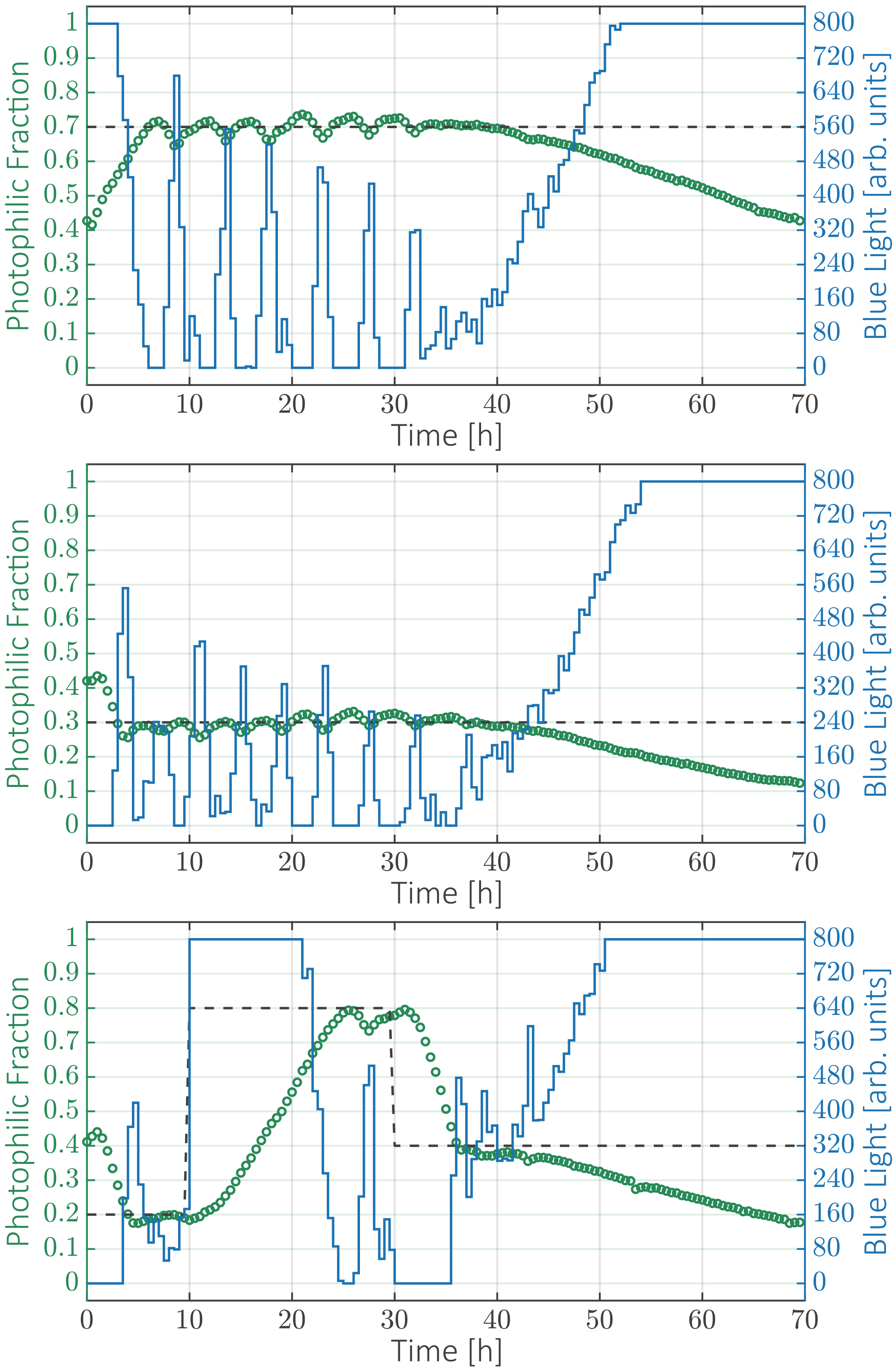

Supplementary Fig. 11. Breakdown of stabilization after 40h
After 40h we observe a continuous drift of the co-culture strain ratio in favor of the constitutive strain, probably reflecting the fixation of escape mutations in one of the strains or in both. The drift cannot be fully counteracted by the action of the controller, because change in growth rates due to mutations takes the closed-loop system out of the controllable regime. However, it can also be seen that the drift is much slower than what is observed in the absence of regulation (Figure 5), so that even 30h after the onset of the drift phase, the two strains still co-exist. We hypothesize that this is due to the fact that favorable mutations lead both strains to grow at the fastest attainable rate, which will be similar in both cases due to the low metabolic burden imposed by the growth control circuit in the photophilic strain. Source data are provided as a Source Data file.

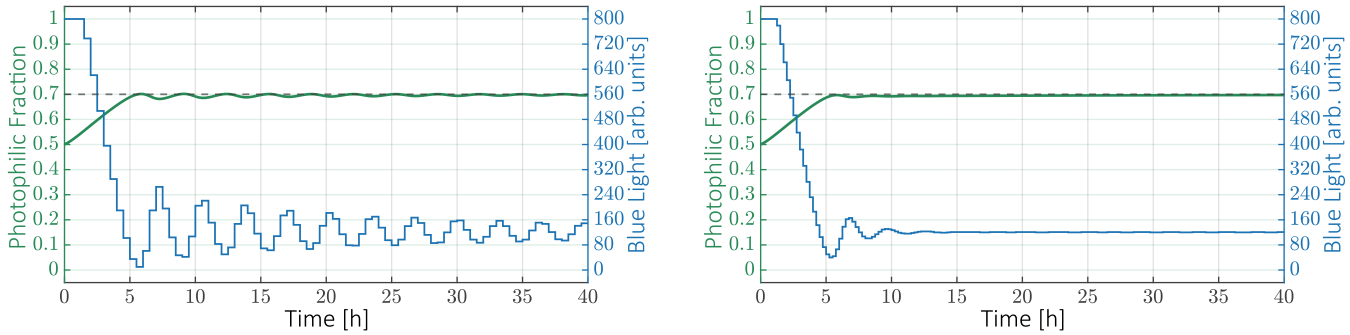

Supplementary Fig. 12. Sampling frequency and stability
The mathematical model suggests that the low-amplitude oscillations at steady-state observed in our closed-loop experiments (Figure 6) could be avoided if the sampling frequency is increased. **(Left)** Sampling every 30 minutes. **(Right)** Sampling every 15 minutes.

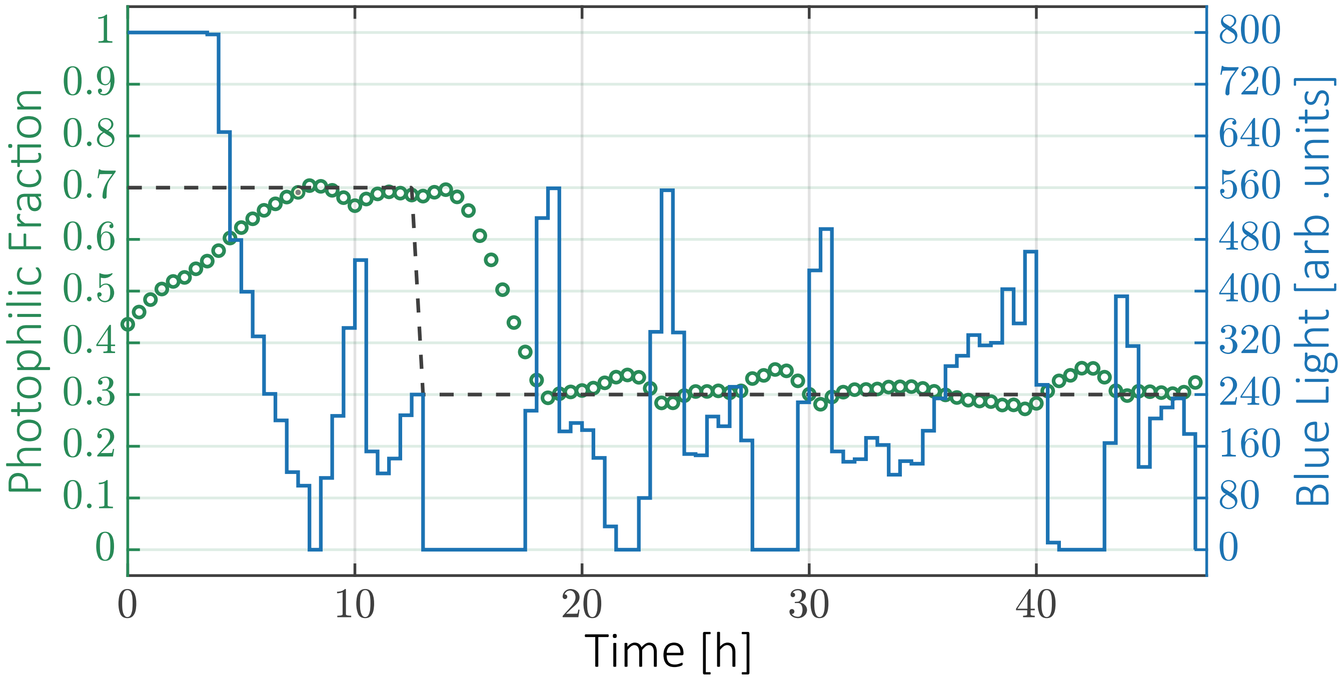

Supplementary Fig. 13. Setpoint tracking
As shown in Figure 6, the closed-loop co-culture can be forced to track a dynamically changing setpoint. In this case, the objective was to set the strain ratio to 0.7 during the first 13h and then to change it to 0.3 for the remainder of the experiment. Source data are provided as a Source Data file.

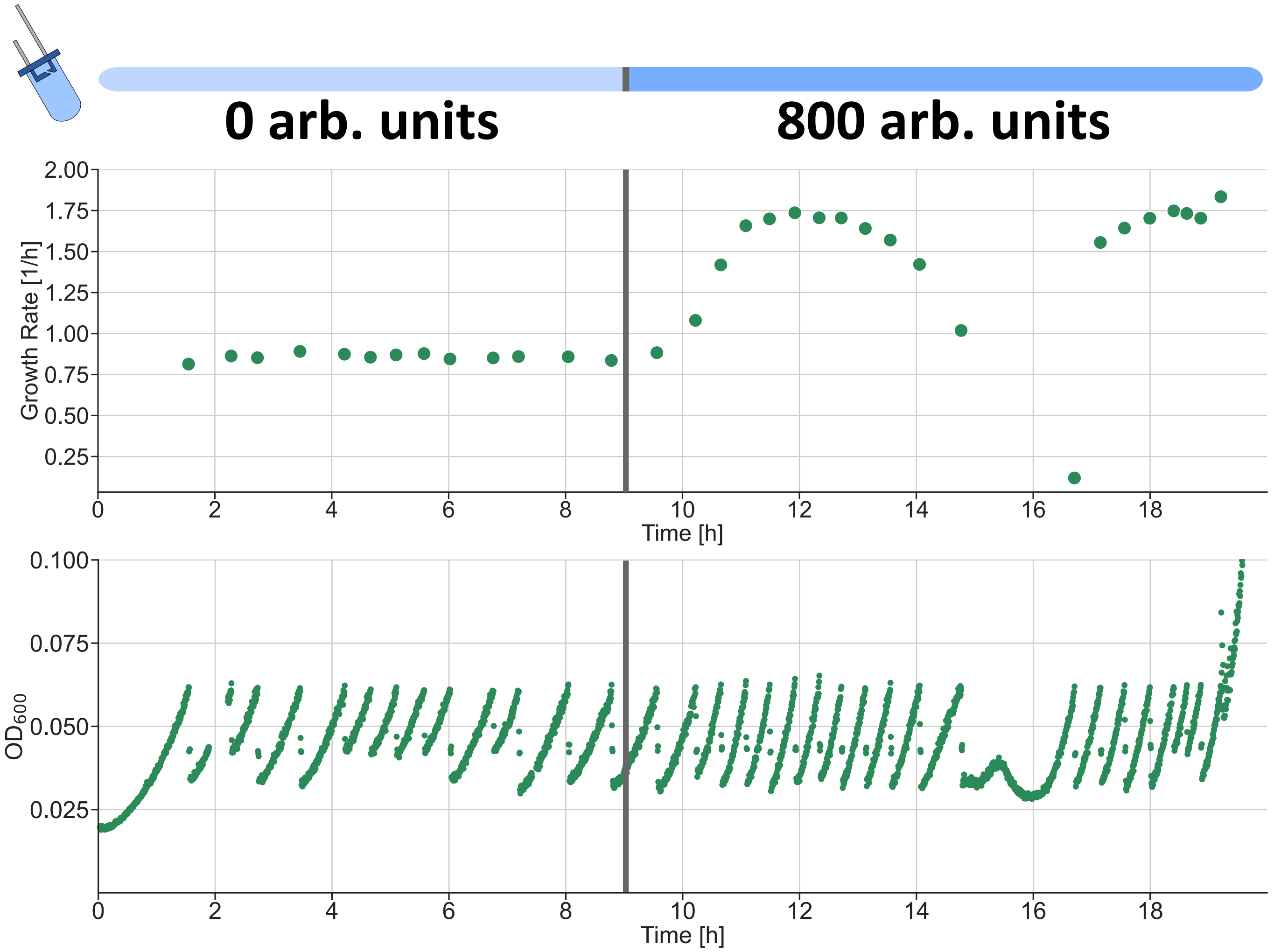

Supplementary Fig. 14. Growth arrest on *evotron* platform
After the *evotron* platform was assembled, we began observing a spontaneous, reversible growth arrest in the turbidostat cultures happening after 10-15h. The growth arrest happened at earlier times when the cells were grown with maximal light from the beginning, suggesting that the metabolic state of the cells influenced the timing of the arrest. Since this platform consists of a closed opentrons OT-2 robot (tightly covered with black foil to avoid ambient light inside) with a modified eVOLVER platorm replacing its deck (Supplementary Figure 23), we hypothesize that this growth arrest was caused by insufficient aeration within the covered OT-2 enclosure. Addition of an external source of pressurized air into the chamber restored normal growth (Supplementary Figure 15) and the growth defect was never observed when the air supply was running. Source data are provided as a Source Data file.

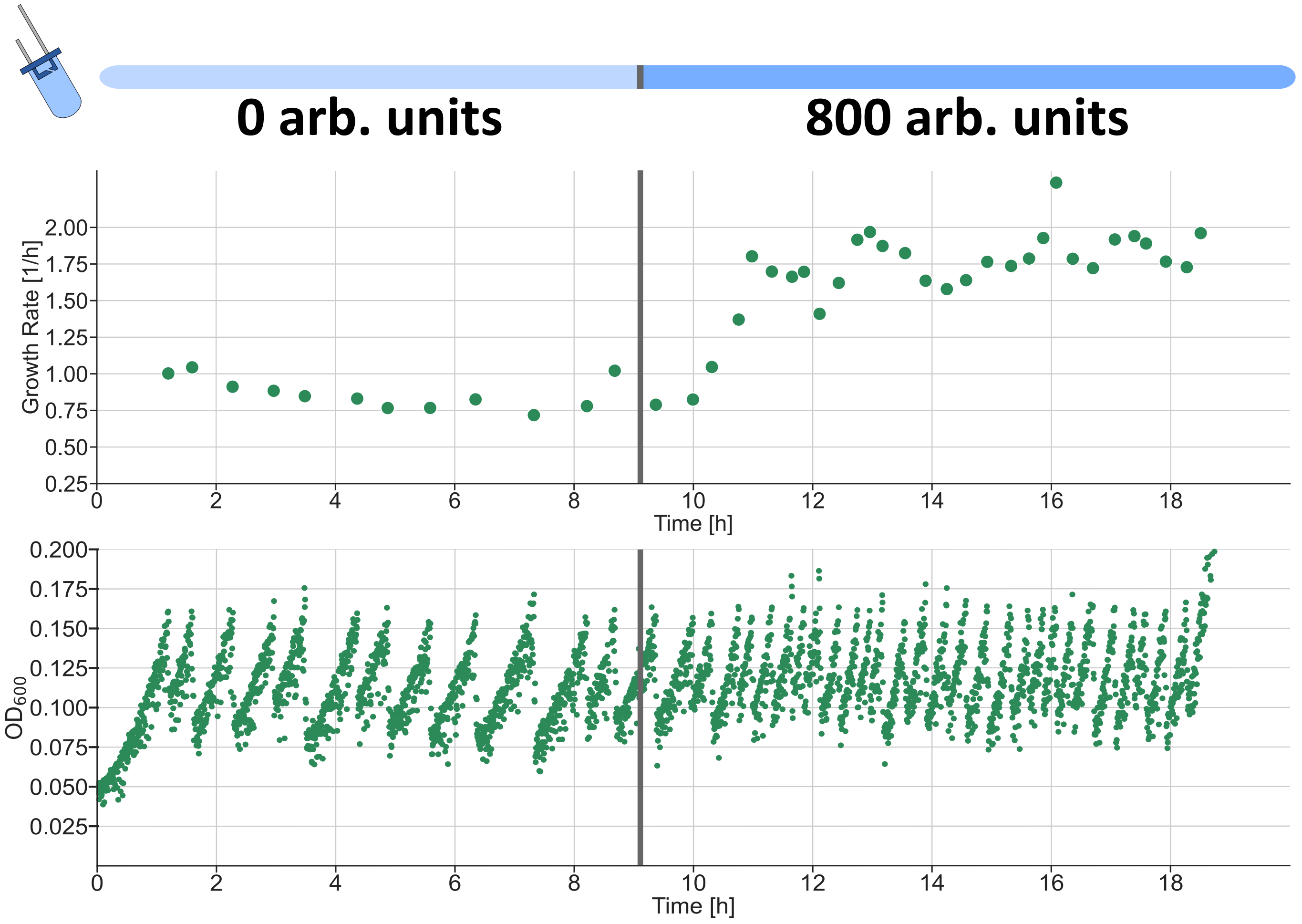

Supplementary Fig. 15. Aeration restores normal growth on *evotron* platform
Addition of an external source of pressurized air (4 bar) into the covered OT-2 enclosure restored normal growth in the turbidostat cultures. The data in this figure corresponds to an experiment analogous to the one shown in Supplementary Figure 14, but where the external supply of air was active throughout the experiment. The OD tolerance range of the turbidostat is different between the two experiments, but we observed that this was not the cause of the growth arrest. Source data are provided as a Source Data file.

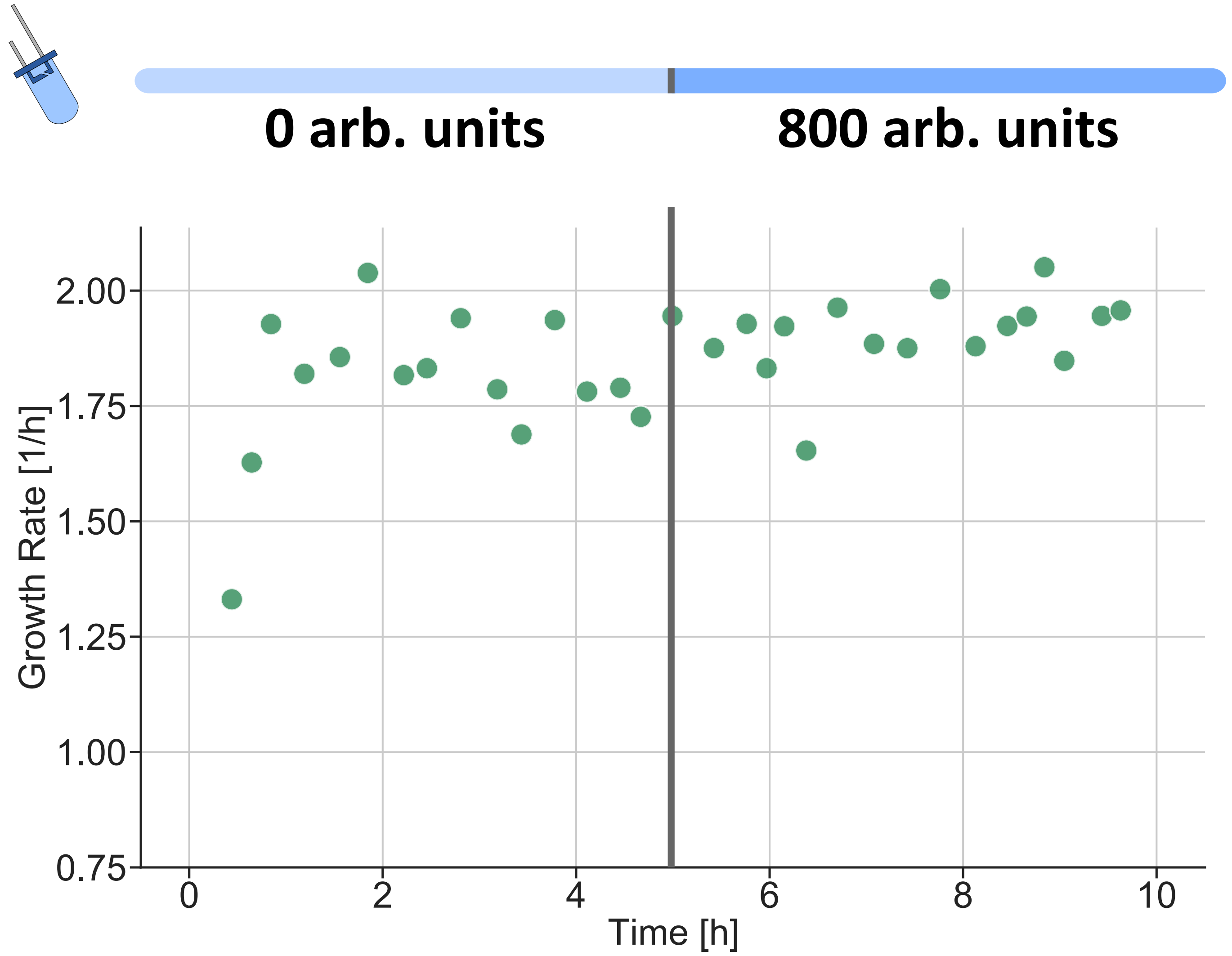

Supplementary Fig. 16. Growth of photophilic strain is not affected by light in the absence of chloramphenicol The photophilic strain was grown in the absence of chloramphenicol and the culture was grown in the dark for 5h and then illuminated with maximum light intensity. Light does not cause any appreciable difference in the growth rate, suggesting that the induction of the resistance causes negligible metabolic burden and that there is no direct phototoxicity. Source data are provided as a Source Data file.

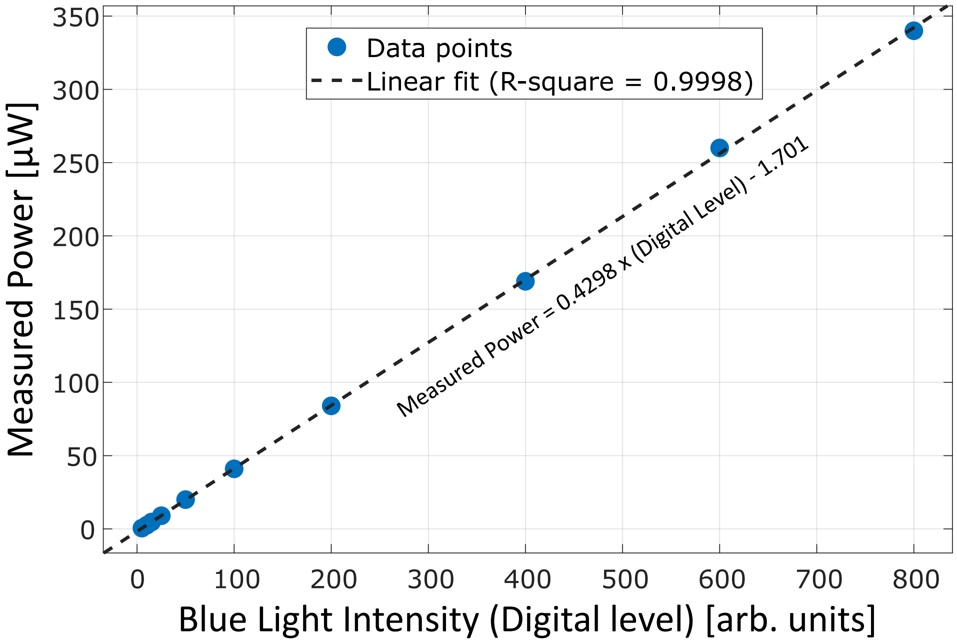

Supplementary Fig. 17. LED Measured power vs Digital level
We measured the blue LED (465nm) power inside the eVOLVER sleeve at different digital levels for characterization. Data shows a linear relation between the measured power and the applied digital level. LED power was measured with Nova power meter (PD300 sensor), Ophir Optronics Solutions Ltd. Source data are provided as a Source Data file.

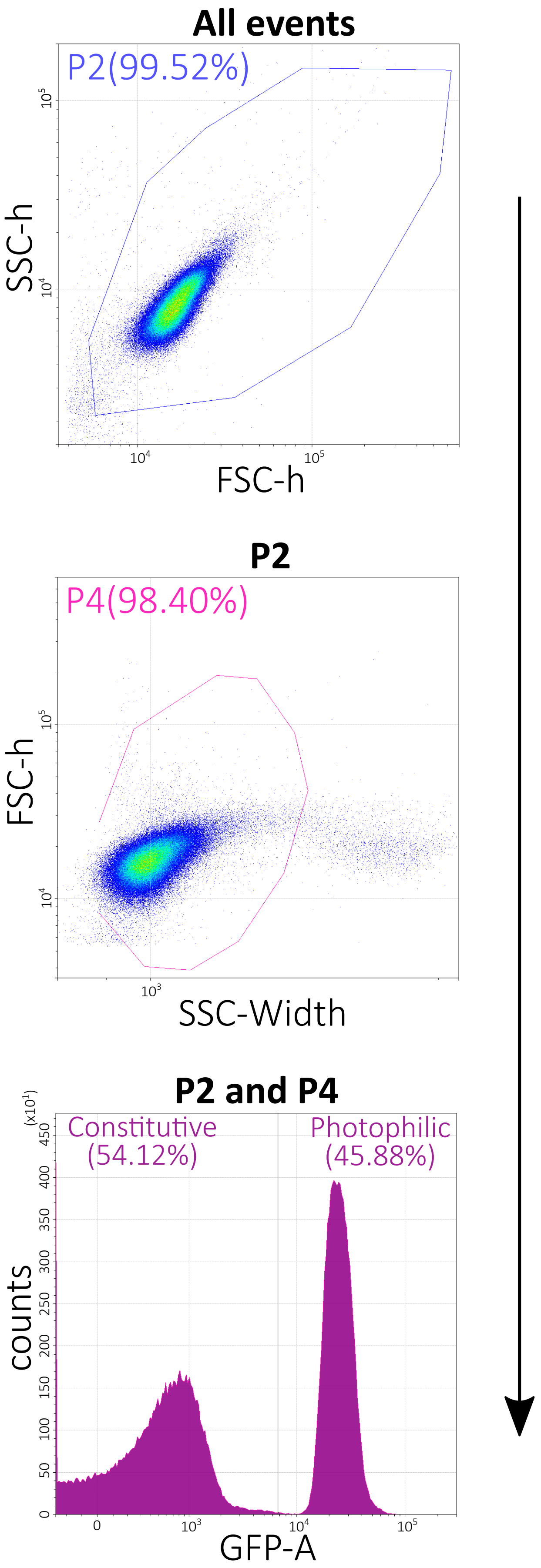

Supplementary Fig. 18. Flow-cytometry gating strategy
Gating strategy used to distinguish photophilic and constitutive cells and determine the co-culture strain ratio. First a polygon gate was applied on the FCS-H vs. SSC-H channels to select for living cells (P2). P2 was then further gated in the SSC-Width vs. FSC-H channels to select for single cells (P4). In co-culture experiments, the P4 population was further separated into two subpopulations (corresponding to the photophilic and constitutive strains) by applying a fixed threshold gate on the GFP-A channel (Threshold=6500).

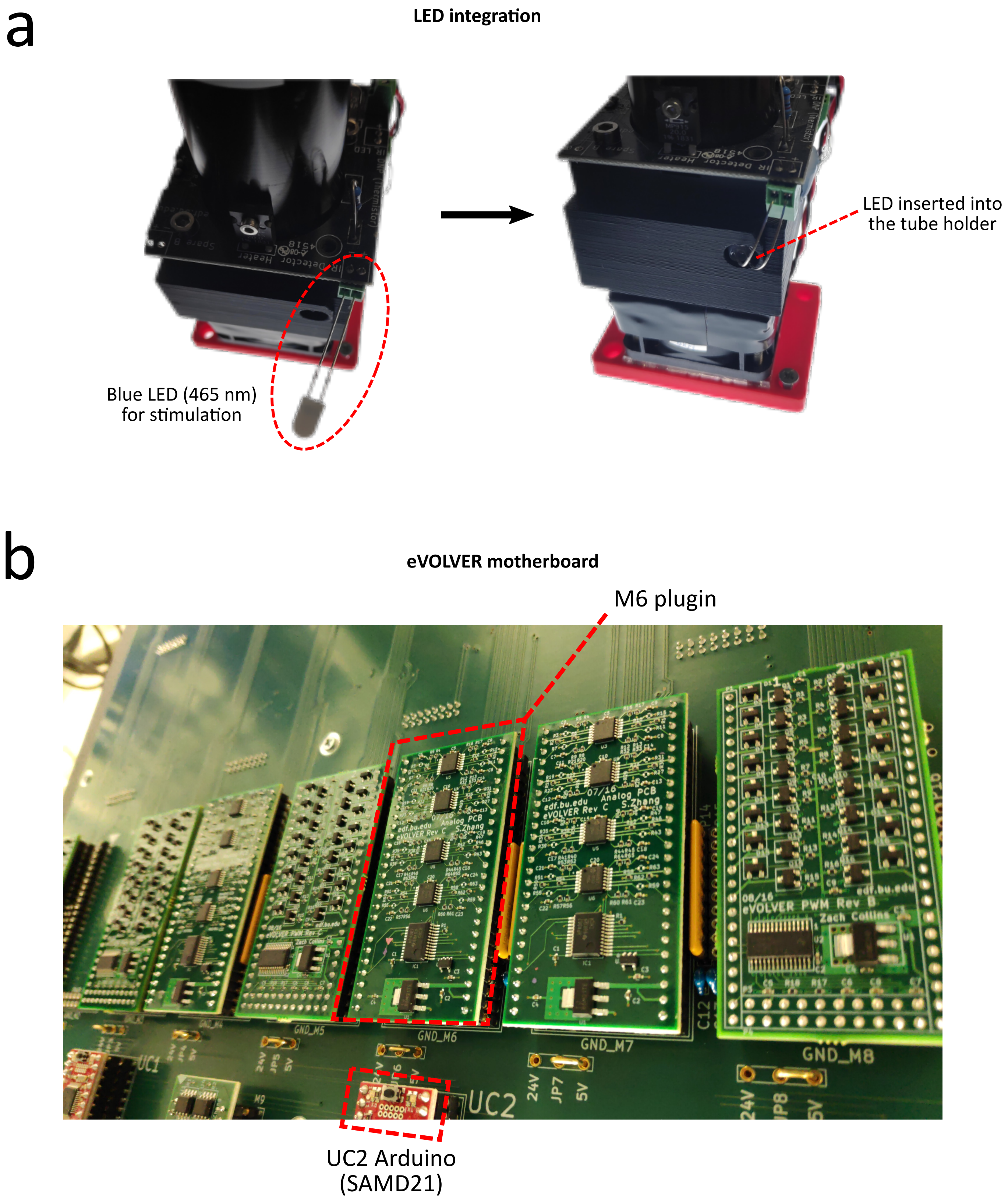

Supplementary Fig. 19. LED integration on the modified eVOLVER sleeve.
(a) LED for cell culture stimulation is connected to the sleeve electronic board at the 90 degree photodiode (IR detector) location. **(b)** To control and power this additional LED, M6 plugin on the eVOLVER motherboard is replaced with eVOLVER PWM board (1). UC2 SAMD21 arduino is re-programmed with suitable code accordingly, providing software control access for changing LED intensity during optogenetic experiments.

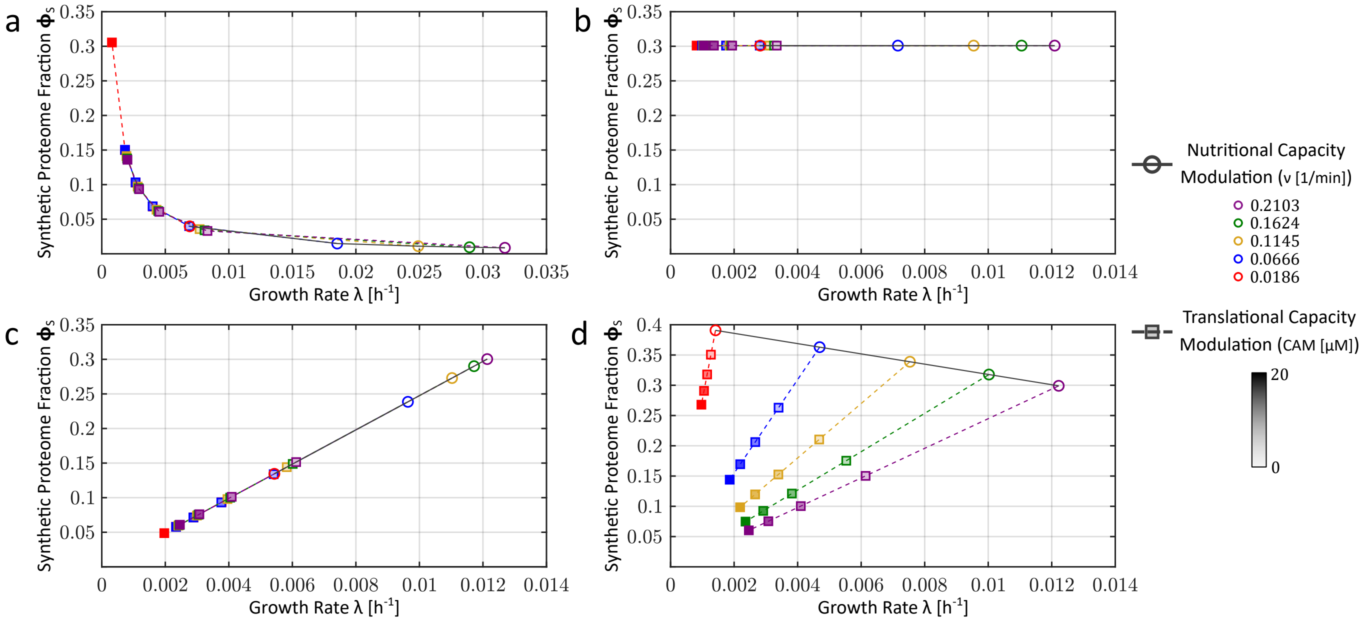

Supplementary Fig. 20. Model predictions for the dependency of the expression of a constitutive gene on growth rate The proteome fraction occupied by the constitutive protein is plotted against the growth rate. In the model, growth is varied by two independent mechanisms: by changing the quality of nutrients (nutritional capacity, circles of different colors) or by adding sublethal concentrations of chloramphenicol (translational capacity, squares with shading that denotes CAM concentration). **(a)** Conventional non-host-aware model. **(b)** Model that includes translational resources. **(c)** Model that includes both transcriptional and translational resources. **(d)** Model that includes both transcriptional and translational resources, as well as an explicit dependency on the nutritional capacity ν.

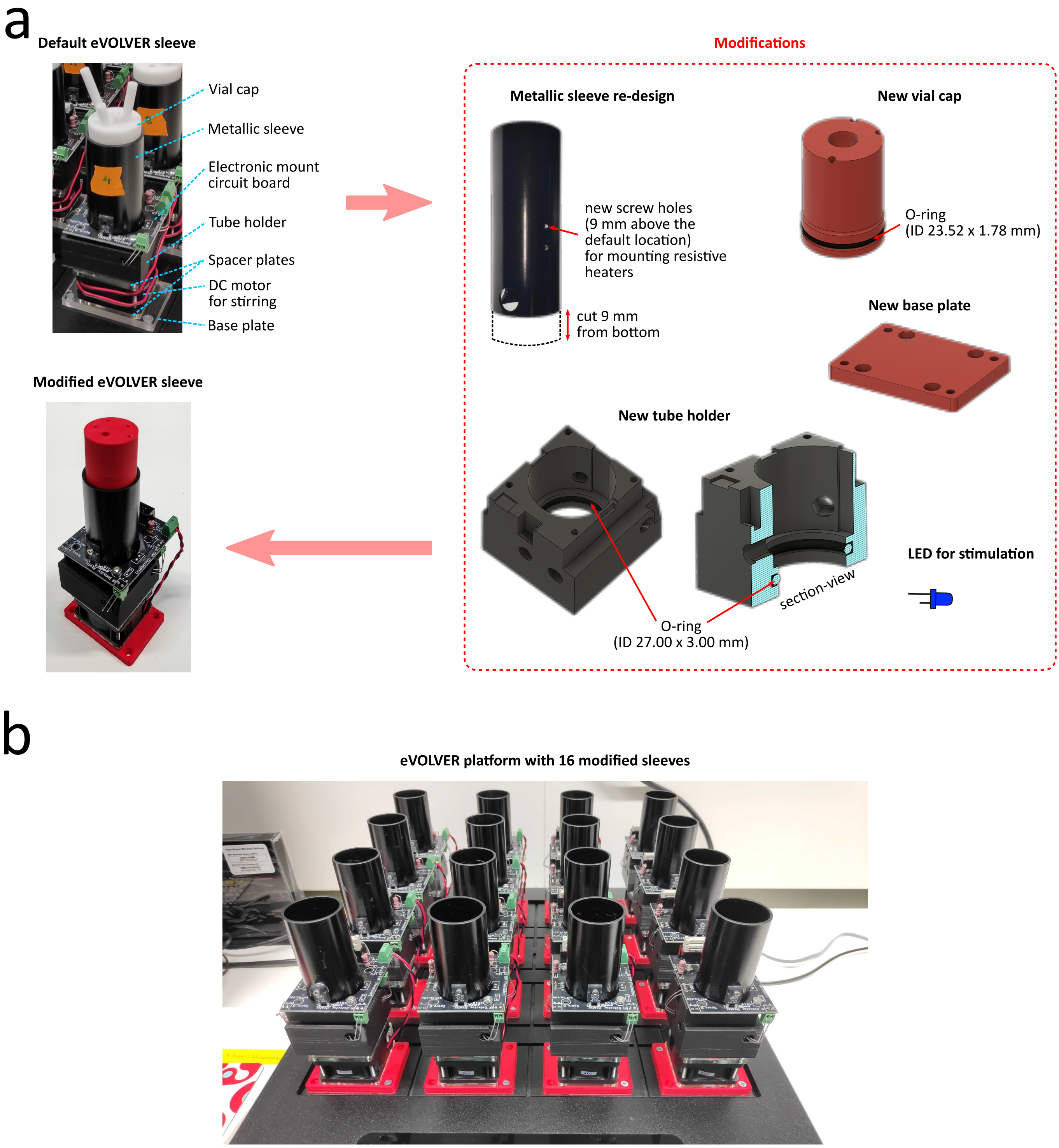

Supplementary Fig. 21. Modified eVOLVER sleeve design.
(a) Illustrated modifications resulted in stable and consistent OD measurement (as shown in Supplementary Figure 3) along with cell culture light stimulation capability. **(b)** eVOLVER platform with 16 modified sleeves.

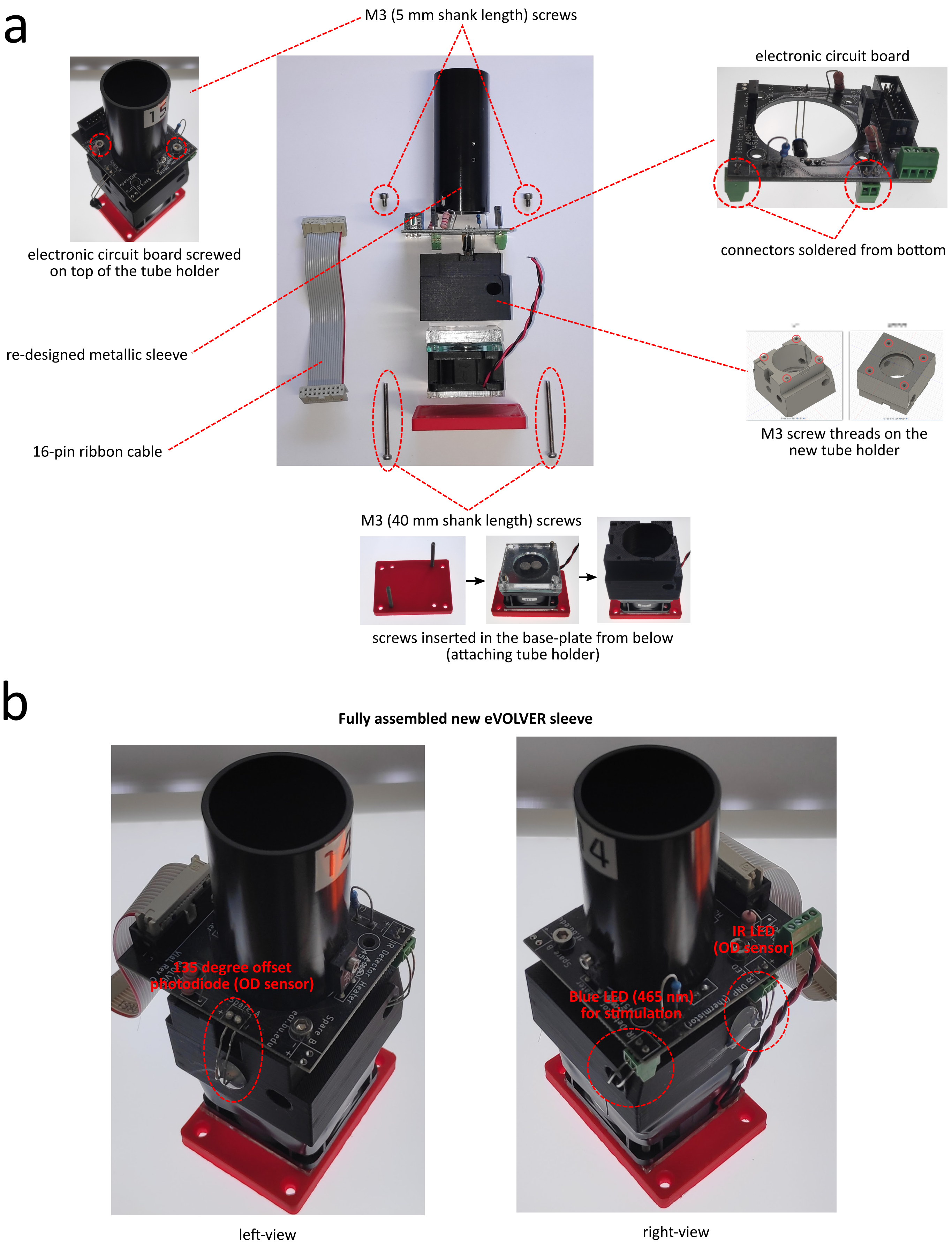

Supplementary Fig. 22. Modified eVOLVER sleeve assembly.
(a) Different components of the modified eVOLVER sleeve. **(b)** Fully assembled new eVOLVER sleeve with OD sensor (IR LED and photodiode) and blue LED for stimulation. This blue LED is connected to the electronic circuit board via a connector which was originally used for 90 degree offset photodiode in the default eVOLVER sleeve design.

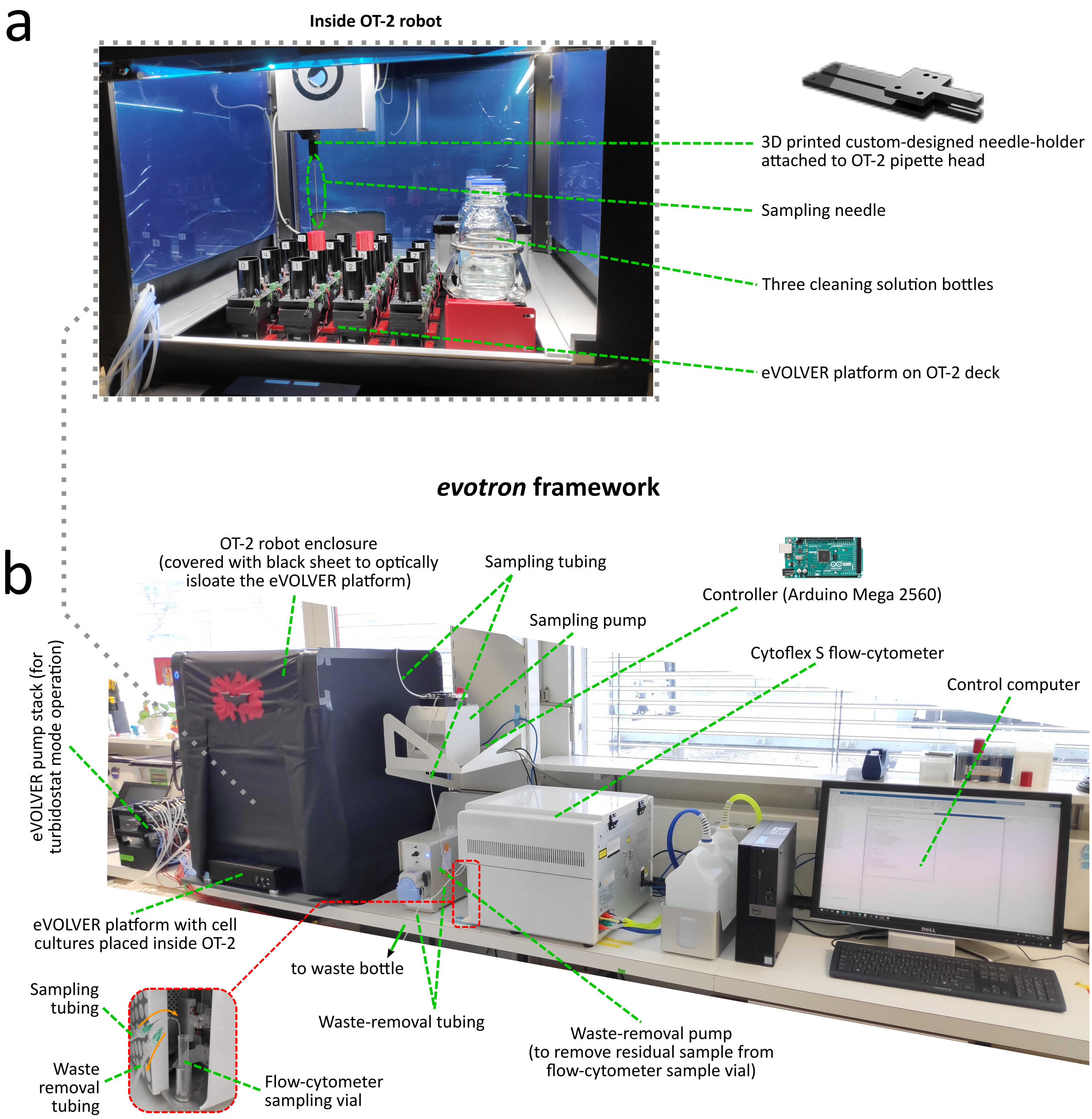

Supplementary Fig. 23. ***evotron*** **framework**
(a) Modfied eVOLVER platform placed inside Opentrons OT-2 robot **(b)** Complete *evotron* framework assembled on a lab bench.

Parameter	Description	Value	Unit	Source
$\hat{\alpha}_T$	Effective T7-monomer production rate	$7.2 \cdot 10^{-3}$	nM	⋆
$\hat{\alpha}_C$	Effective CAT production rate	$6.467 \cdot 10^{-6}$	$\mathrm{nM\,min^{-1}}$	⋆
K_G	K_m of T7-promoter activation	57.1585	nM	⋆
h_{ON}^{min}	Minimal dimerization rate	$2.0202 \cdot 10^{-7}$	$\mathrm{nM^{-1}\,min^{-1}}$	⋆
h_{ON}^{max}	Maximal dimerization rate	$2.0020 \cdot 10^{-5}$	$\mathrm{nM^{-1}\,min^{-1}}$	⋆
K_L	Light-dependent dimerization's K_m	$1.9851 \cdot 10^{3}$	arb. units∗	⋆
n_L	Light-dependent dimerization's Hill coefficient	1.3548		⋆
n_G	Hill coefficient of T7-promoter activation	1.5557		⋆
h_C	Hill-exponent of CAM degradation	1.5388		⋆
$\gamma_{\mathrm{mol2fluo}}$	Conversion factor between molecule numbers and flow-cytometry arbitrary mCherry fluorescence units	0.2549	$\mathrm{nM^{-1}}$ arb. units ∗∗	⋆
L_0	Effective light-intensity corresponding to ambient light during pre-culturing	196.3930	arb. units∗	⋆
N_p	p15A plasmid copy number (CAT gene)	10		(15) BNID: 105307
K_D	K_m of CAM-ribosome binding reaction	1300	nM	(8)
$\hat{K}_C$	K_m of CAM-ribosome binding reaction	0.3333	nM min	(8)
κ	CAM diffusion rate	90	$\mathrm{min^{-1}}$	(8)
n_r	effective ribosomal unit length	12221	aa	Estimated from (5)
n_T	T7-monomer length	597	aa	‡
n_C	CAT length	219	aa	
g_0	Average translation rate per ribosome	0.0987	$\mathrm{min^{-1}}$	Estimated from (5)
Φ_R^{max}	Maximal ribosomal proteome fraction	0.5470		(5)
Φ_{R_0}	Proteome fraction of inactive ribosomes	0.0660		(5)
ρ_{cell}	Cell density (conversion factor between proteome fractions and molecules per cell	$2 \cdot 10^{9}$	$\mathrm{aa\,fL^{-1}}$	(4)
ν	Nutrient capacity of medium (LB)	0.1921	$\mathrm{min^{-1}}$	†
A_E	External concentration of CAM	$10.5 \cdot 10^{3}$	nM	

Supplementary Table 1. Parameter values used for simulations of photophilic strain and co-culture dynamics ⋆Obtained from fit to the photophilic strain's dynamic response. †Obtained from manual fitting to the growth rate of the photophilic strain in the absence of CAM. ‡Average between the length of the two T7 split units (16). ∗Arbitrary units of light intensity. ∗∗Arbitrary units of flow-cytometry mCherry fluorescence.

3 Plasmid sequences used in this study

Strain	Host	Plasmids
bJAG132 (photophilic)	SKA1515 (BW25113 attB::venus)	pAB276, mJAG063
bJAG234	BW25113	mJAG090
bJAG235 (constitutive)	BW25113	mJAG090, mJAG099
bJAG236	BW25113	mJAG090, mJAG100
bJAG237	BW25113	mJAG090, mJAG101
bJAG367	BW25113	mJAG019, mJAG168

Supplementary Table 2. Strains and plasmids used in this study

In the following, nucleotide sequences for the plasmids used in this study and listed in Supplementary Table 2 are provided in genbank format. Saving each text entry into a file with .gb extension and importing it into a sequence reader renders a fully-annotated plasmid map.

```
LOCUS       pAB276                 7559 bp ds-DNA     circular     28-JUL-2022
DEFINITION  .
FEATURES             Location/Qualifiers
     rep_origin      complement(368..729)
                     /label="pSC101 ori"
     terminator      complement(746..840)
                     /label="lambda t0 terminator"
     CDS             complement(870..1730)
                     /label="bla"
     misc_feature    1867..1939
                     /label="araB promoter"
     RBS             1954..1960
                     /label="RBS"
     misc_feature    1969..3657
                     /label="T7 Pol (1-563)"
     misc_feature    3658..3672
                     /label="long gly-ala linker"
     misc_feature    3673..4122
                     /label="nMAGHigh1 (VVD)"
     terminator      4132..4229
                     /label="rrnB T1 terminator"
     misc_feature    4720..4792
                     /label="araB promoter"
     RBS             4842..4848
                     /label="RBS"
     misc_feature    4887..5336
                     /label="pMag (VVD1)"
     misc_feature    5337..5351
                     /label="long gly-ala linker"
     misc_feature    5352..6311
                     /label="T7 Pol (564-883)"
     misc_feature    6596..7543
                     /label="repA"
ORIGIN
        1 AGGTGATTTT AATCACTATA CCAATTGAGA TGGGCTAGTC AATGATAATT ACTAGTCCTT
       61 TTCCTTTGAG TTGTGGGTAT CTGTAAATTC TGCTAGACCT TTGCTGGAAA ACTTGTAAAT
      121 TCTGCTAGAC CCTCTGTAAA TTCCGCTAGA CCTTTGTGTG TTTTTTTTGT TTATATTCAA
      181 GTGGTTATAA TTTATAGAAT AAAGAAAGAA TAAAAAAAGA TAAAAAGAAT AGATCCCAGC
      241 CCTGTGTATA ACTCACTACT TTAGTCAGTT CCGCAGTATT ACAAAAGGAT GTCGCAAACG
      301 CTGTTTGCTC CTCTACAAAA CAGACCTTAA AACCCTAAAG GCTTAAGTAG CACCCTCGCA
      361 AGCTCGGGCA AATCGCTGAA TATTCCTTTT GTCTCCGACC ATCAGGCACC TGAGTCGCTG
      421 TCTTTTTCGT GACATTCAGT TCGCTGCGCT CACGGCTCTG GCAGTGAATG GGGGTAAATG
      481 GCACTACAGG CGCCTTTTAT GGATTCATGC AAGGAAACTA CCCATAATAC AAGAAAAGCC
      541 CGTCACGGGC TTCTCAGGGC GTTTTATGGC GGGTCTGCTA TGTGGTGCTA TCTGACTTTT
      601 TGCTGTTCAG CAGTTCCTGC CCTCTGATTT TCCAGTCTGA CCACTTCGGA TTATCCCGTG
      661 ACAGGTCATT CAGACTGGCT AATGCACCCA GTAAGGCAGC GGTATCATCA ACAGGCTTAC
      721 CCGTCTTACT GTCCCTAGTG CTTGGATTCT CACCAATAAA AAACGCCCGG CGGCAACCGA
      781 GCGTTCTGAA CAAATCCAGA TGGAGTTCTG AGGTCATTAC TGGATCTATC AACAGGAGTC
      841 CAAGCGAGCT CGTAAACTTG GTCTGACAGT TACCAATGCT TAATCAGTGA GGCACCTATC
      901 TCAGCGATCT GTCTATTTCG TTCATCCATA GTTGCCTGAC TCCCCGTCGT GTAGATAACT
      961 ACGATACGGG AGGGCTTACC ATCTGGCCCC AGTGCTGCAA TGATACCGCG AGACCCACGC
     1021 TCACCGGCTC CAGATTTATC AGCAATAAAC CAGCCAGCCG GAAGGGCCGA GCGCAGAAGT
     1081 GGTCCTGCAA CTTTATCCGC CTCCATCCAG TCTATTAATT GTTGCCGGGA AGCTAGAGTA
     1141 AGTAGTTCGC CAGTTAATAG TTTGCGCAAC GTTGTTGCCA TTGCTACAGG CATCGTGGTG
     1201 TCACGCTCGT CGTTTGGTAT GGCTTCATTC AGCTCCGGTT CCCAACGATC AAGGCGAGTT
     1261 ACATGATCCC CCATGTTGTG CAAAAAAGCG GTTAGCTCCT TCGGTCCTCC GATCGTTGTC
     1321 AGAAGTAAGT TGGCCGCAGT GTTATCACTC ATGGTTATGG CAGCACTGCA TAATTCTCTT
     1381 ACTGTCATGC CATCCGTAAG ATGCTTTTCT GTGACTGGTG AGTACTCAAC CAAGTCATTC
     1441 TGAGAATAGT GTATGCGGCG ACCGAGTTGC TCTTGCCCGG CGTCAATACG GGATAATACC
     1501 GCGCCACATA GCAGAACTTT AAAAGTGCTC ATCATTGGAA AACGTTCTTC GGGGCGAAAA
     1561 CTCTCAAGGA TCTTACCGCT GTTGAGATCC AGTTCGATGT AACCCACTCG TGCACCCAAC
     1621 TGATCTTCAG CATCTTTTAC TTTCACCAGC GTTTCTGGGT GAGCAAAAAC AGGAAGGCAA
```

1681 AATGCCGCAA AAAAGGGAAT AAGGGCGACA CGGAAATGTT GAATACTCAT ACTCTTCCTT
1741 TTTCAATATT ATTGAAGCAT TTATCAGGGT TATTGTCTCA TGAGCGGATA CATATTTGAA
1801 TGTATTTAGA AAAATAAACA AATAGGGGTT CCGCGCACAT TTCCCCGAAA AGTGCCACCT
1861 GACGTCTAGC ATTTTTATCC ATAAGATTAG CGGATCCTAC CTGACGCTTT TTATCGCAAC
1921 TCTCTACTGT TTCTCCATAC CCGTTTTTTT GGAAGGAGGT AAAGATCTAT GAACACGATT
1981 AACATCGCTA AGAACGACTT CTCTGACATC GAACTGGCTG CTATCCCGTT CAACACTCTG
2041 GCTGACCATT ACGGTGAGCG TTTAGCTCGC GAACAGTTGG CCCTTGAGCA TGAGTCTTAC
2101 GAGATGGGTG AAGCACGCTT CCGCAAGATG TTTGAGCGTC AACTTAAAGC TGGTGAGGTT
2161 GCGGATAACG CTGCCGCCAA GCCTCTCATC ACTACCCTAC TCCCTAAGAT GATTGCACGC
2221 ATCAACGACT GGTTTGAGGA AGTGAAAGCT AAGCGCGGCA AGCGCCCGAC AGCCTTCCAG
2281 TTCCTGCAAG AAATCAAGCC GGAAGCCGTA GCGTACATCA CCATTAAGAC CACTCTGGCT
2341 TGCCTAACCA GTGCTGACAA TACAACCGTT CAGGCTGTAG CAAGCGCAAT CGGTCGGGCC
2401 ATTGAGGACG AGGCTCGCTT CGGTCGTATC CGTGACCTTG AAGCTAAGCA CTTCAAGAAA
2461 AACGTTGAGG AACAACTCAA CAAGCGCGTA GGGCACGTCT ACAAGAAAGC ATTTATGCAA
2521 GTTGTCGAGG CTGACATGCT CTCTAAGGGT CTACTCGGTG GCGAGGCGTG GTCTTCGTGG
2581 CATAAGGAAG ACTCTATTCA TGTAGGAGTA CGCTGCATCG AGATGCTCAT TGAGTCAACC
2641 GGAATGGTTA GCTTACACCG CCAAAATGCT GGCGTAGTAG GTCAAGACTC TGAGACTATC
2701 GAACTCGCAC CTGAATACGC TGAGGCTATC GCAACCCGTG CAGGTGCGCT GGCTGGCATC
2761 TCTCCGATGT TCCAACCTTG CGTAGTTCCT CCTAAGCCGT GGACTGGCAT TACTGGTGGT
2821 GGCTATTGGG CTAACGGTCG TCGTCCTCTG GCGCTGGTGC GTACTCACAG TAAGAAAGCA
2881 CTGATGCGCT ACGAAGACGT TTACATGCCT GAGGTGTACA AAGCGATTAA CATTGCGCAA
2941 AACACCGCAT GGAAAATCAA CAAGAAAGTC CTAGCGGTCG CCAACGTAAT CACCAAGTGG
3001 AAGCATTGTC CGGTCGAGGA CATCCCTGCG ATTGAGCGTG AAGAACTCCC GATGAAACCG
3061 GAAGACATCG ACATGAATCC TGAGGCTCTC ACCGCGTGGA AACGTGCTGC CGCTGCTGTG
3121 TACCGCAAGG ACAAGGCTCG CAAGTCTCGC CGTATCAGCC TTGAGTTCAT GCTTGAGCAA
3181 GCCAATAAGT TTGCTAACCA TAAGGCCATC TGGTTCCCTT ACAACATGGA CTGGCGCGGT
3241 CGTGTTTACG CTGTGTCAAT GTTCAACCCG CAAGGTAACG ATATGACCAA AGGACTGCTT
3301 ACGCTGGCGA AAGGTAAACC AATCGGTAAG GAAGGTTACT ACTGGCTGAA AATCCACGGT
3361 GCAAACTGTG CGGGTGTCGA TAAGGTTCCG TTCCCTGAGC GCATCAAGTT CATTGAGGAA
3421 AACCACGAGA ACATCATGGC TTGCGCTAAG TCTCCACTGG AGAACACTTG GTGGGCTGAG
3481 CAAGATTCTC CGTTCTGCTT CCTTGCGTTC TGCTTTGAGT ACGCTGGGGT ACAGCACCAC
3541 GGCCTGAGCT ATAACTGCTC CCTTCCGCTG GCGTTTGACG GGTCTTGCTC TGGCATCCAG
3601 CACTTCTCCG CGATGCTCCG AGATGAGGTA GGTGGTCGCG CGGTTAACTT GCTTCCTggc
3661 ggtTCTggag gtCACACTCT TTACGCCCCT GGAGGATACG ACATTATGGG ATATTTGGAT
3721 CAGATTGGGA ACCGCCCAAA CCCTCAGGTC GAACTGGGGC CTGTGGACAC GTCATGTGCC
3781 CTGATCCTGT GCGATCTGAA GCAAAAGGAC ACTCCGATCG TCTACGCCTC GGAAGCCTTC
3841 TTGTATATGA CCGGATACAG CAATGCAGAG GTGCTCGGCA GGAACTGCAG ATTCCTGCAG
3901 TCCCCCGACG GGATGGTGAA ACCAAAGTCG ACTCGCAAAT ATGTGGACTC GAACACGATC
3961 AACACCATCC GGAAGGCCAT CGACCGGAAC GCCGAGGTCC AGGTGGAGGT GGTCAACTTT
4021 AAGAAGAACG GCCAGCGGTT CGTGAACTTT CTGACCATCA TTCCGGTCCG GGATGAAACC
4081 GGAGAGTACA GATACTCCAT GGGATTCCAG TGCGAAACCG AATAACCTAG GAGGCATCAA
4141 ATAAAACGAA AGGCTCAGTC GAAAGACTGG GCCTTTCGTT TTATCTGTTG TTTGTCGGTG
4201 AACGCTCTCC TGAGTAGGAC AAATCCGCCG CCCTAGACCT AGCTGCAGCT AGGGTACGGG
4261 TTTTGCTGCC CGCAAACGGG CTGTTCTGGT GTTGCTAGTT TGTTATCAGA ATCGCAGATC
4321 CGGCTTCAGc cgGTTTGCCG GCTGAAAGCG CTATTTCTTC CAGAATTGCC ATGATTTTTT
4381 CCCCACGGGA GGCGTCACTG GCTCCCGTGT TGTCGGCAGC TTTGATTCGA TAAGCAGCAT
4441 CGCCTGTTTC AGGCTGTCTA TGTGTGACTG TTGAGCTGTA ACAAGTTGTC TCAGGTGTTC
4501 AATTTCATGT TCTAGTTGCT TTGTTTTACT GGTTTCACCT GTTCTATTAG GTGTTACATG
4561 CTGTTCATCT GTTACATTGT CGATCTGTTC ATGGTGAACA GCTTTgAATG CACCAAAAAC
4621 TCGTAAAAGC TCTGATGTAT CTATCTTTTT TACACCGTTT TCATCTGTGc atgcGCCGAT
4681 CAACGTCTCA TTTTCGCCAG ATATCGGcgc gccgcttcgT AGCATTTTTA TCCATAAGAT
4741 TAGCGGATCC TACCTGACGC TTTTTATCGC AACTCTCTAC TGTTTCTCCA TGGGAGACCA
4801 CAACGGTTTC CCTCTAGAAA TAATTTTGTT TAACTTTAAG AAGGAGGTCA TACCCGTTTT
4861 TTTGGAAGGA GGTAAATTAA TTAATGCACA CTCTTTACGC CCCTGGAGGA TACGACATTA
4921 TGGGATATTT GCGGCAGATT AGGAACCGCC CAAACCCTCA GGTCGAACTG GGGCCTGTGG
4981 ACACGTCATG TGCCCTGATC CTGTGCGATC TGAAGCAAAA GGACACTCCG ATCGTCTACG
5041 CCTCGGAAGC CTTCTTGTAT ATGACCGGAT ACAGCAATGC AGAGGTGCTC GGCAGGAACT
5101 GCAGATTCCT GCAGTCCCCC GACGGGATGG TGAAACCAAA GTCGACTCGC AAATATGTGG
5161 ACTCGAACAC GATCAACACC ATGCGGAAGG CCATCGACCG GAACGCCGAG GTCCAGGTGG
5221 AGGTGGTCAA CTTTAAGAAG AACGGCCAGC GGTTCGTGAA CTTTCTGACC ATGATTCCGG
5281 TCCGGGATGA AACCGGAGAG TACAGATACT CCATGGGATT CCAGTGCGAA ACCGAAggcg
5341 gtTCTggagg tAGTGAAACC GTTCAGGACA TCTACGGGAT TGTTGCTAAG AAAGTCAACG

```
5401 AGATTCTACA AGCAGACGCA ATCAATGGGA CCGATAACGA AGTAGTTACC GTGACCGATG
5461 AGAACACTGG TGAAATCTCT GAGAAAGTCA AGCTGGGCAC TAAGGCACTG GCTGGTCAAT
5521 GGCTGGCTTA CGGTGTTACT CGCAGTGTGA CTAAGaGTTC AGTCATGACG CTGGCTTACG
5581 GGTCCAAAGA GTTCGGCTTC CGTCAACAAG TGCTGGAAGA TACCATTCAG CCAGCTATTG
5641 ATTCCGGCAA GGGTCTGATG TTCACTCAGC CGAATCAGGC TGCTGGATAC ATGGCTAAGC
5701 TGATTTGGGA ATCTGTGAGC GTGACGGTGG TAGCTGCGGT TGAAGCAATG AACTGGCTTA
5761 AGTCTGCTGC TAAGCTGCTG GCTGCTGAGG TCAAAGATAA GAAGACTGGA GAGATTCTTC
5821 GCAAGCGTTG CGCTGTGCAT TGGGTAACTC CTGATGGTTT CCCTGTGTGG CAGGAATACA
5881 AGAAGCCTAT TCAGACGCGC TTGAACCTGA TGTTCCTCGG TCAGTTCCGC TTACAGCCTA
5941 CCATTAACAC CAACAAAGAT AGCGAGATTG ATGCACACAA ACAGGAGTCT GGTATCGCTC
6001 CTAACTTTGT ACACAGCCAA GACGGTAGCC ACCTTCGTAA GACTGTAGTG TGGGCACACG
6061 AGAAGTACGG AATCGAATCT TTTGCACTGA TTCACGACTC CTTCGGTACC ATTCCGGCTG
6121 ACGCTGCGAA CCTGTTCAAA GCAGTGCGCG AAACTATGGT TGACACATAT GAGTCTTGTG
6181 ATGTACTGGC TGATTTCTAC GACCAGTTCG CTGACCAGTT GCACGAGTCT CAATTGGACA
6241 AAATGCCAGC ACTTCCGGCT AAAGGTAACT TGAACCTCCG TGACATCTTA GAGTCGGACT
6301 TCGCGTTCGC GTAATCTAGA GGCATCAAAT AAAACGAAAG GCTCAGTCGA AAGACTGGGC
6361 CTTTCGTTTT ATCTGTTGTT TGTCGGTGAA CGCTCTCCTG AGTAGGACAA ATCCGCCGCC
6421 CTAGACCTAG CTGCAGCTAG GGTACGGGTT TTGCTGCCCG CAAACGGGCT GTTCTGGTGT
6481 TGCTAGgatc CATATGGACA GTTTTCCCTT TGATATgTAA CGGTGAACAG TTGTTCTACT
6541 TTTGTTTGTT AGTCTTGATG CTTCACTGAT AGATACAAGA GCCATAAGAA CCTCAGATCC
6601 TTCCGTATTT AGCCAGTATG TTCTCTAGTG TGGTTCGTTG TTTTTGCGTG AGCCATGAGA
6661 ACGAACCATT GAGATCATaC TTACTTTGCA TGTCACTCAA AAATTTTGCC TCAAAACTGG
6721 TGAGCTGAAT TTTTGCAGTT AAAGCATCGT GTAGTGTTTT TCTTAGTCCG TTAtGTAGGT
6781 AGGAATCTGA TGTAATGGTT GTTGGTATTT TGTCACCATT CATTTTTATC TGGTTGTTCT
6841 CAAGTTCGGT TACGAGATCC ATTTGTCTAT CTAGTTCAAC TTGGAAAATC AACGTATCAG
6901 TCGGGCGGCC TCGCTTATCA ACCACCAATT TCATATTGCT GTAAGTGTTT AAATCTTTAC
6961 TTATTGGTTT CAAAACCCAT TGGTTAAGCC TTTTAAACTC ATGGTAGTTA TTTTCAAGCA
7021 TTAACATGAA CTTAAATTCA TCAAGGCTAA TCTCTATATT TGCCTTGTGA GTTTTCTTTT
7081 GTGTTAGTTC TTTTAATAAC CACTCATAAA TCCTCATAGA GTATTTGTTT TCAAAAGACT
7141 TAACATGTTC CAGATTATAT TTTATGAATT TTTTTAACTG GAAAAGATAA GGCAATATCT
7201 CTTCACTAAA AACTAATTCT AATTTTTCGC TTGAGAACTT GGCATAGTTT GTCCACTGGA
7261 AAATCTCAAA GCCTTTAACC AAAGGATTCC TGATTTCCAC AGTTCTCGTC ATCAGCTCTC
7321 TGGTTGCTTT AGCTAATACA CCATAAGCAT TTTCCCTACT GATGTTCATC ATCTGAGCGT
7381 ATTGGTTATA AGTGAACGAT ACCGTCCGTT CTTTCCTTGT AGGGTTTTCA ATCGTGGGGT
7441 TGAGTAGTGC CACACAGCAT AAAATTAGCT TGGTTTCATG CTCCGTTAAG TCATAGCGAC
7501 TAATCGCTAG TTCATTTGCT TTGAAAACAA CTAATTCAGA CATACATCTC AATTGGTCT
//
```

```
LOCUS       mJAG063                 3223 bp ds-DNA     circular     28-JUL-2022
DEFINITION  .
FEATURES             Location/Qualifiers
     terminator      60..165
                     /label="lambda T0 terminator"
     CDS             complement(173..964)
                     /label="specR"
     promoter        1119..1136
                     /label="T7_promoter (-17 to +1)"
     CDS             1180..1835
                     /label="CmR"
     misc_feature    1840..1898
                     /label="Terminator L3S2P21"
     terminator      1917..2014
                     /label="rrnB T1 terminator"
     rep_origin      2481..3168
                     /label="p15A ori"
ORIGIN
        1 ATTTATCTCT TCAAATGTAG CACCTGAAGT CAGCCCCATA CGATATAAGT TGTTACTAGT
       61 GCTTGGATTC TCACCAATAA AAAACGCCCG GCGGCAACCG AGCGTTCTGA ACAAATCCAG
      121 ATGGAGTTCT GAGGTCATTA CTGGATCTAT CAACAGGAGT CCAAGCGAGC TCTTATTTGC
      181 CGACTACCTT GGTGATCTCG CCTTTCACGT AGTGGACAAA TTCTTCCAAC TGATCTGCGC
      241 GCGAGGCCAA GCGATCTTCT TCTTGTCCAA GATAAGCCTG TCTAGCTTCA AGTATGACGG
      301 GCTGATACTG GGCCGGCAGG CGCTCCATTG CCCAGTCGGC AGCGACATCC TTCGGCGCGA
      361 TTTTGCCGGT TACTGCGCTG TACCAAATGC GGGACAACGT AAGCACTACA TTTCGCTCAT
      421 CGCCAGCCCA GTCGGGCGGC GAGTTCCATA GCGTTAAGGT TTCATTTAGC GCCTCAAATA
      481 GATCCTGTTC AGGAACCGGA TCAAAGAGTT CCTCCGCCGC TGGACCTACC AAGGCAACGC
      541 TATGTTCTCT TGCTTTTGTC AGCAAGATAG CCAGATCAAT GTCGATCGTG GCTGGCTCGA
      601 AGATACCTGC AAGAATGTCA TTGCGCTGCC ATTCTCCAAA TTGCAGTTCG CGCTTAGCTG
      661 GATAACGCCA CGGAATGATG TCGTCGTGCA CAACAATGGT GACTTCTACA GCGCGGAGAA
      721 TCTCGCTCTC TCCAGGGGAA GCCGAAGTTT CCAAAAGGTC GTTGATCAAA GCTCGCCGCG
      781 TTGTTTCATC AAGCCTTACG GTCACCGTAA CCAGCAAATC AATATCACTG TGTGGCTTCA
      841 GGCCGCCATC CACTGCGGAG CCGTACAAAT GTACGGCCAG CAACGTCGGT TCGAGATGGC
      901 GCTCGATGAC GCCAACTACC TCTGATAGTT GAGTCGATAC TTCGGCGATC ACCGCTTCCC
      961 TCATGCGAAA CGATCCTCAT CCTGTCTCTT GATCAGATAT TGATCCCCTG CGCCATCAGA
     1021 TCCTTGGCGG CAAGAAAGCC ATCCAGTTTA CTTTGCAGGG CTTCCCAACC TTACCAGAGG
     1081 GCGCCCCAGC TGGCAATTCC GACGTCGcgg ccgcAGGGTA ATACGACTCA CTATAGGGAG
     1141 AGGATCCTGT CAATTTCCGC GATAGAGGAG GTAagatcAA TGGAGAAAAA AATCACTGGA
     1201 TATACCACCG TTGATATATC CCAATGGCAT CGTAAAGAAC ATTTTGAGGC ATTTCAGTCA
     1261 GTTGCTCAAT GTACCTATAA CCAGACCGTT CAGCTGGATA TTACGGCCTT TTTAAAGACC
     1321 GTAAAGAAAA ATAAGCACAA GTTTTATCCG GCCTTTATTC ACATTCTTGC CCGCCTGATG
     1381 AATGCTCATC CGGAATTTCG TATGGCAATG AAAGACGGTG AGCTGGTGAT ATGGGATAGT
     1441 GTTCACCCTT GTTACACCGT TTTCCATGAG CAAACTGAAA CGTTTTCATC GCTCTGGAGT
     1501 GAATACCACG ACGATTTCCG GCAGTTTCTA CACATATATT CGCAAGATGT GGCGTGTTAt
     1561 GGTGAAAACC TGGCCTATTT CCCTAAAGGG TTTATTGAGA ATATGTTTTT CGTCTCAGCC
     1621 AATCCCTGGG TGAGTTTCAC CAGTTTTGAT TTAAACGTGG CCAATATGGA CAACTTCTTC
     1681 GCCCCCGTTT TCACCATGGG CAAATATTAT ACGCAAGGCG ACAAGGTGCT GATGCCGCTG
     1741 GCGATTCAGG TTCATCATGC CGTtTGTGAT GGCTTCCATG TCGGCAGAAT GCTTAATGAA
     1801 TTACAACAGT ACTGCGATGA GTGGCAGGGC GGGGCGTAAC TCGGTACCAA ATTCCAGAAA
     1861 AGAGGCCTCC CGAAAGGGGG GCCTTTTTTC GTTTTGGTCC AAgcggccgc CCTAGGAGGC
     1921 ATCAAATAAA ACGAAAGGCT CAGTCGAAAG ACTGGGCCTT TCGTTTTATC TGTTGTTTGT
     1981 CGGTGAACGC TCTCCTGAGT AGGACAAATC CGCCGCCCTA GACCTAGCTG CAGCTAGGGT
     2041 ACGGGTTTTG CTGCCCGCAA ACGGGCTGTT CTGGTGTTGC TAGTTTGTTA TCAGAATCGC
     2101 AGATCCGGCT TCAGccgGTT TGCCGGCTGA AAGCGCTATT TCTTCCAGAA TTGCCATGAT
     2161 TTTTTCCCCA CGGGAGGCGT CACTGGCTCC CGTGTTGTCG GCAGCTTTGA TTCGATAAGC
     2221 AGCATCGCCT GTTTCAGGCT GTCTATGTGT GACTGTTGAG CTGTAACAAG TTGTCTCAGG
     2281 TGTTCAATTT CATGTTCTAG TTGCTTTGTT TTACTGGTTT CACCTGTTCT ATTAGGTGTT
     2341 ACATGCTGTT CATCTGTTAC ATTGTCGATC TGTTCATGGT GAACAGCTTT gAATGCACCA
     2401 AAAACTCGTA AAAGCTCTGA TGTATCTATC TTTTTTACAC CGTTTTCATC TGTGcatgcA
     2461 cgaagcggcg cgCCCATATG GGATATATTC CGCTTCCTCG CTCACTGACT CGCTACGCTC
     2521 GGTCGTTCGA CTGCGGCGAG CGGAAATGGC TTACGAACGG GGCGGAGATT TCCTGGAAGA
     2581 TGCCAGGAAG ATACTTAACA GGGAAGTGAG AGGGCCGCGG CAAAGCCGTT TTTCCATAGG
```

```
     2641 CTCCGCCCCC CTGACAAGCA TCACGAAATC TGACGCTCAA ATCAGTGGTG GCGAAACCCG
     2701 ACAGGACTAT AAAGATACCA GGCGTTTCCC CCTGGCGGCT CCCTCGTGCG CTCTCCTGTT
     2761 CCTGCCTTTC GGTTTACCGG TGTCATTCCG CTGTTATGGC CGCGTTTGTC TCATTCCACG
     2821 CCTGACACTC AGTTCCGGGT AGGCAGTTCG CTCCAAGCTG GACTGTATGC ACGAACCCCC
     2881 CGTTCAGTCC GACCGCTGCG CCTTATCCGG TAACTATCGT CTTGAGTCCA ACCCGGAAAG
     2941 ACATGCAAAA GCACCACTGG CAGCAGCCAC TGGTAATTGA TTTAGAGGAG TTAGTCTTGA
     3001 AGTCATGCGC CGGTTAAGGC TAAACTGAAA GGACAAGTTT TGGTGACTGC GCTCCTCCAA
     3061 GCCAGTTACC TCGGTTCAAA GAGTTGGTAG CTCAGAGAAC CTTCGAAAAA CCGCCCTGCA
     3121 AGGCGGTTTT TTCGTTTTCA GAGCAAGAGA TTACGCGCAG ACCAAAACGA TCTCAAGAAG
     3181 ATCATCTTAT TAATCAGATA AAATATTACT AGATTTCAGT GCA
//
```

```
LOCUS       mJAG090                 3172 bp ds-DNA     circular     28-JUL-2022
DEFINITION  .
FEATURES             Location/Qualifiers
     terminator           complement(53..147)
                          /label="lambda t0 terminator"
     Antibiotic Resistance complement(177..1037)
                          /label="AmpR"
     terminator           1186..1351
                          /label="rrnBT1T2 terminator - truncated"
     misc_feature         1356..1399
                          /label="J23101 mutant (Weak Constitutive Promoter)"
     ribozyme             1416..1493
                          /label="AraJ  (Insulator Ribozyme)"
     RBS                  1498..1517
                          /label="B0033m (RBS)"
     CDS                  1522..2199
                          /label="mcherry226"
     misc_feature         2206..2266
                          /label="Terminator L3S2P21 (synthetic, strong)"
     terminator           2282..2379
                          /label="rrnB T1 terminator"
     rep_origin           complement(2517..3160)
                          /label="colE1 ori"
ORIGIN
        1 TCAGTGGAAC GAAAACTCAC GTTAAGGGAT TTTGGTCATG ACTAGTGCTT GGATTCTCAC
       61 CAATAAAAAA CGCCCGGCGG CAACCGAGCG TTCTGAACAA ATCCAGATGG AGTTCTGAGG
      121 TCATTACTGG ATCTATCAAC AGGAGTCCAA GCGAGCTCGT AAACTTGGTC TGACAGTTAC
      181 CAATGCTTAA TCAGTGAGGC ACCTATCTCA GCGATCTGTC TATTTCGTTC ATCCATAGTT
      241 GCCTGACTCC CCGTCGTGTA GATAACTACG ATACGGGAGG GCTTACCATC TGGCCCCAGT
      301 GCTGCAATGA TACCGCGcga CCCACGCTCA CCGGCTCCAG ATTTATCAGC AATAAACCAG
      361 CCAGCCGGAA GGGCCGAGCG CAGAAGTGGT CCTGCAACTT TATCCGCCTC CATCCAGTCT
      421 ATTAATTGTT GCCGGGAAGC TAGAGTAAGT AGTTCGCCAG TTAATAGTTT GCGCAACGTT
      481 GTTGCCATTG CTACAGGCAT CGTGGTGTCA CGCTCGTCGT TTGGTATGGC TTCATTCAGC
      541 TCCGGTTCCC AACGATCAAG GCGAGTTACA TGATCCCCCA TGTTGTGCAA AAAAGCGGTT
      601 AGCTCCTTCG GTCCTCCGAT CGTTGTCAGA AGTAAGTTGG CCGCAGTGTT ATCACTCATG
      661 GTTATGGCAG CACTGCATAA TTCTCTTACT GTCATGCCAT CCGTAAGATG CTTTTCTGTG
      721 ACTGGTGAGT ACTCAACCAA GTCATTCTGA GAATAGTGTA TGCGGCGACC GAGTTGCTCT
      781 TGCCCGGCGT CAATACGGGA TAATACCGCG CCACATAGCA GAACTTTAAA AGTGCTCATC
      841 ATTGGAAAAC GTTCTTCGGG GCGAAAACTC TCAAGGATCT TACCGCTGTT GAGATCCAGT
      901 TCGATGTAAC CCACTCGTGC ACCCAACTGA TCTTCAGCAT CTTTTACTTT CACCAGCGTT
      961 TCTGGGTGAG CAAAAACAGG AAGGCAAAAT GCCGCAAAAA AGGGAATAAG GGCGACACGG
     1021 AAATGTTGAA TACTCATACT CTTCCTTTTT CAATATTATT GAAGCATTTA TCAGGGTTAT
     1081 TGTCTCATGA GCGGATACAT ATTTGAATGT ATTTAGAAAA ATAAACAAAT AGGGGTTCCG
     1141 CGCACATTTC CCCGAAAAGT GCCACCTGAC GTCGcggccg cAGGGGGCTG TTTTGGCGGA
     1201 TGAGAGAAGA TTTTCAGCCT GATACAGATT AAATCAGAAC GCAGAAGCGG TCTGATAAAA
     1261 CAGAATTTGC CTGGCGGCAG TAGCGCGGTG GTCCCACCTG ACCCCATGCC GAACTCAGAA
     1321 GTGAAACGCC GTAGCGCCGA TGGTAGTGTG GAGCAcattc tagagtttac agctagctca
     1381 gtcctaggta tatgctagcA TAGGTGTGGT CATAGAGTGG TCGTGATCTG AAACTCGATC
     1441 ACCTGATGAG CTCAAGGCAG AGCGAAACCA CCTCTACAAA TAATTTTGTT TAATACTaga
     1501 gtcacacagg actactaAAT GGCTATCATT AAAGAGTTCA TGCGCTTCAA AGTTCACATG
     1561 GAGGGTTCTG TTAACGGTCA CGAGTTCGAG ATCGAAGGCG AAGGCGAGGG CCGTCCGTAT
     1621 GAAGGCACCC AGACCGCCAA ACTGAAAGTG ACTAAAGGCG GCCCGCTGCC TTTTGCGTGG
     1681 GACATCCTGA GCCCGCAATT TATGTACGGT TCTAAAGCGT ATGTTAAACA CCCAGCGGAT
     1741 ATCCCGGACT ATCTGAAGCT GTCTTTTCCG GAAGGTTTCA AGTGGGAACG CGTAATGAAT
     1801 TTTGAAGATG GTGGTGTCGT GACCGTCACT CAGGACTCCT CCCTGCAGGA TGGCGAGTTC
     1861 ATCTATAAAG TTAAACTGCG TGGTACTAAT TTTCCATCTG ATGGCCCGGT GATGCAGAAA
     1921 AAGACGATGG GTTGGGAGGC GTCTAGCGAA CGCATGTATC CGGAAGATGG TGCGCTGAAA
     1981 GGCGAAATTA AACAGCGCCT GAAACTGAAA GATGGCGGCC ATTATGACGC TGAAGTGAAA
     2041 ACCACGTACA AAGCCAAGAA ACCTGTGCAG CTGCCTGGCG CGTACAATGT GAATATTAAA
     2101 CTGGACATCA CCTCTCATAA TGAAGATTAT ACGATCGTAG AGCAATATGA GCGCGCGGAG
     2161 GGTCGTCATT CTACCGGTGG CATGGATGAG CTGTACAAAT AGTAACTCGG TACCAAATTC
     2221 CAGAAAAGAG GCCTCCCGAA AGGGGGGCCT TTTTTCGTTT TGGTCCAAgc ggccgcTCTA
```

```
2281 GAGGCATCAA ATAAAACGAA AGGCTCAGTC GAAAGACTGG GCCTTTCGTT TTATCTGTTG
2341 TTTGTCGGTG AACGCTCTCC TGAGTAGGAC AAATCCGCCG CCCTAGACCT AGCTGCAGCT
2401 AGGGCGTTCG GCTGCGGCGA GCGGTATCAG CTCACTCAAA GGCGGTAATA CGGTTATCCA
2461 CAGAATCAGG GGATAACGCA GGAAAGAACA TGTGAGCAAA AGGCCAGCAA AAGGCCAGGA
2521 ACCGTAAAAA GGCCGCGTTG CTGGCGTTTT TCCATAGGCT CCGCCCCCCT GACGAGCATC
2581 ACAAAAATCG ACGCTCAAGT CAGAGGTGGC GAAACCCGAC AGGACTATAA AGATACCAGG
2641 CGTTTCCCCC TGGAAGCTCC CTCGTGCGCT CTCCTGTTCC GACCCTGCCG CTTACCGGAT
2701 ACCTGTCCGC CTTTCTCCCT TCGGGAAGCG TGGCGCTTTC TCAtaGCTCA CGCTGTAGGT
2761 ATCTCAGTTC GGTGTAGGTC GTTCGCTCCA AGCTGGGCTG TGTGCACGAA CCCCCCGTTC
2821 AGCCCGACCG CTGCGCCTTA TCCGGTAACT ATCGTCTTGA GTCCAACCCG GTAAGACACG
2881 ACTTATCGCC ACTGGCAGCA GCCACTGGTA ACAGGATTAG CAGAGCGAGG TATGTAGGCG
2941 GTGCTACAGA GTTCTTGAAG TGGTGGCCTA ACTACGGCTA CACTAGAAGG ACAGTATTTG
3001 GTATCTGCGC TCTGCTGAAG CCAGTTACCT TCGGAAAAAG AGTTGGTAGC TCTTGATCCG
3061 GCAAACAAAC CACCGCTGGT AGCGGTGGTT TTTTTGTTTG CAAGCAGCAG ATTACGCGCA
3121 GAAAAAAAGG ATCTCAAGAA GATCCTTTGA TCTTTTCTAC GGGGTCTGAC GC
//
```

```
LOCUS       mJAG099                4224 bp ds-DNA     circular     28-JUL-2022
DEFINITION  .
FEATURES             Location/Qualifiers
     rep_origin      complement(219..580)
                     /label="pSC101 ori"
     terminator      complement(597..691)
                     /label="lambda t0 terminator"
     CDS             complement(703..1494)
                     /label="specR"
     misc_feature    1657..1690
                     /label="J23101 mutant (Weak Constitutive Promoter)"
     RBS             1710..1723
                     /label="BBa_B0031 (RBS)"
     CDS             1725..2384
                     /label="CmR"
     misc_feature    2385..2445
                     /label="Terminator L3S2P21 (synthetic, strong)"
     terminator      2469..2566
                     /label="rrnB T1 terminator"
     CDS             complement(3112..4059)
                     /label="repA"
ORIGIN
        1 ACCTTTGTGT GTTTTTTTTG TTTATATTCA AGTGGTTATA ATTTATAGAA TAAAGAAAGA
       61 ATAAAAAAAG ATAAAAAGAA TAGATCCCAG CCCTGTGTAT AACTCACTAC TTTAGTCAGT
      121 TCCGCAGTAT TACAAAAGGA TGTCGCAAAC GCTGTTTGCT CCTCTACAAA ACAGACCTTA
      181 AAACCCTAAA GGCTTAAGTA GCACCCTCGC AAGCTCGGGC AAATCGCTGA ATATTCCTTT
      241 TGTCTCCGAC CATCAGGCAC CTGAGTCGCT GTCTTTTTCG TGACATTCAG TTCGCTGCGC
      301 TCACGGCTCT GGCAGTGAAT GGGGGTAAAT GGCACTACAG GCGCCTTTTA TGGATTCATG
      361 CAAGGAAACT ACCCATAATA CAAGAAAAGC CCGTCACGGG CTTCTCAGGG CGTTTTATGG
      421 CGGGTCTGCT ATGTGGTGCT ATCTGACTTT TTGCTGTTCA GCAGTTCCTG CCCTCTGATT
      481 TTCCAGTCTG ACCACTTCGG ATTATCCCGT GACAGGTCAT TCAGACTGGC TAATGCACCC
      541 AGTAAGGCAG CGGTATCATC AACAGGCTTA CCCGTCTTAC TGTCCCTAGT GCTTGGATTC
      601 TCACCAATAA AAAACGCCCG GCGGCAACCG AGCGTTCTGA ACAAATCCAG ATGGAGTTCT
      661 GAGGTCATTA CTGGATCTAT CAACAGGAGT CCAAGCGAGC TCTTATTTGC CGACTACCTT
      721 GGTGATCTCG CCTTTCACGT AGTGGACAAA TTCTTCCAAC TGATCTGCGC GCGAGGCCAA
      781 GCGATCTTCT TCTTGTCCAA GATAAGCCTG TCTAGCTTCA AGTATGACGG GCTGATACTG
      841 GGCCGGCAGG CGCTCCATTG CCCAGTCGGC AGCGACATCC TTCGGCGCGA TTTTGCCGGT
      901 TACTGCGCTG TACCAAATGC GGGACAACGT AAGCACTACA TTTCGCTCAT CGCCAGCCCA
      961 GTCGGGCGGC GAGTTCCATA GCGTTAAGGT TTCATTTAGC GCCTCAAATA GATCCTGTTC
     1021 AGGAACCGGA TCAAAGAGTT CCTCCGCCGC TGGACCTACC AAGGCAACGC TATGTTCTCT
     1081 TGCTTTTGTC AGCAAGATAG CCAGATCAAT GTCGATCGTG GCTGGCTCGA AGATACCTGC
     1141 AAGAATGTCA TTGCGCTGCC ATTCTCCAAA TTGCAGTTCG CGCTTAGCTG GATAACGCCA
     1201 CGGAATGATG TCGTCGTGCA CAACAATGGT GACTTCTACA GCGCGGAGAA TCTCGCTCTC
     1261 TCCAGGGGAA GCCGAAGTTT CCAAAAGGTC GTTGATCAAA GCTCGCCGCG TTGTTTCATC
     1321 AAGCCTTACG GTCACCGTAA CCAGCAAATC AATATCACTG TGTGGCTTCA GGCCGCCATC
     1381 CACTGCGGAG CCGTACAAAT GTACGGCCAG CAACGTCGGT TCGAGATGGC GCTCGATGAC
     1441 GCCAACTACC TCTGATAGTT GAGTCGATAC TTCGGCGATC ACCGCTTCCC TCATGCGAAA
     1501 CGATCCTCAT CCTGTCTCTT GATCAGATAT TGATCCCCTG CGCCATCAGA TCCTTGGCGG
     1561 CAAGAAAGCC ATCCAGTTTA CTTTGCAGGG CTTCCCAACC TTACCAGAGG GCGCCCCAGC
     1621 TGGCAATTCC GACGTCgcgg ccgcgaaGAC ATAGGGttta cagctagctc agtcctaggt
     1681 atatgctagc AGCGATAACA AACTTTGACT CACACAGGAA ACCAATGGAG AAAAAAATCA
     1741 CTGGATATAC CACCGTTGAT ATATCCCAAT GGCATCGTAA AGAACATTTT GAGGCATTTC
     1801 AGTCAGTTGC TCAATGTACC TATAACCAGA CCGTTCAGCT GGATATTACG GCCTTTTTAA
     1861 AGACCGTAAA GAAAAATAAG CACAAGTTTT ATCCGGCCTT TATTCACATT CTTGCCCGCC
     1921 TGATGAATGC TCATCCGGAA TTTCGTATGG CAATGAAAGA CGGTGAGCTG GTGATATGGG
     1981 ATAGTGTTCA CCCTTGTTAC ACCGTTTTCC ATGAGCAAAC TGAAACGTTT TCATCGCTCT
     2041 GGAGTGAATA CCACGACGAT TTCCGGCAGT TTCTACACAT ATATTCGCAA GATGTGGCGT
     2101 GTTAtGGTGA AAACCTGGCC TATTTCCCTA AAGGGTTTAT TGAGAATATG TTTTTCGTCT
     2161 CAGCCAATCC CTGGGTGAGT TTCACCAGTT TTGATTTAAA CGTGGCCAAT ATGGACAACT
     2221 TCTTCGCCCC CGTTTTCACC ATGGGCAAAT ATTATACGCA AGGCGACAAG GTGCTGATGC
     2281 CGCTGGCGAT TCAGGTTCAT CATGCCGTtT GTGATGGCTT CCATGTCGGC AGAATGCTTA
     2341 ATGAATTACA ACAGTACTGC GATGAGTGGC AGGGCGGGGC GTAACTCGGT ACCAAATTCC
```

```
2401 AGAAAAGAGG CCTCCCGAAA GGGGGGCCTT TTTTCGTTTT GGTCCAAATG TCTTCgcggc
2461 cgcTCTAGAG GCATCAAATA AAACGAAAGG CTCAGTCGAA AGACTGGGCC TTTCGTTTTA
2521 TCTGTTGTTT GTCGGTGAAC GCTCTCCTGA GTAGGACAAA TCCGCCGCCC TAGACCTAGC
2581 TGCAGCTAGG GTACGGGTTT TGCTGCCCGC AAACGGGCTG TTCTGGTGTT GCTAGTTTGT
2641 TATCAGAATC GCAGATCCGG CTTCAGccgG TTTGCCGGCT GAAAGCGCTA TTTCTTCCAG
2701 AATTGCCATG ATTTTTTCCC CACGGGAGGC GTCACTGGCT CCCGTGTTGT CGGCAGCTTT
2761 GATTCGATAA GCAGCATCGC CTGTTTCAGG CTGTCTATGT GTGACTGTTG AGCTGTAACA
2821 AGTTGTCTCA GGTGTTCAAT TTCATGTTCT AGTTGCTTTG TTTTACTGGT TTCACCTGTT
2881 CTATTAGGTG TTACATGCTG TTCATCTGTT ACATTGTCGA TCTGTTCATG GTGAACAGCT
2941 TTgAATGCAC CAAAAACTCG TAAAAGCTCT GATGTATCTA TCTTTTTTAC ACCGTTTTCA
3001 TCTGTGCATA TGGACAGTTT TCCCTTTGAT ATgTAACGGT GAACAGTTGT TCTACTTTTG
3061 TTTGTTAGTC TTGATGCTTC ACTGATAGAT ACAAGAGCCA TAAGAACCTC AGATCCTTCC
3121 GTATTTAGCC AGTATGTTCT CTAGTGTGGT TCGTTGTTTT TGCGTGAGCC ATGAGAACGA
3181 ACCATTGAGA TCATaCTTAC TTTGCATGTC ACTCAAAAAT TTTGCCTCAA AACTGGTGAG
3241 CTGAATTTTT GCAGTTAAAG CATCGTGTAG TGTTTTTCTT AGTCCGTTAt GTAGGTAGGA
3301 ATCTGATGTA ATGGTTGTTG GTATTTTGTC ACCATTCATT TTTATCTGGT TGTTCTCAAG
3361 TTCGGTTACG AGATCCATTT GTCTATCTAG TTCAACTTGG AAAATCAACG TATCAGTCGG
3421 GCGGCCTCGC TTATCAACCA CCAATTTCAT ATTGCTGTAA GTGTTTAAAT CTTTACTTAT
3481 TGGTTTCAAA ACCCATTGGT TAAGCCTTTT AAACTCATGG TAGTTATTTT CAAGCATTAA
3541 CATGAACTTA AATTCATCAA GGCTAATCTC TATATTTGCC TTGTGAGTTT TCTTTTGTGT
3601 TAGTTCTTTT AATAACCACT CATAAATCCT CATAGAGTAT TTGTTTTCAA AAGACTTAAC
3661 ATGTTCCAGA TTATATTTTA TGAATTTTTT TAACTGGAAA AGATAAGGCA ATATCTCTTC
3721 ACTAAAAACT AATTCTAATT TTTCGCTTGA GAACTTGGCA TAGTTTGTCC ACTGGAAAAT
3781 CTCAAAGCCT TTAACCAAAG GATTCCTGAT TTCCACAGTT CTCGTCATCA GCTCTCTGGT
3841 TGCTTTAGCT AATACACCAT AAGCATTTTC CCTACTGATG TTCATCATCT GAGCGTATTG
3901 GTTATAAGTG AACGATACCG TCCGTTCTTT CCTTGTAGGG TTTTCAATCG TGGGGTTGAG
3961 TAGTGCCACA CAGCATAAAA TTAGCTTGGT TTCATGCTCC GTTAAGTCAT AGCGACTAAT
4021 CGCTAGTTCA TTTGCTTTGA AAACAACTAA TTCAGACATA CATCTCAATT GGTCTAGGTG
4081 ATTTTAATCA CTATACCAAT TGAGATGGGC TAGTCAATGA TAATTACTAG TCCTTTTCCT
4141 TTGAGTTGTG GGTATCTGTA AATTCTGCTA GACCTTTGCT GGAAAACTTG TAAATTCTGC
4201 TAGACCCTCT GTAAATTCCG CTAG
//
```

```
LOCUS       mJAG100                4260 bp ds-DNA     circular     28-JUL-2022
DEFINITION  .
FEATURES             Location/Qualifiers
     terminator      complement(10..104)
                     /label="lambda t0 terminator"
     CDS             complement(116..907)
                     /label="specR"
     misc_feature    1070..1103
                     /label="J23101 mutant (Weak Constitutive Promoter)"
     RBS             1123..1136
                     /label="BBa_B0031 (RBS)"
     misc_feature    1138..1794
                     /label="CmR"
     Degradation Tag 1795..1827
                     /label="LAA-ssrA tag"
     misc_feature    1834..1894
                     /label="Terminator L3S2P21 (synthetic, strong)"
     terminator      1918..2015
                     /label="rrnB T1 terminator"
     CDS             complement(2561..3508)
                     /label="repA"
     rep_origin      complement(3892..4253)
                     /label="pSC101 ori"
ORIGIN
        1 AGTGCTTGGA TTCTCACCAA TAAAAAACGC CCGGCGGCAA CCGAGCGTTC TGAACAAATC
       61 CAGATGGAGT TCTGAGGTCA TTACTGGATC TATCAACAGG AGTCCAAGCG AGCTCTTATT
      121 TGCCGACTAC CTTGGTGATC TCGCCTTTCA CGTAGTGGAC AAATTCTTCC AACTGATCTG
      181 CGCGCGAGGC CAAGCGATCT TCTTCTTGTC CAAGATAAGC CTGTCTAGCT TCAAGTATGA
      241 CGGGCTGATA CTGGGCCGGC AGGCGCTCCA TTGCCCAGTC GGCAGCGACA TCCTTCGGCG
      301 CGATTTTGCC GGTTACTGCG CTGTACCAAA TGCGGGACAA CGTAAGCACT ACATTTCGCT
      361 CATCGCCAGC CCAGTCGGGC GGCGAGTTCC ATAGCGTTAA GGTTTCATTT AGCGCCTCAA
      421 ATAGATCCTG TTCAGGAACC GGATCAAAGA GTTCCTCCGC CGCTGGACCT ACCAAGGCAA
      481 CGCTATGTTC TCTTGCTTTT GTCAGCAAGA TAGCCAGATC AATGTCGATC GTGGCTGGCT
      541 CGAAGATACC TGCAAGAATG TCATTGCGCT GCCATTCTCC AAATTGCAGT TCGCGCTTAG
      601 CTGGATAACG CCACGGAATG ATGTCGTCGT GCACAACAAT GGTGACTTCT ACAGCGCGGA
      661 GAATCTCGCT CTCTCCAGGG GAAGCCGAAG TTTCCAAAAG GTCGTTGATC AAAGCTCGCC
      721 GCGTTGTTTC ATCAAGCCTT ACGGTCACCG TAACCAGCAA ATCAATATCA CTGTGTGGCT
      781 TCAGGCCGCC ATCCACTGCG GAGCCGTACA AATGTACGGC CAGCAACGTC GGTTCGAGAT
      841 GGCGCTCGAT GACGCCAACT ACCTCTGATA GTTGAGTCGA TACTTCGGCG ATCACCGCTT
      901 CCCTCATGCG AAACGATCCT CATCCTGTCT CTTGATCAGA TATTGATCCC CTGCGCCATC
      961 AGATCCTTGG CGGCAAGAAA GCCATCCAGT TTACTTTGCA GGGCTTCCCA ACCTTACCAG
     1021 AGGGCGCCCC AGCTGGCAAT TCCGACGTCg cggccgcgaa GACATAGGGt ttacagctag
     1081 ctcagtccta ggtatatgct agcAGCGATA ACAAACTTTG ACTCACACAG GAAACCAATG
     1141 GAGAAAAAAA TCACTGGATA TACCACCGTT GATATATCCC AATGGCATCG TAAAGAACAT
     1201 TTTGAGGCAT TTCAGTCAGT TGCTCAATGT ACCTATAACC AGACCGTTCA GCTGGATATT
     1261 ACGGCCTTTT TAAAGACCGT AAAGAAAAAT AAGCACAAGT TTTATCCGGC CTTTATTCAC
     1321 ATTCTTGCCC GCCTGATGAA TGCTCATCCG GAATTTCGTA TGGCAATGAA AGACGGTGAG
     1381 CTGGTGATAT GGGATAGTGT TCACCCTTGT TACACCGTTT TCCATGAGCA AACTGAAACG
     1441 TTTTCATCGC TCTGGAGTGA ATACCACGAC GATTTCCGGC AGTTTCTACA CATATATTCG
     1501 CAAGATGTGG CGTGTTAtGG TGAAAACCTG GCCTATTTCC CTAAAGGGTT TATTGAGAAT
     1561 ATGTTTTTCG TCTCAGCCAA TCCCTGGGTG AGTTTCACCA GTTTTGATTT AAACGTGGCC
     1621 AATATGGACA ACTTCTTCGC CCCCGTTTTC ACCATGGGCA AATATTATAC GCAAGGCGAC
     1681 AAGGTGCTGA TGCCGCTGGC GATTCAGGTT CATCATGCCG TtTGTGATGG CTTCCATGTC
     1741 GGCAGAATGC TTAATGAATT ACAACAGTAC TGCGATGAGT GGCAGGGCGG GGCgGCTGct
     1801 aacgacgaaa actacgctct ggctgctTAG TAACTCGGTA CCAAATTCCA GAAAAGAGGC
     1861 CTCCCGAAAG GGGGGCCTTT TTTCGTTTTG GTCCAAATGT CTTCgcggcc gcTCTAGAGG
     1921 CATCAAATAA AACGAAAGGC TCAGTCGAAA GACTGGGCCT TTCGTTTTAT CTGTTGTTTG
     1981 TCGGTGAACG CTCTCCTGAG TAGGACAAAT CCGCCGCCCT AGACCTAGCT GCAGCTAGGG
     2041 TACGGGTTTT GCTGCCCGCA AACGGGCTGT TCTGGTGTTG CTAGTTTGTT ATCAGAATCG
     2101 CAGATCCGGC TTCAGccgGT TTGCCGGCTG AAAGCGCTAT TTCTTCCAGA ATTGCCATGA
     2161 TTTTTTCCCC ACGGGAGGCG TCACTGGCTC CCGTGTTGTC GGCAGCTTTG ATTCGATAAG
     2221 CAGCATCGCC TGTTTCAGGC TGTCTATGTG TGACTGTTGA GCTGTAACAA GTTGTCTCAG
```

```
2281 GTGTTCAATT TCATGTTCTA GTTGCTTTGT TTTACTGGTT TCACCTGTTC TATTAGGTGT
2341 TACATGCTGT TCATCTGTTA CATTGTCGAT CTGTTCATGG TGAACAGCTT TgAATGCACC
2401 AAAAACTCGT AAAAGCTCTG ATGTATCTAT CTTTTTTACA CCGTTTTCAT CTGTGCATAT
2461 GGACAGTTTT CCCTTTGATA TgTAACGGTG AACAGTTGTT CTACTTTTGT TTGTTAGTCT
2521 TGATGCTTCA CTGATAGATA CAAGAGCCAT AAGAACCTCA GATCCTTCCG TATTTAGCCA
2581 GTATGTTCTC TAGTGTGGTT CGTTGTTTTT GCGTGAGCCA TGAGAACGAA CCATTGAGAT
2641 CATaCTTACT TTGCATGTCA CTCAAAAATT TTGCCTCAAA ACTGGTGAGC TGAATTTTTG
2701 CAGTTAAAGC ATCGTGTAGT GTTTTTCTTA GTCCGTTAtG TAGGTAGGAA TCTGATGTAA
2761 TGGTTGTTGG TATTTTGTCA CCATTCATTT TTATCTGGTT GTTCTCAAGT TCGGTTACGA
2821 GATCCATTTG TCTATCTAGT TCAACTTGGA AAATCAACGT ATCAGTCGGG CGGCCTCGCT
2881 TATCAACCAC CAATTTCATA TTGCTGTAAG TGTTTAAATC TTTACTTATT GGTTTCAAAA
2941 CCCATTGGTT AAGCCTTTTA AACTCATGGT AGTTATTTTC AAGCATTAAC ATGAACTTAA
3001 ATTCATCAAG GCTAATCTCT ATATTTGCCT TGTGAGTTTT CTTTTGTGTT AGTTCTTTTA
3061 ATAACCACTC ATAAATCCTC ATAGAGTATT TGTTTTCAAA AGACTTAACA TGTTCCAGAT
3121 TATATTTTAT GAATTTTTTT AACTGGAAAA GATAAGGCAA TATCTCTTCA CTAAAAACTA
3181 ATTCTAATTT TTCGCTTGAG AACTTGGCAT AGTTTGTCCA CTGGAAAATC TCAAAGCCTT
3241 TAACCAAAGG ATTCCTGATT TCCACAGTTC TCGTCATCAG CTCTCTGGTT GCTTTAGCTA
3301 ATACACCATA AGCATTTTCC CTACTGATGT TCATCATCTG AGCGTATTGG TTATAAGTGA
3361 ACGATACCGT CCGTTCTTTC CTTGTAGGGT TTTCAATCGT GGGGTTGAGT AGTGCCACAC
3421 AGCATAAAAT TAGCTTGGTT TCATGCTCCG TTAAGTCATA GCGACTAATC GCTAGTTCAT
3481 TTGCTTTGAA AACAACTAAT TCAGACATAC ATCTCAATTG GTCTAGGTGA TTTTAATCAC
3541 TATACCAATT GAGATGGGCT AGTCAATGAT AATTACTAGT CCTTTTCCTT TGAGTTGTGG
3601 GTATCTGTAA ATTCTGCTAG ACCTTTGCTG GAAAACTTGT AAATTCTGCT AGACCCTCTG
3661 TAAATTCCGC TAGACCTTTG TGTGTTTTTT TTGTTTATAT TCAAGTGGTT ATAATTTATA
3721 GAATAAAGAA AGAATAAAAA AAGATAAAAA GAATAGATCC CAGCCCTGTG TATAACTCAC
3781 TACTTTAGTC AGTTCCGCAG TATTACAAAA GGATGTCGCA AACGCTGTTT GCTCCTCTAC
3841 AAAACAGACC TTAAAACCCT AAAGGCTTAA GTAGCACCCT CGCAAGCTCG GGCAAATCGC
3901 TGAATATTCC TTTTGTCTCC GACCATCAGG CACCTGAGTC GCTGTCTTTT TCGTGACATT
3961 CAGTTCGCTG CGCTCACGGC TCTGGCAGTG AATGGGGGTA AATGGCACTA CAGGCGCCTT
4021 TTATGGATTC ATGCAAGGAA ACTACCCATA ATACAAGAAA AGCCCGTCAC GGGCTTCTCA
4081 GGGCGTTTTA TGGCGGGTCT GCTATGTGGT GCTATCTGAC TTTTTGCTGT TCAGCAGTTC
4141 CTGCCCTCTG ATTTTCCAGT CTGACCACTT CGGATTATCC CGTGACAGGT CATTCAGACT
4201 GGCTAATGCA CCCAGTAAGG CAGCGGTATC ATCAACAGGC TTACCCGTCT TACTGTCCCT
//
```

```
LOCUS       mJAG101                 4223 bp ds-DNA     circular     28-JUL-2022
DEFINITION  .
FEATURES             Location/Qualifiers
     terminator      complement(9..103)
                     /label="lambda t0 terminator"
     CDS             complement(115..906)
                     /label="specR"
     misc_feature    1069..1102
                     /label="J23101 mutant (Weak Constitutive Promoter)"
     RBS             1122..1134
                     /label="BBa_B0032 (RBS)"
     CDS             1136..1795
                     /label="CmR"
     misc_feature    1796..1856
                     /label="Terminator L3S2P21 (synthetic, strong)"
     terminator      1880..1977
                     /label="rrnB T1 terminator"
     CDS             complement(2523..3470)
                     /label="repA"
     rep_origin      complement(3854..4215)
                     /label="pSC101 ori"
ORIGIN
        1 GTGCTTGGAT TCTCACCAAT AAAAAACGCC CGGCGGCAAC CGAGCGTTCT GAACAAATCC
       61 AGATGGAGTT CTGAGGTCAT TACTGGATCT ATCAACAGGA GTCCAAGCGA GCTCTTATTT
      121 GCCGACTACC TTGGTGATCT CGCCTTTCAC GTAGTGGACA AATTCTTCCA ACTGATCTGC
      181 GCGCGAGGCC AAGCGATCTT CTTCTTGTCC AAGATAAGCC TGTCTAGCTT CAAGTATGAC
      241 GGGCTGATAC TGGGCCGGCA GGCGCTCCAT TGCCCAGTCG GCAGCGACAT CCTTCGGCGC
      301 GATTTTGCCG GTTACTGCGC TGTACCAAAT GCGGGACAAC GTAAGCACTA CATTTCGCTC
      361 ATCGCCAGCC CAGTCGGGCG GCGAGTTCCA TAGCGTTAAG GTTTCATTTA GCGCCTCAAA
      421 TAGATCCTGT TCAGGAACCG GATCAAAGAG TTCCTCCGCC GCTGGACCTA CCAAGGCAAC
      481 GCTATGTTCT CTTGCTTTTG TCAGCAAGAT AGCCAGATCA ATGTCGATCG TGGCTGGCTC
      541 GAAGATACCT GCAAGAATGT CATTGCGCTG CCATTCTCCA AATTGCAGTT CGCGCTTAGC
      601 TGGATAACGC CACGGAATGA TGTCGTCGTG CACAACAATG GTGACTTCTA CAGCGCGGAG
      661 AATCTCGCTC TCTCCAGGGG AAGCCGAAGT TTCCAAAAGG TCGTTGATCA AAGCTCGCCG
      721 CGTTGTTTCA TCAAGCCTTA CGGTCACCGT AACCAGCAAA TCAATATCAC TGTGTGGCTT
      781 CAGGCCGCCA TCCACTGCGG AGCCGTACAA ATGTACGGCC AGCAACGTCG GTTCGAGATG
      841 GCGCTCGATG ACGCCAACTA CCTCTGATAG TTGAGTCGAT ACTTCGGCGA TCACCGCTTC
      901 CCTCATGCGA AACGATCCTC ATCCTGTCTC TTGATCAGAT ATTGATCCCC TGCGCCATCA
      961 GATCCTTGGC GGCAAGAAAG CCATCCAGTT TACTTTGCAG GGCTTCCCAA CCTTACCAGA
     1021 GGGCGCCCCA GCTGGCAATT CCGACGTCgc ggccgcgaaG ACATAGGGtt tacagctagc
     1081 tcagtcctag gtatatgcta gcAGCGATAA CAAACTTTGA CTCACACAGG AAAGAATGGA
     1141 GAAAAAAATC ACTGGATATA CCACCGTTGA TATATCCCAA TGGCATCGTA AAGAACATTT
     1201 TGAGGCATTT CAGTCAGTTG CTCAATGTAC CTATAACCAG ACCGTTCAGC TGGATATTAC
     1261 GGCCTTTTTA AAGACCGTAA AGAAAAATAA GCACAAGTTT TATCCGGCCT TTATTCACAT
     1321 TCTTGCCCGC CTGATGAATG CTCATCCGGA ATTTCGTATG GCAATGAAAG ACGGTGAGCT
     1381 GGTGATATGG GATAGTGTTC ACCCTTGTTA CACCGTTTTC CATGAGCAAA CTGAAACGTT
     1441 TTCATCGCTC TGGAGTGAAT ACCACGACGA TTTCCGGCAG TTTCTACACA TATATTCGCA
     1501 AGATGTGGCG TGTTAtGGTG AAAACCTGGC CTATTTCCCT AAAGGGTTTA TTGAGAATAT
     1561 GTTTTTCGTC TCAGCCAATC CCTGGGTGAG TTTCACCAGT TTTGATTTAA ACGTGGCCAA
     1621 TATGGACAAC TTCTTCGCCC CCGTTTTCAC CATGGGCAAA TATTATACGC AAGGCGACAA
     1681 GGTGCTGATG CCGCTGGCGA TTCAGGTTCA TCATGCCGTt TGTGATGGCT TCCATGTCGG
     1741 CAGAATGCTT AATGAATTAC AACAGTACTG CGATGAGTGG CAGGGCGGGG CGTAACTCGG
     1801 TACCAAATTC CAGAAAAGAG GCCTCCCGAA AGGGGGGCCT TTTTTCGTTT TGGTCCAAAT
     1861 GTCTTCgcgg ccgcTCTAGA GGCATCAAAT AAAACGAAAG GCTCAGTCGA AAGACTGGGC
     1921 CTTTCGTTTT ATCTGTTGTT TGTCGGTGAA CGCTCTCCTG AGTAGGACAA ATCCGCCGCC
     1981 CTAGACCTAG CTGCAGCTAG GGTACGGGTT TTGCTGCCCG CAAACGGGCT GTTCTGGTGT
     2041 TGCTAGTTTG TTATCAGAAT CGCAGATCCG GCTTCAGccg GTTTGCCGGC TGAAAGCGCT
     2101 ATTTCTTCCA GAATTGCCAT GATTTTTTCC CCACGGGAGG CGTCACTGGC TCCCGTGTTG
     2161 TCGGCAGCTT TGATTCGATA AGCAGCATCG CCTGTTTCAG GCTGTCTATG TGTGACTGTT
     2221 GAGCTGTAAC AAGTTGTCTC AGGTGTTCAA TTTCATGTTC TAGTTGCTTT GTTTTACTGG
     2281 TTTCACCTGT TCTATTAGGT GTTACATGCT GTTCATCTGT TACATTGTCG ATCTGTTCAT
     2341 GGTGAACAGC TTTgAATGCA CCAAAAACTC GTAAAAGCTC TGATGTATCT ATCTTTTTTA
```

```
2401 CACCGTTTTC ATCTGTGCAT ATGGACAGTT TTCCCTTTGA TATgTAACGG TGAACAGTTG
2461 TTCTACTTTT GTTTGTTAGT CTTGATGCTT CACTGATAGA TACAAGAGCC ATAAGAACCT
2521 CAGATCCTTC CGTATTTAGC CAGTATGTTC TCTAGTGTGG TTCGTTGTTT TTGCGTGAGC
2581 CATGAGAACG AACCATTGAG ATCATaCTTA CTTTGCATGT CACTCAAAAA TTTTGCCTCA
2641 AAACTGGTGA GCTGAATTTT TGCAGTTAAA GCATCGTGTA GTGTTTTTCT TAGTCCGTTA
2701 tGTAGGTAGG AATCTGATGT AATGGTTGTT GGTATTTTGT CACCATTCAT TTTTATCTGG
2761 TTGTTCTCAA GTTCGGTTAC GAGATCCATT TGTCTATCTA GTTCAACTTG GAAAATCAAC
2821 GTATCAGTCG GGCGGCCTCG CTTATCAACC ACCAATTTCA TATTGCTGTA AGTGTTTAAA
2881 TCTTTACTTA TTGGTTTCAA AACCCATTGG TTAAGCCTTT TAAACTCATG GTAGTTATTT
2941 TCAAGCATTA ACATGAACTT AAATTCATCA AGGCTAATCT CTATATTTGC CTTGTGAGTT
3001 TTCTTTTGTG TTAGTTCTTT TAATAACCAC TCATAAATCC TCATAGAGTA TTTGTTTTCA
3061 AAAGACTTAA CATGTTCCAG ATTATATTTT ATGAATTTTT TTAACTGGAA AAGATAAGGC
3121 AATATCTCTT CACTAAAAAC TAATTCTAAT TTTTCGCTTG AGAACTTGGC ATAGTTTGTC
3181 CACTGGAAAA TCTCAAAGCC TTTAACCAAA GGATTCCTGA TTTCCACAGT TCTCGTCATC
3241 AGCTCTCTGG TTGCTTTAGC TAATACACCA TAAGCATTTT CCCTACTGAT GTTCATCATC
3301 TGAGCGTATT GGTTATAAGT GAACGATACC GTCCGTTCTT TCCTTGTAGG GTTTTCAATC
3361 GTGGGGTTGA GTAGTGCCAC ACAGCATAAA ATTAGCTTGG TTTCATGCTC CGTTAAGTCA
3421 TAGCGACTAA TCGCTAGTTC ATTTGCTTTG AAAACAACTA ATTCAGACAT ACATCTCAAT
3481 TGGTCTAGGT GATTTTAATC ACTATACCAA TTGAGATGGG CTAGTCAATG ATAATTACTA
3541 GTCCTTTTCC TTTGAGTTGT GGGTATCTGT AAATTCTGCT AGACCTTTGC TGGAAAACTT
3601 GTAAATTCTG CTAGACCCTC TGTAAATTCC GCTAGACCTT TGTGTGTTTT TTTTGTTTAT
3661 ATTCAAGTGG TTATAATTTA TAGAATAAAG AAAGAATAAA AAAAGATAAA AAGAATAGAT
3721 CCCAGCCCTG TGTATAACTC ACTACTTTAG TCAGTTCCGC AGTATTACAA AAGGATGTCG
3781 CAAACGCTGT TTGCTCCTCT ACAAAACAGA CCTTAAAACC CTAAAGGCTT AAGTAGCACC
3841 CTCGCAAGCT CGGGCAAATC GCTGAATATT CCTTTTGTCT CCGACCATCA GGCACCTGAG
3901 TCGCTGTCTT TTTCGTGACA TTCAGTTCGC TGCGCTCACG GCTCTGGCAG TGAATGGGGG
3961 TAAATGGCAC TACAGGCGCC TTTTATGGAT TCATGCAAGG AAACTACCCA TAATACAAGA
4021 AAAGCCCGTC ACGGGCTTCT CAGGGCGTTT TATGGCGGGT CTGCTATGTG GTGCTATCTG
4081 ACTTTTTGCT GTTCAGCAGT TCCTGCCCTC TGATTTTCCA GTCTGACCAC TTCGGATTAT
4141 CCCGTGACAG GTCATTCAGA CTGGCTAATG CACCCAGTAA GGCAGCGGTA TCATCAACAG
4201 GCTTACCCGT CTTACTGTCC CTA
//
```

```
LOCUS       mJAG019                2240 bp ds-DNA     circular     28-JUL-2022
DEFINITION  .
FEATURES             Location/Qualifiers
     terminator            complement(51..145)
                           /label="lambda t0 terminator"
     Antibiotic Resistance complement(175..1035)
                           /label="AmpR"
     misc_feature          1189..1192
                           /label="Overhang D1"
     terminator            1193..1321
                           /label="B0015 (Terminator)"
     misc_feature          1322..1325
                           /label="Overhang G"
     terminator            1348..1445
                           /label="rrnB T1 terminator"
     rep_origin            complement(1583..2226)
                           /label="colE1 ori"
ORIGIN
        1 AGTGGAACGA AAACTCACGT TAAGGGATTT TGGTCATGAC TAGTGCTTGG ATTCTCACCA
       61 ATAAAAAACG CCCGGCGGCA ACCGAGCGTT CTGAACAAAT CCAGATGGAG TTCTGAGGTC
      121 ATTACTGGAT CTATCAACAG GAGTCCAAGC GAGCTCGTAA ACTTGGTCTG ACAGTTACCA
      181 ATGCTTAATC AGTGAGGCAC CTATCTCAGC GATCTGTCTA TTTCGTTCAT CCATAGTTGC
      241 CTGACTCCCC GTCGTGTAGA TAACTACGAT ACGGGAGGGC TTACCATCTG GCCCCAGTGC
      301 TGCAATGATA CCGCGcgaCC CACGCTCACC GGCTCCAGAT TTATCAGCAA TAAACCAGCC
      361 AGCCGGAAGG GCCGAGCGCA GAAGTGGTCC TGCAACTTTA TCCGCCTCCA TCCAGTCTAT
      421 TAATTGTTGC CGGGAAGCTA GAGTAAGTAG TTCGCCAGTT AATAGTTTGC GCAACGTTGT
      481 TGCCATTGCT ACAGGCATCG TGGTGTCACG CTCGTCGTTT GGTATGGCTT CATTCAGCTC
      541 CGGTTCCCAA CGATCAAGGC GAGTTACATG ATCCCCCATG TTGTGCAAAA AAGCGGTTAG
      601 CTCCTTCGGT CCTCCGATCG TTGTCAGAAG TAAGTTGGCC GCAGTGTTAT CACTCATGGT
      661 TATGGCAGCA CTGCATAATT CTCTTACTGT CATGCCATCC GTAAGATGCT TTTCTGTGAC
      721 TGGTGAGTAC TCAACCAAGT CATTCTGAGA ATAGTGTATG CGGCGACCGA GTTGCTCTTG
      781 CCCGGCGTCA ATACGGGATA ATACCGCGCC ACATAGCAGA ACTTTAAAAG TGCTCATCAT
      841 TGGAAAACGT TCTTCGGGGC GAAAACTCTC AAGGATCTTA CCGCTGTTGA GATCCAGTTC
      901 GATGTAACCC ACTCGTGCAC CCAACTGATC TTCAGCATCT TTTACTTTCA CCAGCGTTTC
      961 TGGGTGAGCA AAAACAGGAA GGCAAAATGC CGCAAAAAAG GGAATAAGGG CGACACGGAA
     1021 ATGTTGAATA CTCATACTCT TCCTTTTTCA ATATTATTGA AGCATTTATC AGGGTTATTG
     1081 TCTCATGAGC GGATACATAT TTGAATGTAT TTAGAAAAAT AAACAAATAG GGGTTCCGCG
     1141 CACATTTCCC CGAAAAGTGC CACCTGACGT CGcggccgcA GGGTCTCAGT AACCaggcat
     1201 caaataaaac gaaaggctca gtcgaaagac tgggcctttc gttttatctg ttgtttgtcg
     1261 gtgaacgctc tctactagag tcacactggc tcaccttcgg gtgggccttt ctgcgtttat
     1321 aGACATGAGA CCAAgcggcc gcTCTAGAGG CATCAAATAA AACGAAAGGC TCAGTCGAAA
     1381 GACTGGGCCT TTCGTTTTAT CTGTTGTTTG TCGGTGAACG CTCTCCTGAG TAGGACAAAT
     1441 CCGCCGCCCT AGACCTAGCT GCAGCTAGGG CGTTCGGCTG CGGCGAGCGG TATCAGCTCA
     1501 CTCAAAGGCG GTAATACGGT TATCCACAGA ATCAGGGGAT AACGCAGGAA AGAACATGTG
     1561 AGCAAAAGGC CAGCAAAAGG CCAGGAACCG TAAAAAGGCC GCGTTGCTGG CGTTTTTCCA
     1621 TAGGCTCCGC CCCCCTGACG AGCATCACAA AAATCGACGC TCAAGTCAGA GGTGGCGAAA
     1681 CCCGACAGGA CTATAAAGAT ACCAGGCGTT TCCCCCTGGA AGCTCCCTCG TGCGCTCTCC
     1741 TGTTCCGACC CTGCCGCTTA CCGGATACCT GTCCGCCTTT CTCCCTTCGG GAAGCGTGGC
     1801 GCTTTCTCAt aGCTCACGCT GTAGGTATCT CAGTTCGGTG TAGGTCGTTC GCTCCAAGCT
     1861 GGGCTGTGTG CACGAACCCC CCGTTCAGCC CGACCGCTGC GCCTTATCCG GTAACTATCG
     1921 TCTTGAGTCC AACCCGGTAA GACACGACTT ATCGCCACTG GCAGCAGCCA CTGGTAACAG
     1981 GATTAGCAGA GCGAGGTATG TAGGCGGTGC TACAGAGTTC TTGAAGTGGT GGCCTAACTA
     2041 CGGCTACACT AGAAGGACAG TATTTGGTAT CTGCGCTCTG CTGAAGCCAG TTACCTTCGG
     2101 AAAAAGAGTT GGTAGCTCTT GATCCGGCAA ACAAACCACC GCTGGTAGCG GTGGTTTTTT
     2161 TGTTTGCAAG CAGCAGATTA CGCGCAGAAA AAAAGGATCT CAAGAAGATC CTTTGATCTT
     2221 TTCTACGGGG TCTGACGCTC
//
```

```
LOCUS       mJAG168                3254 bp ds-DNA     circular     28-JUL-2022
DEFINITION  .
FEATURES             Location/Qualifiers
     terminator      81..186
                     /label="lambda T0 terminator"
     CDS             complement(194..985)
                     /label="specR"
     misc_feature    1136..1139
                     /label="Overhang A"
     misc_feature    1140..1173
                     /label="J23101 mutant (Weak Constitutive Promoter)"
     misc_feature    1196..1199
                     /label="Overhang B1"
     RBS             1215..1225
                     /label="B0033 (RBS)"
     CDS             1232..1891
                     /label="CmR"
     misc_feature    1892..1952
                     /label="Terminator L3S2P21 (synthetic, strong)"
     terminator      1969..2066
                     /label="rrnB T1 terminator"
     rep_origin      2533..3220
                     /label="p15A ori"
ORIGIN
        1 ATATTACTAG ATTTCAGTGC AATTTATCTC TTCAAATGTA GCACCTGAAG TCAGCCCCAT
       61 ACGATATAAG TTGTTACTAG TGCTTGGATT CTCACCAATA AAAAACGCCC GGCGGCAACC
      121 GAGCGTTCTG AACAAATCCA GATGGAGTTC TGAGGTCATT ACTGGATCTA TCAACAGGAG
      181 TCCAAGCGAG CTCTTATTTG CCGACTACCT TGGTGATCTC GCCTTTCACG TAGTGGACAA
      241 ATTCTTCCAA CTGATCTGCG CGCGAGGCCA AGCGATCTTC TTCTTGTCCA AGATAAGCCT
      301 GTCTAGCTTC AAGTATGACG GGCTGATACT GGGCCGGCAG GCGCTCCATT GCCCAGTCGG
      361 CAGCGACATC CTTCGGCGCG ATTTTGCCGG TTACTGCGCT GTACCAAATG CGGGACAACG
      421 TAAGCACTAC ATTTCGCTCA TCGCCAGCCC AGTCGGGCGG CGAGTTCCAT AGCGTTAAGG
      481 TTTCATTTAG CGCCTCAAAT AGATCCTGTT CAGGAACCGG ATCAAAGAGT TCCTCCGCCG
      541 CTGGACCTAC CAAGGCAACG CTATGTTCTC TTGCTTTTGT CAGCAAGATA GCCAGATCAA
      601 TGTCGATCGT GGCTGGCTCG AAGATACCTG CAAGAATGTC ATTGCGCTGC CATTCTCCAA
      661 ATTGCAGTTC GCGCTTAGCT GGATAACGCC ACGGAATGAT GTCGTCGTGC ACAACAATGG
      721 TGACTTCTAC AGCGCGGAGA ATCTCGCTCT CTCCAGGGGA AGCCGAAGTT TCCAAAAGGT
      781 CGTTGATCAA AGCTCGCCGC GTTGTTTCAT CAAGCCTTAC GGTCACCGTA ACCAGCAAAT
      841 CAATATCACT GTGTGGCTTC AGGCCGCCAT CCACTGCGGA GCCGTACAAA TGTACGGCCA
      901 GCAACGTCGG TTCGAGATGG CGCTCGATGA CGCCAACTAC CTCTGATAGT TGAGTCGATA
      961 CTTCGGCGAT CACCGCTTCC CTCATGCGAA ACGATCCTCA TCCTGTCTCT TGATCAGATA
     1021 TTGATCCCCT GCGCCATCAG ATCCTTGGCG GCAAGAAAGC CATCCAGTTT ACTTTGCAGG
     1081 GCTTCCCAAC CTTACCAGAG GGCGCCCCAG CTGGCAATTC CGACGTCGcg gccgcAGGGt
     1141 ttacagctag ctcagtccta ggtatatgct agcAAATTGT GAGCGGATAA CAATTATAGa
     1201 taacaaactt tgactcacac aggacTACTA AATGGAGAAA AAAATCACTG GATATACCAC
     1261 CGTTGATATA TCCCAATGGC ATCGTAAAGA ACATTTTGAG GCATTTCAGT CAGTTGCTCA
     1321 ATGTACCTAT AACCAGACCG TTCAGCTGGA TATTACGGCC TTTTTAAAGA CCGTAAAGAA
     1381 AAATAAGCAC AAGTTTTATC CGGCCTTTAT TCACATTCTT GCCCGCCTGA TGAATGCTCA
     1441 TCCGGAATTT CGTATGGCAA TGAAAGACGG TGAGCTGGTG ATATGGGATA GTGTTCACCC
     1501 TTGTTACACC GTTTTCCATG AGCAAACTGA AACGTTTTCA TCGCTCTGGA GTGAATACCA
     1561 CGACGATTTC CGGCAGTTTC TACACATATA TTCGCAAGAT GTGGCGTGTT AtGGTGAAAA
     1621 CCTGGCCTAT TTCCCTAAAG GGTTTATTGA GAATATGTTT TTCGTCTCAG CCAATCCCTG
     1681 GGTGAGTTTC ACCAGTTTTG ATTTAAACGT GGCCAATATG GACAACTTCT TCGCCCCCGT
     1741 TTTCACCATG GGCAAATATT ATACGCAAGG CGACAAGGTG CTGATGCCGC TGGCGATTCA
     1801 GGTTCATCAT GCCGTtTGTG ATGGCTTCCA TGTCGGCAGA ATGCTTAATG AATTACAACA
     1861 GTACTGCGAT GAGTGGCAGG GCGGGGCGTA ACTCGGTACC AAATTCCAGA AAAGAGGCCT
     1921 CCCGAAAGGG GGCCTTTTTT TCGTTTTGGT CCAAgcggcc gcCCTAGGAG GCATCAAATA
     1981 AAACGAAAGG CTCAGTCGAA AGACTGGGCC TTTCGTTTTA TCTGTTGTTT GTCGGTGAAC
     2041 GCTCTCCTGA GTAGGACAAA TCCGCCGCCC TAGACCTAGC TGCAGCTAGG GTACGGGTTT
     2101 TGCTGCCCGC AAACGGGCTG TTCTGGTGTT GCTAGTTTGT TATCAGAATC GCAGATCCGG
     2161 CTTCAGccgG TTTGCCGGCT GAAAGCGCTA TTTCTTCCAG AATTGCCATG ATTTTTTCCC
     2221 CACGGGAGGC GTCACTGGCT CCCGTGTTGT CGGCAGCTTT GATTCGATAA GCAGCATCGC
```

```
2281 CTGTTTCAGG CTGTCTATGT GTGACTGTTG AGCTGTAACA AGTTGTCTCA GGTGTTCAAT
2341 TTCATGTTCT AGTTGCTTTG TTTTACTGGT TTCACCTGTT CTATTAGGTG TTACATGCTG
2401 TTCATCTGTT ACATTGTCGA TCTGTTCATG GTGAACAGCT TTgAATGCAC CAAAAACTCG
2461 TAAAAGCTCT GATGTATCTA TCTTTTTTAC ACCGTTTTCA TCTGTGcatg cAcgaagcgg
2521 cgcgCCCATA TGGGATATAT TCCGCTTCCT CGCTCACTGA CTCGCTACGC TCGGTCGTTC
2581 GACTGCGGCG AGCGGAAATG GCTTACGAAC GGGGCGGAGA TTTCCTGGAA GATGCCAGGA
2641 AGATACTTAA CAGGGAAGTG AGAGGGCCGC GGCAAAGCCG TTTTTCCATA GGCTCCGCCC
2701 CCCTGACAAG CATCACGAAA TCTGACGCTC AAATCAGTGG TGGCGAAACC CGACAGGACT
2761 ATAAAGATAC CAGGCGTTTC CCCCTGGCGG CTCCCTCGTG CGCTCTCCTG TTCCTGCCTT
2821 TCGGTTTACC GGTGTCATTC CGCTGTTATG GCCGCGTTTG TCTCATTCCA CGCCTGACAC
2881 TCAGTTCCGG GTAGGCAGTT CGCTCCAAGC TGGACTGTAT GCACGAACCC CCCGTTCAGT
2941 CCGACCGCTG CGCCTTATCC GGTAACTATC GTCTTGAGTC CAACCCGGAA AGACATGCAA
3001 AAGCACCACT GGCAGCAGCC ACTGGTAATT GATTTAGAGG AGTTAGTCTT GAAGTCATGC
3061 GCCGGTTAAG GCTAAACTGA AAGGACAAGT TTTGGTGACT GCGCTCCTCC AAGCCAGTTA
3121 CCTCGGTTCA AAGAGTTGGT AGCTCAGAGA ACCTTCGAAA AACCGCCCTG CAAGGCGGTT
3181 TTTTCGTTTT CAGAGCAAGA GATTACGCGC AGACCAAAAC GATCTCAAGA AGATCATCTT
3241 ATTAATCAGA TAAA
//
```

References

[1] B. G. Wong, C. P. Mancuso, S. Kiriakov, C. J. Bashor, A. S. Khalil, Precise, automated control of conditions for high-throughput growth of yeast and bacteria with eVOLVER. *Nature Biotechnology* **36**, 614–623 (2018).

[2] F. Bertaux, S. Sosa-Carrillo, V. Gross, A. Fraisse, C. Aditya, M. Furstenheim, G. Batt, Enhancing bioreactor arrays for automated measurements and reactive control with ReacSight. *Nature Communications* **13**, 1–12 (2022).

[3] E. Martinez-Salas, J. A. Martin, M. Vicente, Relationship of Escherichia coli density to growth rate and cell age. *Journal of Bacteriology* **147**, 97 (1981).

[4] P. P. Dennis, H. Bremer, Modulation of Chemical Composition and Other Parameters of the Cell at Different Exponential Growth Rates. *EcoSal Plus* **3** (2008).

[5] M. Scott, C. W. Gunderson, E. M. Mateescu, Z. Zhang, T. Hwa, Interdependence of Cell Growth and Gene Expression: Origins and Consequences. *Science* **330**, 1099–1102 (2010).

[6] M. Scott, S. Klumpp, E. M. Mateescu, T. Hwa, Emergence of robust growth laws from optimal regulation of ribosome synthesis. *Molecular Systems Biology* **10**, 747 (2014).

[7] C. You, H. Okano, S. Hui, Z. Zhang, M. Kim, C. W. Gunderson, Y.-P. Wang, P. Lenz, D. Yan, T. Hwa, Coordination of bacterial proteome with metabolism by cyclic AMP signalling. *Nature* **500**, 301–6 (2013).

[8] J. B. Deris, M. Kim, Z. Zhang, H. Okano, R. Hermsen, A. Groisman, T. Hwa, The innate growth bistability and fitness landscapes of antibiotic-resistant bacteria. *Science* **342** (2013).

[9] D. W. Erickson, S. J. Schink, V. Patsalo, J. R. Williamson, U. Gerland, T. Hwa, A global resource allocation strategy governs growth transition kinetics of Escherichia coli. *Nature* **551**, 119–123 (2017).

[10] O. Borkowski, F. Ceroni, G. B. Stan, T. Ellis, Overloaded and stressed: whole-cell considerations for bacterial synthetic biology. *Current Opinion in Microbiology* **33**, 123–130 (2016).

[11] F. Ceroni, R. Algar, G.-B. Stan, T. Ellis, Quantifying cellular capacity identifies gene expression designs with reduced burden. *Nature methods* **12**, 1–8 (2015).

[12] A. Y. Weiße, D. A. Oyarzún, V. Danos, P. S. Swain, Mechanistic links between cellular trade-offs, gene expression, and growth. *Proceedings of the National Academy of Sciences of the United States of America* **112**, E1038-E1047 (2015).

[13] R. Balakrishnan, M. Mori, I. Segota, Z. Zhang, R. Aebersold, C. Ludwig, T. Hwa, Principles of gene regulation quantitatively connect DNA to RNA and proteins in bacteria. *bioRxiv* p. 2021.05.24.445329 (2021).

[14] C. Orelle, E. D. Carlson, T. Szal, T. Florin, M. C. Jewett, A. S. Mankin, Protein synthesis by ribosomes with tethered subunits. *Nature* **524**, 119–124 (2015).

[15] R. Milo, P. Jorgensen, U. Moran, G. Weber, M. Springer, BioNumbers—the database of key numbers in molecular and cell biology. *Nucleic Acids Research* **38**, D750 (2010).

[16] A. Baumschlager, S. K. Aoki, M. Khammash, Dynamic Blue Light-Inducible T7 RNA Polymerases (Opto-T7RNAPs) for Precise Spatiotemporal Gene Expression Control. *ACS Synthetic Biology* **6**, 2157–2167 (2017).
