# Supplementary material for: Dynamic cybergenetic control of bacterial co-culture composition via optogenetic feedback
Source: Nat Commun. 2022 Aug 16;13:4808. doi: 10.1038/s41467-022-32392-z (PMC9381578; doi:10.1038/s41467-022-32392-z)
Supplement: Supplementary file 2 — Reporting Summary [file 41467_2022_32392_MOESM2_ESM.pdf]

nature portfolio

Corresponding author(s): Prof. Dr. Mustafa Khammash

Last updated by author(s): Jul 25, 2022

Reporting Summary

Nature Portfolio wishes to improve the reproducibility of the work that we publish. This form provides structure for consistency and transparency in reporting. For further information on Nature Portfolio policies, see our Editorial Policies and the Editorial Policy Checklist.

Statistics

For all statistical analyses, confirm that the following items are present in the figure legend, table legend, main text, or Methods section.

n/a	Confirmed	
☐	☒	The exact sample size (*n*) for each experimental group/condition, given as a discrete number and unit of measurement
☐	☒	A statement on whether measurements were taken from distinct samples or whether the same sample was measured repeatedly
☒	☐	The statistical test(s) used AND whether they are one- or two-sided<br>*Only common tests should be described solely by name; describe more complex techniques in the Methods section.*
☒	☐	A description of all covariates tested
☒	☐	A description of any assumptions or corrections, such as tests of normality and adjustment for multiple comparisons
☐	☒	A full description of the statistical parameters including central tendency (e.g. means) or other basic estimates (e.g. regression coefficient) AND variation (e.g. standard deviation) or associated estimates of uncertainty (e.g. confidence intervals)
☒	☐	For null hypothesis testing, the test statistic (e.g. *F*, *t*, *r*) with confidence intervals, effect sizes, degrees of freedom and *P* value noted<br>*Give P values as exact values whenever suitable.*
☒	☐	For Bayesian analysis, information on the choice of priors and Markov chain Monte Carlo settings
☒	☐	For hierarchical and complex designs, identification of the appropriate level for tests and full reporting of outcomes
☒	☐	Estimates of effect sizes (e.g. Cohen's *d*, Pearson's *r*), indicating how they were calculated

Our web collection on statistics for biologists contains articles on many of the points above.

Software and code

Policy information about availability of computer code

Data collection	eVOLVER version 2.0 (Fynch Bio) for acquisition of OD and growth rate data.<br>OT-2 Robot (Opentrons); Server version: 4.3.1; Firmware Version: v1.1.0-25e5cea; Protocol API version: 2.0<br>Microsoft Visual Studio 2017 IDE<br>Python v3.7.1<br>Python v3.6.8<br>Arduino IDE v1.8.12<br>CytExpert v2.4 API - flow cytometry samples acquisition.<br>MATLAB R2021a (Mathworks)
Data analysis	Custom python (v.3.8.6) script for gating and analysis of fcs files.<br>Custom python (v.3.8.6) script for analysis of growth rate measurement files from eVOLVER.<br>Custom MATLAB R2019b (Mathworks) scripts were used for plotting and figures were then formated using Inkscape (v0.92, open source).

For manuscripts utilizing custom algorithms or software that are central to the research but not yet described in published literature, software must be made available to editors and reviewers. We strongly encourage code deposition in a community repository (e.g. GitHub). See the Nature Portfolio guidelines for submitting code & software for further information.

Data

Policy information about availability of data

All manuscripts must include a data availability statement. This statement should provide the following information, where applicable:

- Accession codes, unique identifiers, or web links for publicly available datasets
- A description of any restrictions on data availability
- For clinical datasets or third party data, please ensure that the statement adheres to our policy

All data required to evaluate the conclusions in the paper are present in the main text and/or the Supplementary Materials. Source Data for all the figures presented in the paper is made available in a Source Data .zip folder.
The plasmids used in this study were all designed using publictly-available sequences and will be shared upon reasonable request.

Human research participants

Policy information about studies involving human research participants and Sex and Gender in Research.

Reporting on sex and gender	N/A
Population characteristics	N/A
Recruitment	N/A
Ethics oversight	N/A

Note that full information on the approval of the study protocol must also be provided in the manuscript.

Field-specific reporting

Please select the one below that is the best fit for your research. If you are not sure, read the appropriate sections before making your selection.

[x] Life sciences [ ] Behavioural & social sciences [ ] Ecological, evolutionary & environmental sciences

For a reference copy of the document with all sections, see nature.com/documents/nr-reporting-summary-flat.pdf

Life sciences study design

All studies must disclose on these points even when the disclosure is negative.

Sample size	Sample were collected in biologically independent triplicates, as described below. We chose this number of samples since we observed that the results of the triplicates were very consistent, with no outliers, and therefore it represented a good compromise between statistical significance and experimental effort.
Data exclusions	No data was excluded.
Replication	All experiments were performed in biologically independent triplicates, either on the same day from different overnight cultures or on different days. The three replicates resulted in consistent and reproducible results with only small SEM values. Therefore, we deemed that three independent experiments were sufficient to provide a good estimate of both the steady-state growth rates of the strains in characterization experiments and the dynamic behavior of those strains, both in isolation and in co-culture. The characterization experiments of the slowest constitutive strains were only performed twice, since these strains were not relevant for the rest of the study. Also, to show that the setpoint can be changed during a closed-loop run, we performed two individual experiments with distinct dynamic setpoint profiles. We did not perform replicates of these experiments for the following reason: These experiments are only slight variations of the closed-loop experiments with a single setpoint, for which we present three biologically independent runs. Moreover, the resulting dynamic profiles are consistent, both among the two experiments with different setpoint profiles, as well as with the dynamics observed in the three closed-loop experiments with a single high or low setpoint respectively, showing that the results of closed-loop experiments in general are consistent and reproducible.
Randomization	The cultures were observed to have slightly different growth rates depending on the particular eVOLVER sleeve in which the vial is incubated. This effect probably arises from different alignments in the magnets that drive the bars responsible for stirring, resulting in different degrees of oxygenation. To ensure that no systematic errors were accumulated due to this factor, the sleeve positions in the eVOLVER were randomized for the growth-rate characterization experiments and open-loop and closed-loop repeats.
Blinding	Knowledge of sample identity does not affect interpretation of experimental data.

Reporting for specific materials, systems and methods

We require information from authors about some types of materials, experimental systems and methods used in many studies. Here, indicate whether each material, system or method listed is relevant to your study. If you are not sure if a list item applies to your research, read the appropriate section before selecting a response.

Materials & experimental systems

n/a	Involved in the study	
☒	☐	Antibodies
☒	☐	Eukaryotic cell lines
☒	☐	Palaeontology and archaeology
☒	☐	Animals and other organisms
☒	☐	Clinical data
☒	☐	Dual use research of concern

Methods

n/a	Involved in the study	
☒	☐	ChIP-seq
☐	☒	Flow cytometry
☒	☐	MRI-based neuroimaging

Flow Cytometry

Plots

Confirm that:

☒ The axis labels state the marker and fluorochrome used (e.g. CD4-FITC).

☒ The axis scales are clearly visible. Include numbers along axes only for bottom left plot of group (a 'group' is an analysis of identical markers).

☒ All plots are contour plots with outliers or pseudocolor plots.

☒ A numerical value for number of cells or percentage (with statistics) is provided.

Methodology

Sample preparation	Samples were transferred directly from the turbidostat vials of the eVOLVER to a sampling vial on the flow cytometer. Then, 10ul of sample volume were recorded with a time limit of 1min. A detailed protocol of the different steps in the automated sampling process, including washing steps and waste removal, is available in the supplementary material.
Instrument	Cytoflex S (Beckman Coulter)
Software	CytExpert v2.4 software
Cell population abundance	Between 90-98% of events fell within the morphological gates described below and were then separated into a photophilic or constitutive fraction according to a threshold gate on the GFP-A channel
Gating strategy	Gains: mVenus was measured with a 488-nm laser and 525/40 bandpass filter, and mCherry was measured with 561-nm laser and 610/20 bandpass filter. The gain settings were as follows: forward scatter 100, side scatter 100, GFP 500, PE 145, mCherry 500.<br><br>Gating: Both in automated closed-loop experiments and in the custom python scripts used for analysis of the data from characterization experiments, a first polygon gate was applied on the FCS-H vs. SSC-H channels to select for living cells (P2). P2 was then further gated in the SSC-Width vs. FSC-H channels to select for single cells (P4). In co-culture experiments, the P4 population was further separated into two subpopulations (corresponding to the photophilic and constitutive strains) by applying a fixed threshold gat on the GFP-A channel (Threshold=6500).

☒ Tick this box to confirm that a figure exemplifying the gating strategy is provided in the Supplementary Information.
