# Supplementary material for: Dynamic cybergenetic control of bacterial co-culture composition via optogenetic feedback
Source: Nat Commun. 2022 Aug 16;13:4808. doi: 10.1038/s41467-022-32392-z (PMC9381578; doi:10.1038/s41467-022-32392-z)
Supplement: Supplementary file 4 — Description of Additional Supplementary Files [file 41467_2022_32392_MOESM4_ESM.pdf]

Title: Supplementary Software

Description: MATLAB scripts used to run simulations of the photophilic-constitutive co-culture, both in open-loop and closed-loop settings, as well as the optimization pipeline to determine the best PID gains for a closed-loop co-culture experiment.
